# Which practices co‐deliver food security, climate change mitigation and adaptation, and combat land degradation and desertification?*

**DOI:** 10.1111/gcb.14878

**Published:** 2019-12-14

**Authors:** Pete Smith, Katherine Calvin, Johnson Nkem, Donovan Campbell, Francesco Cherubini, Giacomo Grassi, Vladimir Korotkov, Anh Le Hoang, Shuaib Lwasa, Pamela McElwee, Ephraim Nkonya, Nobuko Saigusa, Jean‐Francois Soussana, Miguel Angel Taboada, Frances C. Manning, Dorothy Nampanzira, Cristina Arias‐Navarro, Matteo Vizzarri, Jo House, Stephanie Roe, Annette Cowie, Mark Rounsevell, Almut Arneth

**Affiliations:** ^1^ Institute of Biological & Environmental Sciences University of Aberdeen Aberdeen UK; ^2^ Pacific Northwest National Laboratory Joint Global Change Research Institute College Park MD USA; ^3^ United Nations Economic Commission for Africa Addis Ababa Ethiopia; ^4^ The University of the West Indies Mona Jamaica; ^5^ Industrial Ecology Programme Department of Energy and Process Engineering Norwegian University of Science and Technology (NTNU) Trondheim Norway; ^6^ European Commission Joint Research Centre Ispra Italy; ^7^ Yu. A. Izrael Institute of Global Climate and Ecology Moscow Russia; ^8^ Ministry of Agriculture and Rural Development (MARD) Hanoi Vietnam; ^9^ Department of Geography Makerere University Kampala Uganda; ^10^ Department of Human Ecology Rutgers University New Brunswick NJ USA; ^11^ IFPRI Washington DC USA; ^12^ Center for Global Environmental Research National Institute for Environmental Studies Tsukuba Ibaraki Japan; ^13^ French National Institute for Agricultural, Environment and Food Research (INRA) Paris France; ^14^ National Agricultural Technology Institute (INTA) Natural Resources Research Center (CIRN) Institute of Soils Ciudad Autónoma de Buenos Aires Argentina; ^15^ Department of Livestock and Industrial Resources Makerere University Kampala Uganda; ^16^ School of Geographical Sciences University of Bristol Bristol UK; ^17^ Department of Environmental Sciences University of Virginia Charlottesville VA USA; ^18^ Climate Focus Berlin Germany; ^19^ NSW Department of Primary Industries DPI Agriculture Livestock Industries Centre University of New England Armidale NSW Australia; ^20^ Karlsruhe Institute of Technology, Atmospheric Environmental Research (KIT, IMK‐IFU) Garmisch‐Partenkirchen Germany; ^21^ Institute of Geography University of Edinburgh Edinburgh UK

**Keywords:** adaptation, adverse side effects, co‐benefits, demand management, desertification, food security, land degradation, land management, mitigation, practice, risk management

## Abstract

There is a clear need for transformative change in the land management and food production sectors to address the global land challenges of climate change mitigation, climate change adaptation, combatting land degradation and desertification, and delivering food security (referred to hereafter as “land challenges”). We assess the potential for 40 practices to address these land challenges and find that: Nine options deliver medium to large benefits for all four land challenges. A further two options have no global estimates for adaptation, but have medium to large benefits for all other land challenges. Five options have large mitigation potential (>3 Gt CO_2_eq/year) without adverse impacts on the other land challenges. Five options have moderate mitigation potential, with no adverse impacts on the other land challenges. Sixteen practices have large adaptation potential (>25 million people benefit), without adverse side effects on other land challenges. Most practices can be applied without competing for available land. However, seven options could result in competition for land. A large number of practices do not require dedicated land, including several land management options, all value chain options, and all risk management options. Four options could greatly increase competition for land if applied at a large scale, though the impact is scale and context specific, highlighting the need for safeguards to ensure that expansion of land for mitigation does not impact natural systems and food security. A number of practices, such as increased food productivity, dietary change and reduced food loss and waste, can reduce demand for land conversion, thereby potentially freeing‐up land and creating opportunities for enhanced implementation of other practices, making them important components of portfolios of practices to address the combined land challenges.

## Graphical abstract

There is a clear need for transformative change in the land management and food production sectors to address the global land challenges of climate change mitigation, climate change adaptation, combatting land degradation and desertification, and delivering food security (referred to hereafter as “land challenges”). We assess the potential for 40 practices to address these land challenges and find that most practices deliver across all land challenges. Some practices could result in competition for land, but those that reduce demand for land conversion potentially free‐up land, creating opportunities for enhanced implementation of other practices, making them important for addressing combined land challenge.

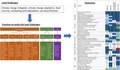

## 1 INTRODUCTION

Many of the greatest current global challenges are related to the food system and the way that land is used and managed. Among the most pressing of these global challenges are the need to mitigate and adapt to climate change, the need to combat desertification and land degradation, and the need to deliver food security. These are collectively referred to, hereafter, as the “land challenges.” Given that many of the land challenges are related, there is a clear need to facilitate transformative change in land management and food production systems to address these global land challenges (Alexander, Rounsevell, Henry, Reddy, & Brown, 2019; Reed & Stringer, 2016; Webb et al., 2017).

**Figure 1:**
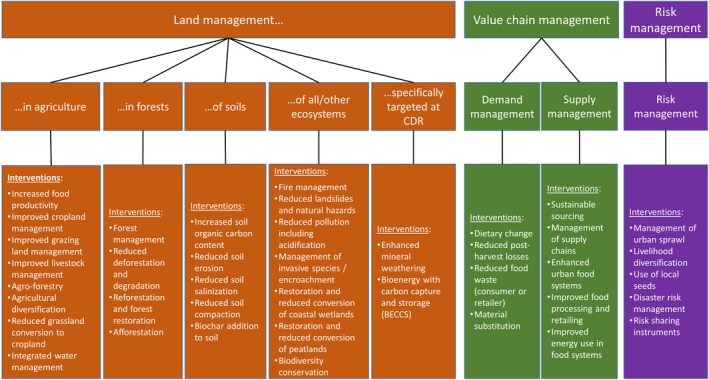
Broad categorization of practices categorized into three main classes and eight subclasses

A number of practices have been suggested to address one or more of these land challenges. The practices considered in this study can be categorized into those that rely on (a) land management; (b) value chain management; and (c) risk management (Figure 1). The land management practices can be grouped according to those that are applied in agriculture, in forests, on soils, in other/all ecosystems and those that are applied specifically for carbon dioxide removal (CDR). There is overlap in the categories; for example, the soil‐based strategies can be applied in agricultural or forestry systems; feedstock production for bioenergy can be an agricultural or forestry activity. The value chain management practices can be categorized as those based on demand management and supply management. The risk management options are grouped together (Figure 1).

In this paper, we assess quantitatively at the global scale the potential of 40 practices (Figure 1, lower boxes) for addressing each of these land challenges (note that food security is largely addressed from an undernutrition perspective), in order to identify those that deliver across more than one challenge, and those that can result in trade‐offs across the land challenges.

## 2 MATERIALS AND METHODS

Practices available to address the land challenges of climate change mitigation, climate change adaptation, land degradation, desertification, and food security were collated from the literature. These practices are described in Tables 1, 2, 3 for land management‐based, value chain management‐based, and risk management‐based practices, respectively, with context caveats and supporting references provided in Tables S1–S3.

**Table 1:** Land management‐based practices considered in this study

Practice	Description
Increased food productivity	Increased food productivity arises when the output of food commodities increases per unit of input, for example, per unit of land or water. It can be realized through many other practices such as improved cropland, grazing land, and livestock management
Improved cropland management	Improved cropland management is a collection of practices consisting of (a) *management of the crop*: including high carbon input practices, for example, improved crop varieties, crop rotation, use of cover crops, perennial cropping systems, integrated production systems, crop diversification, agricultural biotechnology; (b) *nutrient management*: including optimized fertilizer application rate, fertilizer type (organic manures, compost, and mineral), timing, precision application, nitrification inhibitors; (c) *reduced tillage intensity and residue retention*; (d) *improved water management*: including drainage of waterlogged mineral soils and irrigation of crops in arid/ semiarid conditions; (e) *improved rice management*: including water management such as mid‐season drainage and improved fertilization and residue management in paddy rice systems; and (f) *biochar application*
Improved grazing land management	Improved grazing land management is a collection of practices consisting of (a) *management of vegetation*: including improved grass varieties/sward composition, deep rooting grasses, increased productivity, and nutrient management; (b) *animal management*: including appropriate stocking densities fit to carrying capacity, fodder banks, and fodder diversification; and (c) *fire management*: improved use of fire for sustainable grassland management, including fire prevention and improved prescribed burning (see also fire management as a separate practice below)
Improved livestock management	Improved livestock management is a collection of practices consisting of (a) *improved feed and dietary additives* (e.g., bioactive compounds, fats), used to increase productivity and reduce emissions from enteric fermentation; (b) *breeding* (e.g., breeds with higher productivity or reduced emissions from enteric fermentation); (c) *herd management,* including decreasing neonatal mortality, improving sanitary conditions, animal health and herd renewal, and diversifying animal species; (d) *emerging technologies* (of which some are not legally authorized in several countries) such as propionate enhancers, nitrate and sulfate supplements, archaea inhibitors and archaeal vaccines, methanotrophs, acetogens, defaunation of the rumen, bacteriophages and probiotics, ionophores/antibiotics; and (e) *improved manure management*, including manipulation of bedding and storage conditions, anaerobic digesters; biofilters, dietary change and additives, soil‐applied and animal‐fed nitrification inhibitors, urease inhibitors, fertilizer type, rate and timing, manipulation of manure application practices, and grazing management
Agroforestry	Agroforestry involves the deliberate planting of trees in croplands and silvopastoral systems
Agricultural diversification	Agricultural diversification includes a set of agricultural practices that aim to improve the resilience of farming systems to climate variability and climate change and to economic risks posed by fluctuating market forces. In general, the agricultural system is shifted from one based on low‐value agricultural commodities to one that is more diverse, composed of a basket of higher value‐added products
Reduced grassland conversion to cropland	Grasslands can be converted to croplands by plowing of grassland and seeding with crops. Since croplands have a lower soil carbon content than grasslands and are also more prone to erosion than grasslands, reducing conversion of grassland to croplands will prevent soil carbon losses by oxidation and soil loss through erosion. These processes can be reduced if the rate of grassland conversion to cropland is reduced
Integrated water management	Integrated water management is the process of creating holistic strategies to promote integrated, efficient, equitable, and sustainable use of water for agroecosystems. It includes a collection of practices including water‐use efficient irrigation in arid/semiarid areas, improvement of soil water holding capacity through increases in soil organic matter content, and improved cropland management, agroforestry, and conservation agriculture. Increasing water availability, and reliability of water for agricultural production, achieved by using different techniques of water harvesting, storage, and its judicious utilization through farm ponds, dams, and community tanks in rainfed agriculture areas can benefit adaptation
Improved and sustainable forest management	Improved forest management refers to management practices in forests for the purpose of climate change mitigation. It includes a wide variety of practices affecting the growth of trees and the biomass removed, including improved regeneration (natural or artificial) and a better schedule, intensity, and execution of operations (thinning, selective logging, final cut; reduced impact logging, etc.). Sustainable forest management is the stewardship and use of forests and forest lands in a way, and at a rate, that maintains their biodiversity, productivity, regeneration capacity, vitality, and their potential to fulfill, now and in the future, relevant ecological, economic, and social functions, at local, national, and global levels, and that does not cause damage to other ecosystems
Reduced deforestation and degradation	Reduced deforestation and forest degradation include conservation of existing carbon pools in forest vegetation and soil by controlling the drivers of deforestation (i.e., commercial and subsistence agriculture, mining, urban expansion) and forest degradation (i.e., overharvesting including fuelwood collection, poor harvesting practices, overgrazing, pest outbreaks, and extreme wildfires), also through establishing protected areas, improving law enforcement, forest governance and land tenure, supporting community forest management, and introducing forest certification
Reforestation and forest restoration	Reforestation is the conversion to forest of land that has previously contained forests but that has been converted to some other use. Forest restoration refers to practices aimed at regaining ecological integrity in a deforested or degraded forest landscape. As such, it could fall under reforestation if it were reestablishing trees where they have been lost, or under forest management if it were restoring forests where not all trees have been lost. For practical reasons, here forest restoration is treated together with reforestation
Afforestation	Afforestation is the conversion to forest of land that historically has not contained forests (see also reforestation)
Increased soil organic carbon content	Practices that increase soil organic matter content include (a) *land use change* to an ecosystem with higher equilibrium soil carbon levels (e.g., from cropland to forest); (b) *management of the vegetation*: including high carbon input practices, for example, improved varieties, rotations and cover crops, perennial cropping systems, biotechnology to increase inputs and recalcitrance of below ground carbon; (c) *nutrient management and organic material input* to increase carbon returns to the soil: including optimized fertilizer and organic material application rate, type, timing, and precision application; (d) *reduced tillage intensity and residue retention*; and (e) *improved water management*: including irrigation in arid/semiarid conditions
Reduced soil erosion	Soil erosion is the removal of soil from the land surface by water, wind, or disturbance, which occurs worldwide but it is particularly severe in Asia, Latin America, and the Caribbean, and the Near East and North Africa. Soil erosion management includes conservation practices (e.g., the use of minimum tillage or zero tillage, crop rotations and cover crops, rational grazing systems), engineering‐like practices (e.g., construction of terraces and contour banks for controlling water erosion), or forest barriers and strip cultivation for controlling wind erosion. In eroded soils, the advance of erosion gullies and sand dunes can be limited by increasing plant cover, among other practices
Reduced soil salinization	Soil salinization is a major process of land degradation that decreases soil fertility and affects agricultural production, aquaculture, and forestry. It is a significant component of desertification processes in drylands. Practices to reduce soil salinization include improvement of water management (e.g., water‐use efficiency and irrigation/drainage technology in arid/semiarid areas, surface and groundwater management), improvement of soil health (through increase in soil organic matter), and improved cropland, grazing land and livestock management, agroforestry, and conservation agriculture
Reduced soil compaction	Reduced soil compaction mainly includes agricultural techniques (e.g., crop rotations with deep‐rooted thesis, control of livestock density) and control of agricultural traffic
Biochar addition to soil	The use of biochar, a solid product of the pyrolysis process, as a soil amendment can increase the water‐holding and nutrient‐holding capacity of soil and can stabilize added organic matter. It may therefore provide better access to water and nutrients for crops and other vegetation types (as part of cropland, grazing land, and improved forest management). The sourcing for feedstock for pyrolysis also needs to be considered
Fire management	Fire management is a land management option aimed at safeguarding life, property, and resources through the prevention, detection, control, restriction, and suppression of fire in forest and other vegetation. It includes the improved use of fire for sustainable forestry management, including wildfire prevention and prescribed burning. Prescribed burning is used to reduce the risk of large, uncontrollable fires in forest areas. Controlled burning is an effective economic method of reducing fire danger and stimulating natural reforestation under the forest canopy and after clear felling
Reduced landslides and natural hazards	Landslides are mainly triggered by human activity (e.g., legal and illegal mining, fire, deforestation) in combination with climate change. Management of landslides and natural hazards (e.g., floods, storm surges, droughts) is based on vegetation management (e.g., afforestation) and engineering works (e.g., dams, terraces, stabilization, and filling of erosion gullies)
Reduced pollution including acidification	Management of air pollution is connected to climate change by emission sources of air polluting materials and their impacts on climate, human health, and ecosystems, including agriculture. Acid deposition is one of the many consequences of air pollution, harming trees, and other vegetation, as well as contributing to land degradation. Practices that reduce acid deposition include prevention of emissions of nitrogen oxides (NO*_x_*) and sulfur dioxide (SO_2_), which also reduce GHG emissions and other short‐lived climate pollutants (SLCPs). Reductions of SLCPs reduce warming in the near term and the overall rate of warming, which can be crucial for plants that are sensitive to even small increases in temperature. Management of harmful air pollutants such as fine particulate matter (PM2.5) and ozone (O_3_) also mitigates the impacts of incomplete fossil fuel combustion and GHG emissions. In addition, management of pollutants such as tropospheric O_3_ has beneficial impacts on food production, since O_3_ decreases crop production. Control of urban and industrial air pollution also mitigate the harmful effects of pollution and provide benefits via improved human health. Management of pollution contributes to aquatic ecosystem conservation as controlling air pollution, rising atmospheric CO_2_ concentrations, acid deposition, and industrial waste reduce acidification of marine and freshwater ecosystems
Management of invasive species/encroachment	Agriculture and forests can be diverse but often, much of the diversity is non‐native. Invasive species in different biomes have been introduced intentionally or unintentionally through export of ornamental plants or animals, and through the promotion of modern agriculture and forestry. Non‐native species tend to be more numerous in some human‐modified landscapes (e.g., over 50% of species in an urbanized area or extensive agricultural fields can be non‐native). Invasive alien species in the United States cause major environmental damage amounting to almost US$120 billion year^−1^. There are approximately 50,000 foreign species and the number is increasing. About 42% of the species on the threatened or endangered species lists are at risk primarily because of alien‐invasive species. Invasive species can be managed through manual clearance of invasive species, while in some areas, natural enemies of the invasive species are introduced to control them
Restoration and reduced conversion of coastal wetlands	Coastal wetland restoration involves restoring degraded/ damaged coastal wetlands including mangroves, salt marshes, and seagrass ecosystems
Restoration and reduced conversion of peatlands	Peatland restoration involves restoring degraded/damaged peatlands, through rewetting, which both increases carbon sinks, but also avoids ongoing CO_2_ emissions from degraded peatlands, so it both prevents future emissions and creates a sink, as well as protecting biodiversity
Biodiversity conservation	Biodiversity conservation refers to practices aiming at maintaining components of biological diversity. It includes conservation of ecosystems and natural habitats, maintenance and recovery of viable populations of species in their natural surroundings (in situ conservation) and, in the case of domesticated or cultivated species, in the surroundings where they have developed their distinctive properties outside their natural habitats (ex situ conservation). Examples of biodiversity conservation measures are establishment of protected areas to achieve specific conservation objectives, preservation of biodiversity hotspots, land management to recover natural habitats, practices to expand or control targeted plant or animal species in productive lands or rangelands (e.g., rewilding), sustainable harvest of native species
Enhanced weathering of minerals	The enhanced weathering of minerals that naturally absorb CO_2_ from the atmosphere has been proposed as a CDR technology with a large mitigation potential. The rocks are ground to increase the surface area and the ground minerals are then applied to the land where they absorb atmospheric CO_2_
Bioenergy and BECCS	Bioenergy production can mitigate climate change by delivering an energy service, therefore avoiding combustion of fossil energy. It is the most common renewable energy source used today in the world and has a large potential for future deployment. BECCS entails the use of bioenergy technologies (e.g., bioelectricity or biofuels) in combination with CO_2_ capture and storage. BECCS simultaneously provides energy and can reduce atmospheric CO_2_ concentrations; thus, BECCS is considered a CDR technology. While several BECCS demonstration projects exist, it has yet to be deployed at scale. Bioenergy and BECCS are widely used in many future scenarios as a climate change mitigation option in the energy and transport sector, especially those scenarios aimed at a stabilization of global climate at 2°C or less above pre‐industrial levels

Note

Context and supporting references are provided in Table S1.

Abbreviation: BECCS, bioenergy with carbon capture and strorage; CDR, carbon dioxide removal; GHG, greenhouse gas.

**Table 2:** Value chain management‐based practices considered in this study

Practice	Description
Dietary change	Sustainable healthy diets represent a range of dietary changes to improve human diets, to make them healthy in terms of the nutrition delivered, and also (economically, environmentally, and socially) sustainable. A “contract and converge” model of transition to sustainable healthy diets would involve a reduction in overconsumption (particularly of livestock products) in overconsuming populations, with increased consumption of some food groups in populations where minimum nutritional needs are not met. Such a conversion could result in a decline in undernourishment, as well as reduction in the risk of morbidity and mortality due to overconsumption
Reduced post‐harvest losses	Approximately one‐third of the food produced for human consumption is wasted in post‐production operations. The key drivers for post‐harvest waste in developing countries are structural and infrastructure deficiencies, requiring responses that process, preserve, and, where appropriate, redistribute food to where it can be consumed immediately
Reduced food waste	Food loss in developed countries mostly occurs at the retail/consumer stage, and practices that focuses on consumer or retailer waste (ranging from better use by date labeling to consumer education campaigns) can reduce pressure on land (see also reducing post‐harvest losses above)
Material substitution	Material substitution involves the use of wood or agricultural biomass (e.g., straw bales) instead of fossil fuel‐based materials (e.g., concrete, iron, steel, aluminum) for building, textiles, or other applications
Sustainable sourcing	Sustainable sourcing includes approaches to ensure that the production of goods is done in a sustainable way, such as through low‐impact agriculture, zero deforestation supply chains, or sustainably harvested forest products. Currently around 8% of global forest area has been certified in some manner, and 25% of global industrial roundwood comes from certified forests. Sustainable sourcing can also enable producers to increase their percentage of the final value of commodities through improved innovation, coordination, and efficiency in supply chains, as well as labeling to ensure consumer demands. Promoting sustainable and value‐added products can reduce the need for compensatory extensification of agricultural areas and is a specific commitment of some sourcing programs (such as forest certification programs)
Management of supply chains	Management of supply chains include improving efficiency and sustainability to reduce climate risk and profitably reduce emissions and can include: (a) increasing the economic value through improved production processes; (b) adopting emission accounting tools (e.g., carbon and water footprinting); (c) improved policies for stability of food supply to minimize food price volatility
Enhanced urban food systems	Urban areas are becoming the principal territories for practice in improving food access through innovative strategies that aim to reduce hunger and improve livelihoods, including support for urban and peri‐urban agriculture, green infrastructure (e.g., green roofs), local markets, enhanced social (food) safety nets, development of alternative food sources and technologies, such as vertical farming, and local food policy and planning initiatives. Such systems have created nutritious food supplies for the city, while improving the health status of urban dwellers, reducing pollution levels, adapting to and mitigating climate change, and stimulating economic development
Improved food processing and retailing	Improved food processing and retailing involves several practices related to improving packaging, processing, cooling, drying, and extracting, and reducing agri‐food GHG emissions from processing and transportation and reducing waste in retailing
Improved energy use in food systems	Energy efficiency of agriculture can be improved to reduce the dependency on nonrenewable energy sources either by decreased energy inputs, or through increased outputs per unit of input. In some countries, managerial inefficiency (rather than a technology gap) is the main source for energy efficiency loss. Heterogenous patterns of energy efficiency exist at the national scale and promoting energy efficient technologies along with managerial capacity development can reduce the gap and provide large benefits for climate adaptation. Improvements in carbon monitoring and calculation techniques such as the foot‐printing of agricultural products can enhance energy efficiency transition management and uptake in agricultural enterprises

Note

Context and supporting references are provided in Table S2.

**Table 3:** Risk management‐based practices considered in this study

Practice	Description
Management of urban sprawl	Unplanned urban expansion of cities along the rural–urban fringe (especially strong in emerging towns and cities in Asia and Africa) has been identified as a driver of forest and agricultural land loss and a threat to food production around cities and may result in a 1.8%–2.4% loss of global croplands by 2030. Policies to prevent urban sprawl have included integrated land use planning, agricultural zoning ordinances and agricultural districts, urban redevelopment, arable land reclamation, and transfer/purchase of development rights or easements
Livelihood diversification	Livelihood diversification (drawing from a portfolio of dissimilar sources of livelihood as a tool to spread risk) has been identified as one option to increase incomes and reduce poverty, increase food security, and promote climate resilience and risk reduction
Use of local seeds	Using local seeds (also called seed sovereignty) refers to use of non‐improved, non‐commercial seed varieties. These can be used and stored by local farmers as low‐cost inputs and can often help contribute to the conservation of local varieties and landraces, increasing local biodiversity, and often require no pesticide or fertilizer use, leading to less land degradation
Disaster risk management	Disaster risk management encompasses many approaches to try to reduce the consequences of climate and weather‐related disasters and events on socioeconomic systems through proactive prevention; timely response; quick and effective recovery; and sustainable development. Other options include using early warning systems that can encompass (a) education systems; (b) hazard and risk maps; (c) hydrological and meteorological monitoring (such as flood forecasting or extreme weather warnings); and (d) communication systems
Risk sharing instruments	Risk sharing instruments can encompass a variety of approaches, including intra‐household risk pooling, community rotating credit associations (ROSCAs) and other formal and informal credit services, as well as insurance of various kinds. Commercial crop insurance can involve both traditional indemnity‐based insurance that reimburses clients for estimated financial losses from shortfalls, or index insurance that pays out the value of an index (such as weather events) rather than actual losses

Note

Context and supporting references are provided in Table S3.

Practices often overlap, so are not additive. For example, increasing food productivity will involve changes to cropland, grazing land, and livestock management, which in turn may include increasing soil carbon stocks. The practices cannot therefore be summed and are not mutually exclusive (e.g., cropland management might also increase soil organic matter stocks), and some of the practices considered comprise a few potential management interventions (e.g., improved cropland management is a collection of management interventions). Enabling conditions and strategies such as use of indigenous and local knowledge, attention to gender issues, appropriate governance, etc., are not categorized as practices, so are not included in this analysis. Some suggested methods to address land challenges are better described as *overarching frameworks* than as practices. For example, *climate smart agriculture* is a collection of practices aimed at delivering mitigation and adaptation in agriculture, including improved cropland management, grazing land management, and livestock management. Similarly, policy goals, such as *land degradation neutrality*, include a number of practices. For this reason, policy goals or overarching frameworks (see Table S4) are not treated as practices in this study, but their component practices are.

The IPCC SR1.5 (2018) considered a range of practices (from a mitigation/adaptation perspective only). Table S5 shows how the IPCC SR1.5 options map on to the practices considered in this study. Note that this study excludes most of the energy‐related options from IPCC SR1.5, as well as green infrastructure and sustainable aquaculture.

A comprehensive literature review was conducted to gather evidence on the quantitative impact of the practices on each land challenge. The quantified global potential of each practice was then compared to thresholds for each land challenge to assess whether the positive or negative potential was large, moderate, or small. The thresholds for categorization of potentials are shown in Table 4. No equivalence is implied in terms of positive or negative impacts, either in the number or in the magnitude of the impact, that is, one benefit *does not equal* one adverse side effect. As a consequence, (a) large benefits for one land challenge might outweigh relatively minor negative impacts in addressing another land challenge; (b) some practices may deliver mostly benefits with few negative impacts, but the benefits might be small in magnitude, that is, the practices do no harm, but present only minor co‐benefits; and (c) the lack of *global* estimates of potential does not imply there is no evidence of impact; regional studies often show impacts of the practices, but if the global impact is not available of the literature or cannot be inferred from published studies, no value is given.

**Table 4:** Criteria used to define magnitude of impact of each practice

	Mitigation	Adaptation	Desertification	Land degradation	Food
Large positive	More than 3 Gt CO_2_eq/year	Positively impacts more than around 25 million people	Positively impacts more than around 3 million km^2^	Positively impacts more than around 3 million km^2^	Positively impacts more than around 100 million people
Moderate positive	0.3–3 Gt CO_2_eq	1 million to 25 million	0.5–3 million km^2^	0.5–3 million km^2^	1 million to 100 million
Small positive	>0	Under 1 million	>0	>0	Under 1 million
Negligible	0	No effect	No effect	No effect	No effect
Small negative	<0	Under 1 million	<0	<0	Under 1 million
Moderate negative	−0.3 to −3 Gt CO_2_eq	1 million to 25 million	0.5–3 million km^2^	0.5–3 million km^2^	1 million to 100 million
Large negative	More than −3 Gt CO_2_eq/year	Negatively impacts more than around 25 million people	Negatively impacts more than around 3 million km^2^	Negatively impacts more than around 3 million km^2^	Negatively impacts more than around 100 million people

Note

Magnitudes are for the technical potential of practices globally. For each land challenge, magnitudes are set relative to a marker level as follows. For mitigation, potentials are set relative to the approximate potentials for the mitigation options with the largest individual impacts (~3 Gt CO_2_eq/year; Pacala and Socolow, 2004). The threshold for the “large” magnitude category is set at this level. For adaptation, magnitudes are set relative to the 100 million lives estimated to be affected by climate change and a carbon‐based economy between 2010 and 2030 (DARA, 2012). The threshold for the “large” magnitude category represents 25% of this total. For desertification and land degradation, magnitudes are set relative to the lower end of current estimates of degraded land, 10–60 million km^2^ (Gibbs & Salmon, 2015). The threshold for the “large” magnitude category represents 30% of the lower estimate. For food security, magnitudes are set relative to the approximately 800 million people who are currently undernourished (HLPE, 2017). The threshold for the “large” magnitude category represents 12.5% of this total.

## 3 RESULTS

In the sections below, we provide the quantitative estimates/ranges for the global potential for each practice to address the land challenges of climate change mitigation (Section 3.1), climate change adaptation (Section 3.2), land degradation and desertification (Section 3.3), and food security (Section 3.4) arising from the extensive literature review, before summarizing these potentials in relation to the thresholds in Table 4, across all land challenges.

### 3.1 Potential of the practices for delivering mitigation

#### 3.1.1 Practices based on land management

*Increasing the productivity of land* used for food production can deliver significant mitigation by avoiding emissions that would occur if increased food demand were met through expansion of the agricultural land area (Burney, Davis, & Lobell, 2010). If pursued through injudicious use of agrochemical inputs, numerous adverse impacts on greenhouse gas (GHG) emissions and other aspects of environmental sustainability can occur (Table 5), but if pursued sustainably and with appropriate governance and other measures to prevent rebound effects, for example, through sustainable intensification (e.g., Pretty et al., 2018), increased food productivity could provide high levels of mitigation. For example, yield improvement has been estimated to have contributed to emissions' savings of >13 Gt CO_2_eq/year since 1961 (Burney et al., 2010; Table 5). If the considerable remaining global yield gaps (Mueller et al., 2012) could be closed through sustainable intensification, mitigation of a similar magnitude could be realized. This can also reduce the GHG intensity of products (Bennetzen, Smith, & Porter, 2016a, 2016b) which means a smaller environmental footprint of production, since demand can be met using less land and/or with fewer animals.

**Table 5:** Summary of global mitigation effects of practices based on land management

Practice	Potential	Confidence	Citation
Increased food productivity	>13 Gt CO_2_eq/year	Low confidence	Burney et al. (2010)
Improved cropland management	1.4–2.3 Gt CO_2_eq/year	Medium confidence	Smith et al. (2008, 2014), Pradhan et al. (2013)
Improved grazing land management	1.4–1.8 Gt CO_2_eq/year	Medium confidence	Conant, Cerri, Osborne, and Paustian (2017), Herrero et al. (2016), Smith et al. (2008, 2014)
Improved livestock management	0.2–2.4 Gt CO_2_eq/year	Medium confidence	Herrero et al. (2016), Smith et al. (2008, 2014)
Agroforestry	0.1–5.7 Gt CO_2_eq/year	Medium confidence	Dickie et al. (2014), Griscom et al. (2017), Hawken (2017), Zomer et al. (2016)
Agricultural diversification	>0	Low confidence	Campbell et al. (2014), Cohn et al. (2017)
Reduced grassland conversion to cropland	0.03–0.7 Gt CO_2_eq/year	Low confidence	Calculated from values in Griscom et al. (2017), Krause et al. (2017), Poeplau et al. (2011)
Integrated water management	0.1–0.72 Gt CO_2_eq/year	Low confidence	Howell, Evett, Tolk, Copeland, and Marek (2015), IPCC (2014), Li et al. (2006), Rahman and Bulbul (2015), Smith et al. (2008, 2014)
Improved and sustainable forest management	0.4–2.1 Gt CO_2_eq/year	Medium confidence	Bastin et al. (2019), Griscom et al. (2017), Sasaki et al. (2016)
Reduced deforestation and degradation	0.4–5.8 Gt CO_2_eq/year	High confidence	Baccini et al. (2017), Griscom et al. (2017), Hawken (2017), Houghton, Byers, and Nassikas (2015), Houghton and Nassikas (2018), Smith et al. (2014)
Reforestation and forest restoration	1.5–10.1 Gt CO_2_eq/year	Medium confidence	Dooley and Kartha (2018), Griscom et al. (2017), Hawken (2017), Houghton and Nassikas (2018)
Afforestation	See Reforestation	Medium confidence	Fuss et al. (2018), Hawken (2017), Kreidenweis et al. (2016), Lenton (2010)
Increased soil organic carbon content	0.4–8.6 Gt CO_2_eq/year	High confidence	Conant et al. (2017), Dickie et al. (2014), Frank et al. (2017), Fuss et al. (2018), Griscom et al. (2017), Hawken (2017), Henderson et al. (2015), Herrero et al. (2016), Lal (2004), McLaren (2012), Paustian et al. (2016), Poeplau and Don (2015), Powlson et al. (2014), Sanderman et al. (2017), Smith (2016), Sommer and Bossio (2014), Zomer et al. (2016)
Reduced soil erosion	Source of 1.36–3.67 to sink of 0.44–3.67 Gt CO_2_eq/year	Low confidence	Jacinthe and Lal (2001), Lal (2004), Smith et al. (2001, 2005), Stallard (1998), Van Oost et al. (2007)
Reduced soil salinization	>0	Low confidence	Dagar et al. (2016), UNCTAD (2011), Wong et al. (2010)
Reduced soil compaction	>0	Low confidence	Chamen et al. (2015), Epron et al. (2016), Tullberg et al. (2018)
Biochar addition to soil	0.03–6.6 Gt CO_2_eq/year	Medium confidence	Dickie et al. (2014), Fuss et al. (2018), Griscom et al. (2017), Hawken (2017), IPCC (2018), Lenton (2010, 2014), Powell and Lenton (2012), Pratt and Moran (2010), Roberts, Gloy, Joseph, Scott, and Lehmann (2009), Smith (2016), Woolf et al. (2010)
Fire management	0.48–8.1 Gt CO_2_eq/year	Medium confidence	Arora and Melton (2018), Tacconi (2016)
Reduced landslides and natural hazards	>0	Low confidence	
Reduced pollution including acidification	(a) Reduce projected warming ~0.5°C by 2050; (b) reduce terrestrial C uptake 0.55–1.28 Gt CO_2_eq/year	(a) and (b) medium confidence	(a) Shindell et al. (2012); UNEP and WMO (2011); (b) Bala et al. (2013)
Management of invasive species/encroachment	No global estimates	No evidence	
Restoration and reduced conversion of coastal wetlands	0.3–3.1 Gt CO_2_eq/year	Medium confidence	Donato et al. (2011), Griscom et al. (2017), Hawken (2017), Howard et al. (2017), Pendleton et al. (2012)
Restoration and reduced conversion of peatlands	0.6–2 Gt CO_2_eq/year	Medium confidence	Couwenberg et al. (2010), Griscom et al. (2017), Hawken (2017), Hooijer et al. (2010), Joosten and Couwenberg (2008)
Biodiversity conservation	~0.9 Gt CO_2_eq/year	Low confidence	Calvin et al. (2014), Schmitz et al. (2014)
Enhanced weathering of minerals	0.5–4 Gt CO_2_eq/year	Medium confidence	Beerling et al., 2018, Lenton (2010), Smith, Davis, et al. (2016), Taylor et al. (2016)
Bioenergy and BECCS	0.4–11.3 Gt CO_2_eq/year	Medium confidence	IPCC SR1.5; Fuss et al. (2018), Lenton (2010, 2014), McLaren (2012), Powell and Lenton (2012)

Note

The land management‐based mitigation ranges are consistent with those of Roe et al. (2019).

Abbreviation: BECCS, bioenergy with carbon capture and strorage.

*Improved cropland management* could provide moderate levels of mitigation (1.4–2.3 Gt CO_2_eq/year; Pradhan, Reusser, & Kropp, 2013; Smith et al., 2008, 2014; Table 5). The lower estimate of potential is from Pradhan et al. (2013) for decreasing emissions intensity, and the upper end of technical potential is estimated by adding technical potentials for cropland management (about 1.4 Gt CO_2_eq/year), rice management (about 0.2 Gt CO_2_eq/year), and restoration of degraded land (about 0.7 Gt CO_2_eq/year) from Smith et al. (2008, 2014). Note that much of this potential arises from soil carbon sequestration, so there is overlap with that practice.

Grazing lands can store large stocks of carbon in soil and root biomass (Conant & Paustian, 2002; O'Mara, 2012; Zhou et al., 2017). The global mitigation potential for *improved grazing land management* is moderate (1.4–1.8 Gt CO_2_eq/year), with the lower value in the range for technical potential taken from Smith et al. (2008), which includes only grassland management measures, and the upper value in the range from Herrero et al. (2016), which also includes indirect effects and some components of livestock management, and soil carbon sequestration, so there is overlap with these practices. Conant, Paustian, Del Grosso, and Parton (2005) caution that increases in soil carbon stocks could be partially offset by increases in N_2_O fluxes.

The mitigation potential of *improved livestock management* is also moderate (0.2–1.8 Gt CO_2_eq/year; Smith et al. (2008) including only direct livestock measures; Herrero et al. (2016) include also indirect effects, and some components of grazing land management and soil carbon sequestration) to high (6.1 Gt CO_2_eq/year; Pradhan et al., 2013; Table 5), and the higher estimates overlap with other practices.

Zomer et al. (2016) reported that trees in *agroforestry* landscapes have increased carbon stocks equivalent to 0.7 Gt CO_2_eq/year. Estimates of global potential range from 0.1 to 5.7 Gt CO_2_eq/year (from an “optimum implementation” scenario of Hawken, 2017), based on an assessment of all values in Dickie et al. (2014), Griscom et al. (2017), Hawken (2017), and Zomer et al. (2016).

*Agricultural diversification* mainly aims to increase climate resilience, but it may have a small (but globally unquantified) mitigation potential as a function of crop type, fertilizer management, tillage system, and soil type (Campbell, Thornton, Zougmoré, Asten, & Lipper, 2014; Cohn et al., 2017).

*Reducing conversion of grassland to cropland* could provide significant climate change mitigation by retaining soil carbon stocks that might otherwise be lost. When grasslands are converted to croplands, they lose on average 36% of their soil organic carbon stocks after 20 years (Poeplau et al., 2011). Assuming an average starting soil organic carbon stock of grasslands of 115 t C/ha (Poeplau et al., 2011), this is equivalent to a loss of 41.5 t C/ha on conversion to cropland. Mean annual global cropland conversion rates (1961–2003) have been around 47,000 km^2^/year (Krause et al., 2017) or 940,000 km^2^ over a 20 year period. The equivalent loss of soil organic carbon over 20 years would, therefore, be 14 Gt CO_2_eq = 0.7 Gt CO_2_eq/year. Griscom et al. (2017) estimate a cost‐effective mitigation potential of 0.03 Gt CO_2_eq/ year (Table 5).

*Integrated water management* provides moderate benefits for climate change mitigation through interactions with other land management strategies. For example, promoting soil carbon conservation (e.g., reduced tillage) can improve the water retention capacity of soils. Jat et al. (2015) found that improved tillage practices and residue incorporation increased water‐use efficiency by 30%, rice–wheat yields by 5%–37%, income by 28%–40%, and reduced GHG emissions by 16%–25%. While irrigated agriculture accounts for only 20% of the total cultivated land, the energy consumption from groundwater irrigation is significant. However, current estimates of mitigation potential are limited to reductions in GHG emissions mainly in cropland and rice cultivation (Smith et al., 2008, 2014). Li, Xu, Tiwari, and Ji (2006) estimated a 0.52–0.72 Gt CO_2_eq/year reduction using alternate wetting and drying practices. Current estimates of N_2_O release from terrestrial soils and wetlands account for 10%–15% of anthropogenically fixed nitrogen on the Earth system (Wang et al., 2017).

*Improved and sustainable forest management* could potentially contribute to moderate mitigation benefits globally, up to about 2 Gt CO_2_eq/year (Table 5). For managed forests, the most effective forest carbon mitigation strategy is the one that, through increasing biomass productivity, optimizes the carbon stocks (in forests and in long‐lived products) as well as the wood substitution effects for a given time frame (Erb et al., 2018; Kurz, Smyth, & Lemprière, 2016; Nabuurs, Pussinen, Brusselen, & Schelhaas, 2007; Smyth et al., 2014). Estimates of the mitigation potential also vary depending on the counterfactual, such as business‐as‐usual management (e.g., Grassi, Pilli, House, Federici, & Kurz, 2018) or other assumptions. Climate change will affect the mitigation potential of forest management due to an increase in extreme events such as fires, insects, and pathogens (Seidl et al., 2017). More detailed estimates are available at regional or biome level. For instance, according to Nabuurs et al. (2017), the implementation of Climate‐Smart Forestry (a combination of improved forest management, expansion of forest areas, energy substitution, establishment of forest reserves, etc.) in the European Union has the potential to contribute an additional 0.4 Gt CO_2_eq/year mitigation by 2050. In tropical forests, adoption of reduced impact logging and wood processing technologies along with financial incentives can reduce forest fires, forest degradation, maintain timber production, and retain carbon stocks (Sasaki et al., 2016). Forest certification may support sustainable forest management, helping to prevent forest degradation and over‐logging (Rametsteiner & Simula, 2003). Community forest management has proven a viable model for sustainable forestry, including for carbon sequestration (Chhatre & Agrawal, 2009).

*Reducing deforestation and forest degradation* rates represents one of the most effective and robust options for climate change mitigation, with large mitigation benefits globally (up to 5.8 Gt CO_2_eq/year; Table 5). Because of the combined climate impacts of GHGs and biogeophysical effects (e.g., albedo, evapotranspiration, etc.), reducing deforestation in the tropics has a major climate change mitigation effect, with benefits at local levels too (Alkama & Cescatti, 2016). Reduced deforestation and forest degradation typically lead to large co‐benefits for other ecosystem services (McElwee et al., 2019).

A large range of estimates exist in the scientific literature for the mitigation potential of *reforestation and forest restoration*, and they often overlap with estimates for afforestation. At a global level, the overall potential for these options is large (Bastin et al., 2019; Griscom et al., 2017), reaching about 10 Gt CO_2_eq/year (Table 5). The greatest potential for these options is in tropical and subtropical climates (Houghton & Nassikas, 2018; Lewis, Wheeler, Mitchard, & Koch, 2019). The climate change mitigation benefits of afforestation and reforestation are reduced at high latitudes owing to surface albedo feedback.

The global mitigation potential for *increasing soil organic matter stocks* in mineral soils is estimated to be in the range of 1.3–5.1 Gt CO_2_eq/year, although the full literature range is wider (Fuss et al., 2018; Lal, 2004; Sanderman, Hengl, & Fiske, 2017; Smith, 2016; Smith et al., 2008; Sommer & Bossio, 2014; Table 5).

The management and *control of soil erosion* may prevent losses of organic carbon in water‐ or wind‐transported sediments. However, since the final fate of eroded material is still debated, ranging from a source of 1.36–3.67 Gt CO_2_eq/year (Jacinthe & Lal, 2001; Lal, 2004) to a sink of 0.44–3.67 Gt CO_2_eq/year (Stallard, 1998; Smith, Renwick, Buddemeier, & Crossland, 2001; Smith, Sleezer, Renwick, & Buddemeier, 2005; Van Oost et al., 2007; Table 5), the overall impact of erosion control on mitigation is context specific and highly uncertain at the global level (Hoffmann et al., 2013).

*Salt‐affected soils* are highly constrained environments that require permanent prevention of salinization. Their mitigation potential is likely to be small, though prevention of salinization has more potential, though the global mitigation potential is not quantified (Dagar, Sharma, Sharma, & Singh, 2016; UNCTAD, 2011; Wong, Greene, Dalal, & Murphy, 2010).

*Preventing soil compaction* could reduce N_2_O emissions by minimizing anoxic conditions favorable for denitrification, but its carbon sequestration potential depends on crop management; the global mitigation potential, though globally unquantified, is likely to be small (Chamen, Moxey, Towers, Balana, & Hallett, 2015; Epron et al., 2016; Tullberg, Antille, Bluett, Eberhard, & Scheer, 2018; Table 5).

For *biochar*, a global analysis of technical potential, in which biomass supply constraints were applied to protect against food insecurity, loss of habitat and land degradation, estimated potential abatement of 3.7–6.6 Gt CO_2_eq/year (including 2.6–4.6 Gt CO_2_eq/year carbon stabilization). Considering all published estimates, the estimates of potential range from 0.03 to 6.6 Gt CO_2_eq/year with the lowest estimate from the “plausible” scenario of Hawken (2017; Table 5). Fuss et al. (2018) propose a range of 0.5–2 Gt CO_2_eq/year as the sustainable potential for negative emissions through biochar, similar to the range proposed by Smith (2016) and IPCC (2018).

For *fire management*, total emissions from fires have been in the order of 8.1 Gt CO_2_eq/year for the period 1997–2016 and there are important synergies between air pollution and climate change control policies. Reduction in fire CO_2_ emissions from fire suppression and landscape fragmentation associated with increases in population density is calculated to have enhanced land carbon uptake by 0.48 Gt CO_2_eq/year for the 1960–2009 period (Arora & Melton, 2018; Table 5).

*Management of landslides and natural hazards* is a key climate adaptation option, but due to limited global areas vulnerable to landslides and natural hazards, its mitigation potential is likely to be modest (Noble et al., 2014). Forest regeneration stabilizes hillsides and reduces landslides (Robledo, Fischler, & Patiño, 2004).

In terms of *management of pollution*, including acidification, UNEP and WMO (2011) and Shindell et al. (2012) identified measures targeting reduction in short‐lived climate pollutant emissions that reduce projected global mean warming by about 0.5°C by 2050. Bala, Devaraju, Chaturvedi, Caldeira, and Nemani (2013) showed that N deposition and elevated CO_2_ could have a synergistic effect, which could explain 47% of terrestrial carbon uptake in the 1990s. Estimates of global terrestrial carbon uptake due to current N deposition range from 0.55 to 1.28 Gt CO_2_eq/year (Bala et al., 2013; De Vries, Reinds, Gundersen, & Sterba, 2006; de Vries et al., 2009; Zaehle & Dalmonech, 2011; Table 5).

There are no global data on the impacts of the *management of invasive species*/encroachment on mitigation.

*Coastal wetland restoration* could provide high levels of climate mitigation, with avoided coastal wetland impacts and coastal wetland restoration estimated to deliver 0.3–3.1 Gt CO_2_eq/year in total when considering all global estimates from Griscom et al. (2017), Hawken (2017), Pendleton et al. (2012), Howard et al. (2017), and Donato et al., 2011; Table 5).

*Peatland restoration* could provide moderate levels of climate mitigation, with avoided peat impacts and peat restoration estimated to deliver 0.6–2 Gt CO_2_eq/year from all global estimates published in Couwenberg, Dommain, and Joosten (2010), Griscom et al. (2017), Hawken (2017), Hooijer et al. (2010), and Joosten and Couwenberg (2008), though in some cases, there could be an increase in methane emissions after restoration (Jauhiainen, Limin, Silvennoinen, & Vasander, 2008; Table 5).

Mitigation potential from *biodiversity conservation* varies depending on the type of practice and specific context. Protected areas are estimated to store over 300 Gt carbon, roughly corresponding to 15% of terrestrial carbon stocks (Campbell, Lobell, Genova, & Field, 2008; Kapos et al., 2008). At global level, the potential mitigation resulting from protection of these areas for the period 2005–2095 is on average about 0.9 Gt CO_2_eq/year relative to a reference scenario (Calvin et al., 2014). The potential effects on the carbon cycle of the management of wild animal species are context dependent. For example, moose browsing in boreal forests can decrease the carbon uptake of ecosystems by up to 75% (Schmitz et al., 2018), and reducing moose density through active population management in Canada is estimated to be a carbon sink equivalent to about 0.37 Gt CO_2_eq/year (Schmitz et al., 2014).

*Enhanced mineral weathering* provides substantial climate mitigation, with a global mitigation potential in the region of about 0.5–4 Gt CO_2_eq/year (Beerling et al., 2018; Lenton, 2010; Smith, House, et al., 2016; Taylor et al., 2016; Table 5).

The mitigation potential for *bioenergy and bioenergy with carbon capture and strorage* (*BECCS*) derived from bottom‐up models is large (IPCC SR1.5, 2018), with technical potential estimated at 100–300 EJ/year (IPCC, 2011) or up to ~11 Gt CO_2_eq/year. These estimates, however, exclude N_2_O associated with fertilizer application and land‐use change emissions. Those effects are included in the modeled scenarios using bioenergy and BECCS, with the magnitude depending on where the bioenergy is grown (Wise et al., 2015), at what scale, and whether N fertilizer is used.

#### 3.1.2 Practices based on value chain management

*Dietary change* and *waste reduction* can provide large benefits for mitigation, with potentials of 0.7–8 Gt CO_2_eq/year for dietary change and 0.7–4.5 Gt CO_2_eq/year for food waste reduction (Aleksandrowicz, Green, Joy, Smith, & Haines, 2016; Bajželj et al., 2014; Dickie et al., 2014; Hawken, 2017; Hedenus, Wirsenius, & Johansson, 2014; Herrero et al., 2016; Popp, Lotze‐Campen, & Bodirsky, 2010; Smith et al., 2013; Springmann et al., 2016; Stehfest et al., 2009; Tilman & Clark, 2014). Estimates for food waste reduction (Bajželj et al., 2014; Dickie et al., 2014; Hawken, 2017; Hiç, Pradhan, Rybski, & Kropp, 2016) include both consumer/retail waste and post‐harvest losses (Table 6).

**Table 6:** Summary of mitigation effects of practices based on demand management

Practice	Potential	Confidence	Citation
Dietary change	0.7–8 Gt CO_2_eq/year	High confidence	Bajželj et al. (2014), Dickie et al. (2014), Hawken (2017), Hedenus et al. (2014), Herrero et al. (2016), Popp et al. (2010), Smith et al. (2013), Springmann et al. (2016, 2018), Stehfest et al. (2009), Tilman and Clark (2014)
Reduced post‐harvest losses	4.5 Gt CO_2_eq/year	High confidence	Bajželj et al. (2014)
Reduced food waste (consumer or retailer)	0.8–4.5 Gt CO_2_eq/year	High confidence	Bajželj et al. (2014), Dickie et al. (2014), Hawken (2017), Hiç Pradhan Rybski & Kropp (2016)
Material substitution	0.25–1 Gt CO_2_eq/year	Medium confidence	Dugan et al. (2018), Gustavsson et al. (2006), Kauppi et al. (2001), Leskinen et al. (2018), McLaren (2012), Miner (2010), Sathre and O'Connor (2010), Smyth, Rampley, Lemprière, Schwab, and Kurz (2017)
Sustainable sourcing	No global estimates	No evidence	
Management of supply chains	No global estimates	No evidence	
Enhanced urban food systems	No global estimates	No evidence	
Improved food processing and retailing	See improved energy efficiency		
Improved energy use in food systems	0.37 Gt CO_2_eq/year	Low confidence	James and James (2010), Vermeulen et al. (2012)

Some studies indicate that *material substitution* has the potential for significant mitigation, with one study estimating a 14%–31% reduction in global CO_2_ emissions (Oliver, Nassar, Lippke, & McCarter, 2014); other studies suggest more modest potential (Gustavsson et al., 2006; Table 6).

While *sustainable sourcing* presumably delivers a mitigation benefit, there are no global estimates of potential. Palm oil production alone is estimated to contribute 0.038–0.045 Gt C/year, and the Indonesian palm oil expansion contributed up to 9% of tropical land use change carbon emissions in the 2000s (Carlson & Curran, 2013), but the mitigation benefit of sustainable sourcing of palm oil has not been quantified. There are no estimates of the mitigation potential for *urban food systems*.

*Efficient use of energy and resources* in food transport and distribution can contribute to a reduction in GHG emissions, estimated to be 1% of global CO_2_ emissions (James & James, 2010; Vermeulen, Campbell, & Ingram, 2012). Given that global CO_2_ emissions in 2017 were 37 Gt CO_2_eq, this equates to 0.37 Gt CO_2_eq/year (covering *food transport and distribution*, *improved efficiency of food processing and retailing*, and *improved energy efficiency*; Table 6).

#### 3.1.3 Practices based on risk management

In general, because these options are focused on adaptation and other co‐benefits, the mitigation benefits are modest, and mostly unquantified. Extensive and less dense urban development tends to have higher energy usage, particularly from transport (Liu, Zhou, & Wu, 2015), such that a 10% reduction of very low density urban fabrics is correlated with 9% fewer emissions per capita in Europe (Baur, Förster, & Kleinschmit, 2015). However, the exact contribution to mitigation from the *prevention of urban sprawl* through land conversion in particular has not been well quantified (Thornbush, Golubchikov, & Bouzarovski, 2013). Suggestions from selected studies in the United States are that biomass decreases by half when forest is converted to urban land (Briber et al., 2015), and a study in Bangkok found a decline by half in carbon sinks in the urban area in the past 30 years (Ali, Pumijumnong, & Cui, 2018).

There is no literature specifically on the linkages between *livelihood diversification* and climate mitigation benefits, although some forms of diversification that include agroforestry would likely result in increased carbon sinks (Altieri, Nicholls, Henao, & Lana, 2015; Descheemaeker et al., 2016). There is no literature exploring linkages between *use of local seeds* and GHG emission reductions.

While *disaster risk management* can presumably have mitigation co‐benefits, as it can help reduce food loss on‐farm (e.g., crops destroyed before harvest or avoided animal deaths during droughts and floods, meaning reduced production losses and wasted emissions), there is no quantified global estimate for this potential (Table 7).

**Table 7:** Summary of mitigation effects of practices based on risk management

Practice	Potential	Confidence	Citation
Management of urban sprawl	No global estimates	No evidence	
Livelihood diversification	No global estimates	No evidence	
Use of local seeds	No global estimates	No evidence	
Disaster risk management	No global estimates	No evidence	
Risk sharing instruments	>−0.024 Gt CO_2_eq/year for crop insurance; likely some benefits for other risk sharing instruments	Low confidence	Claassen et al. (2011), EPA (2018)

*Risk sharing instruments* could have some mitigation co‐benefits if they buffer household losses and reduce the need to expand agricultural lands after experiencing risks. However, the overall impacts of these are unknown. Furthermore, commercial insurance may induce producers to bring additional land into crop production, particularly marginal or land with other risks that may be more environmentally sensitive (Claassen, Cooper, & Carriazo, 2011). Policies to deny crop insurance to farmers who have converted grasslands in the United States resulted in a 9% drop in conversion, which likely had positive mitigation impacts (Claassen et al., 2011). Estimates of emissions from cropland conversion in the United States in 2016 were 23.8 Mt CO_2_e, only some of which could be attributed to insurance as a driver.

### 3.2 Potential of the practices for delivering adaptation

#### 3.2.1 Practices based on land management

*Increasing food productivity* by practices such as sustainable intensification improves farm incomes and allows households to build assets for use in times of stress, thereby improving resilience (Campbell et al., 2014). By reducing pressure on land and increasing food production, increased food productivity could be beneficial for adaptation (Campbell et al., 2014). Pretty et al. (2018) report that 163 million farms occupying 4.53 Mkm^2^ have passed a redesign threshold for application of sustainable intensification, suggesting the minimum number of people benefiting from increased productivity and adaptation benefits under sustainable intensification is >163 million, with the total likely to be far higher (Table 8).

**Table 8:** Summary of adaptation effects of practices based on land management

Practice	Potential	Confidence	Citation
Increased food productivity	>163 million people	Medium confidence	Pretty et al. (2018)
Improved cropland management	>25 million people	Low confidence	Challinor et al. (2014), Lipper et al. (2014), Lobell (2014), Vermeulen et al. (2012)
Improved grazing land management	1–25 million people	Low confidence	Porter et al. (2014)
Improved livestock management	1–25 million people	Low confidence	Porter et al. (2014), Rojas‐Downing et al. (2017)
Agroforestry	2,300 million people	Medium confidence	Lasco et al. (2014)
Agricultural diversification	>25 million people	Low confidence	Campbell et al. (2014), Cohn et al. (2017), Vermeulen et al. (2012)
Reduced grassland conversion to cropland	No global estimates	No evidence	
Integrated water management	250 million people	Low confidence	Dillon and Arshad (2016), Liu et al. (2017)
Improved and sustainable forest management	>25 million people	Low confidence	CRED (2015), World Bank et al. (2009)
Reduced deforestation and degradation	1–25 million people	Low confidence	CRED (2015), Keenan et al. (2015), World Bank et al. (2009). The estimates consider a cumulated effect to the end of the century
Reforestation and forest restoration	See afforestation		
Afforestation	>25 million people	Medium confidence	CRED (2015), Griscom et al. (2017), Reyer et al. (2009), Smith et al. (2014), Sonntag et al. (2016), World Bank, Food and Agriculture Organization and International Fund for Agricultural Development (2009). The estimates consider a cumulated effect to the end of the century
Increased soil organic carbon content	Up to 3,200 million people	Low confidence	IPBES (2018)
Reduced soil erosion	Up to 3,200 million people	Low confidence	IPBES (2018)
Reduced soil salinization	1–25 million people	Low confidence	Dagar et al. (2016), Qadir et al. (2013), UNCTAD (2011)
Reduced soil compaction	<1 million people	Low confidence	Chamen et al. (2015), Epron et al. (2016), Tullberg et al. (2018)
Biochar addition to soil	Up to 3,200 million people; but potential negative (unquantified) impacts if arable land used for feedstock production	Low confidence	Jeffery et al. (2017)
Fire management	>5.8 million people affected by wildfire; max. 0.5 million deaths per year by smoke	Medium confidence	Doerr and Santín (2016), Johnston et al. (2012), Koplitz et al. (2016)
Reduced landslides and natural hazards	>25 million people	Low confidence	Arnáez, Lana‐Renault, Lasanta, Ruiz‐Flaño, and Castroviejo (2015), Gariano and Guzzetti (2016)
Reduced pollution including acidification	Prevent 0.5–4.6 million annual premature deaths globally	Medium confidence	Anenberg et al. (2012), Shindell et al. (2012), West et al. (2013), UNEP and WMO (2011)
Management of invasive species/encroachment	No global estimates	No evidence	
Restoration and reduced conversion of coastal wetlands	up to 93–310 million people	Low confidence	Hinkel et al. (2014)
Restoration and reduced conversion of peatlands	No global estimates	No evidence	
Biodiversity conservation	Likely many millions	Low confidence	CBD (2008)
Enhanced weathering of minerals	No global estimates	No evidence	
Bioenergy and BECCS	Potentially large negative consequences from competition for arable land and water.	Low confidence	Fuss et al. (2018), Muller et al. (2017), Smith, Davis, et al. (2016)

Abbreviation: BECCS, bioenergy with carbon capture and strorage.

*Improved cropland management* is a key climate adaptation option, potentially affecting more than 25 million people, including a wide range of technological decisions by farmers. Actions toward adaptation fall into two broad overlapping areas: (a) accelerated adaptation to progressive climate change over decadal timescales, for example, integrated packages of technology, agronomy, and policy options for farmers and food systems, including changing planting dates and zones, tillage systems, crop types, and varieties; and (b) better management of agricultural risks associated with increasing climate variability and extreme events, for example, improved climate information services and safety nets (Challinor et al., 2014; Lipper et al., 2014; Lobell, 2014; Vermeulen et al., 2012). In the same way, *improved livestock management* is another technological adaptation option potentially benefiting 1–25 million people. Crop and animal diversification are considered the most promising adaptation measures (Porter et al., 2014; Rojas‐Downing, Nejadhashemi, Harrigan, & Woznicki, 2017). In grasslands and rangelands, *improved grazing land management* through regulation of stocking rates, grazing field dimensions, establishment of exclosures, and locations of drinking troughs and feeders are strategic decisions by farmers that can deliver adaptation benefits (Mekuria & Aynekulu, 2013; Porter et al., 2014; Taboada, Rubio, & Chaneton, 2011).

Around 30% of the world's rural population use trees across 46% of all agricultural landscapes (Lasco, Delfino, Catacutan, Simelton, & Wilson, 2014), meaning that up to 2.3 billion people benefit from *agroforestry*, globally (Table 8).

*Agricultural diversification* is key to achieving climatic resilience (Campbell et al., 2014; Cohn et al., 2017). Crop diversification is an important climate change adaptation option (Vermeulen et al., 2012), which can improve resilience by engendering a greater ability to suppress pest outbreaks and dampen pathogen transmission, as well as by buffering crop production from the effects of greater climate variability and extreme events (Lin, 2011).

*Reduced conversion of grassland to cropland* may lead to adaptation benefits by stabilizing soils in the face of extreme climatic events, since grasslands are more resilient than cropping systems (Lal, 2001), thereby increasing resilience, but since it would likely have a negative impact on food production/security (since croplands produce more food per unit area than grasslands), the wider adaptation impacts would likely be negative. However, there is no literature quantifying the global impact of avoidance of conversion of grassland to cropland on adaptation.

*Integrated water management* provides large co‐benefits for adaptation (Dillon & Arshad, 2016) by improving the resilience of crop production systems to future climate change (Porter et al., 2014; Table 8). Improving irrigation systems and integrated water resource management, such as enhancing urban and rural water supplies and reducing water evaporation losses (Dillon & Arshad, 2016), are significant options for enhancing climate adaptation. Many technical innovations (e.g., precision water management) can lead to beneficial adaptation outcomes by increasing water availability and the reliability of agricultural production, using different techniques of water harvesting, storage, and its judicious utilization through farm ponds, dams, and community tanks in rainfed agriculture areas. Integrated water management practices that use freshwater would be expected to have few adverse side effects in regions where water is plentiful, but large adverse side effects in regions where water is scarce (Grey & Sadoff, 2007; Liu et al., 2017; Scott et al., 2011).

*Improved and sustainable forest management* positively impacts adaptation by limiting the negative effects associated with pollution (of air and fresh water), diseases, exposure to extreme weather events and natural disasters, and poverty (e.g., Smith et al., 2014). Furthermore, sustainable forest management has a number of potential co‐benefits for adaptation, ecosystem services, biodiversity conservation, microclimatic and water regulation, soil erosion protection and coastal area protection (Locatelli, 2011).

There is high agreement that *reduced deforestation* positively affects adaptation and resilience of coupled human–natural systems and the stability of the water cycle. Based on the number of people affected by natural disasters (CRED, 2015), the number of people depending to varying degrees on forests for their livelihoods (World Bank et al., 2009), the area of managed forest and the current annual deforestation rate (Keenan et al., 2015), the estimated global potential effect for adaptation is largely positive for *forest management*, and moderately positive for *reduced deforestation* when accumulated until the end of the century (Table 8). The uncertainty of these global estimates is high.

More robust qualitative and some quantitative estimates are available at local and regional level. According to Karjalainen, Sarjala, and Raitio (2009), *reducing deforestation* and habitat alteration contribute to limiting infectious diseases such as malaria in Africa, Asia, and Latin America, thus lowering the expenses associated with healthcare treatments. Bhattacharjee and Behera (2017) found that human lives lost due to floods increase with reducing forest cover and increasing deforestation rates in India. In addition, maintaining forest cover in urban contexts reduces air pollution and therefore avoids mortality of about one person per year per city in the United States, and up to 7.6 people per year in New York City (Nowak, Hirabayashi, Bodine, & Greenfield, 2014). There is also evidence that *reduced deforestation and degradation* in mangrove plantations potentially improves soil stabilization, and attenuates the impact of tropical cyclones and typhoons along the coastal areas in South and Southeast Asia (Chow, 2018). At local scales, co‐benefits between REDD+ and adaptation of local communities can potentially be substantial (Long, 2013; Morita & Matsumoto, 2018), even if often difficult to quantify, and not explicitly acknowledged (McElwee et al., 2017).

*Forest restoration* may facilitate the adaptation and resilience of forests to climate change by enhancing connectivity between forest areas and conserving biodiversity hotspots (Dooley & Kartha, 2018; Ellison et al., 2017; Locatelli, Catterall, et al., 2015; Locatelli, Evans, Wardell, Andrade, & Vignola, 2011; Locatelli, Pavageau, Pramova, & Di Gregorio, 2015). Furthermore, forest restoration may improve ecosystem functionality and services, provide microclimatic regulation for people and crops, wood and fodder as safety nets, soil erosion protection and soil fertility enhancement for agricultural resilience, coastal area protection, water and flood regulation (Locatelli, Catterall, et al., 2015; Locatelli, Pavageau, et al., 2015).

*Afforestation and reforestation* are important climate change adaptation practices (Ellison et al., 2017; Locatelli, Catterall, et al., 2015; Locatelli, Pavageau, et al., 2015; Reyer, Guericke, & Ibisch, 2009), and can potentially help a large proportion of the global population to adapt to climate change and to associated natural disasters (Table 8). For example, trees general mitigate summer mean warming and temperature extremes (Findell et al., 2017; Sonntag, Pongratz, Reick, & Schmidt, 2016).

*Soil organic carbon increase* is promoted as an action for climate change adaptation. Since increasing soil organic matter content is a measure to address land degradation, and restoring degraded land helps to improve resilience to climate change, soil carbon increase is an important option for climate change adaptation. With around 120 thousand km^2^ land lost to degradation every year, and over 3.2 billion people negatively impacted by land degradation globally (IPBES, 2018), practices designed to increase soil organic carbon have a large potential to address adaptation needs (Table 8).

Since *soil erosion control* can prevent land degradation and desertification, it improves the resilience of agriculture to climate change and increases food production (IPBES, 2018; Lal, 1998), though the global number of people benefiting from improved resilience to climate change has not been reported in the literature. Using figures from (FAO & ITPS, 2015), IPBES (2018) estimate that land losses due to erosion are equivalent to 1.5 Mkm^2^ of land used for crop production to 2050, or 45 thousand km^2^/year (Foley et al., 2011). Control of soil erosion (water and wind) could benefit 11 Mkm^2^ of degraded land (Lal, 2014) and improve the resilience of at least some of the 3.2 billion people affected by land degradation (IPBES, 2018), suggesting positive impacts on adaptation. Management of erosion is an important climate change adaptation measure, since it reduces the vulnerability of soils to loss under climate extremes, thereby increasing resilience to climate change (Garbrecht, Nearing, Steiner, Zhang, & Nichols, 2015).

*Prevention and/or reversal of topsoil salinization* requires the combined management of groundwater, irrigation techniques, drainage, mulching, and vegetation, with all of these considered relevant for adaptation (Dagar et al., 2016; Qadir, Noble, & Chartres, 2013; UNCTAD, 2011). Taking into account the widespread diffusion of salinity problems, many people can benefit from its implementation by farmers. The relation between *compaction prevention* and/or reversion and climate adaption is less evident, and can be related to better hydrological soil functioning (Chamen et al., 2015; Epron et al., 2016; Tullberg et al., 2018).

*Biochar* has potential to benefit climate adaptation by improving the resilience of crop production systems to future climate change by increasing yield in some regions and improving water holding capacity (Sohi, 2012; Woolf, Amonette, Street‐Perrott, Lehmann, & Joseph, 2010). By increasing yield by 25% in the tropics (Jeffery et al., 2017), this could increase food production for 3.2 billion people affected by land degradation (IPBES, 2018), thereby potentially improving their resilience to climate change shocks (Table 8). The use of large areas of land to provide feedstock for biochar could adversely impact adaptation by occupying land that could be used for food production, though the impact has not been quantified globally.

In terms of *fire management*, Doerr and Santín (2016) showed that globally the average number of people killed by wildfire was 1940, and the total number of people affected was 5.8 million from 1984 to 2013. Johnston et al. (2012) showed the average mortality attributable to landscape fire smoke exposure was 339,000 deaths annually. The regions most affected were sub‐Saharan Africa (157,000) and Southeast Asia (110,000). Estimated annual mortality during La Niña was 262,000, compared with around 100,000 excess deaths across Indonesia, Malaysia, and Singapore (Table 8).

*Management of landslides and natural hazards* are usually listed among planned adaptation options in mountainous and sloped hilly areas, where uncontrolled runoff and avalanches may cause climatic disasters, affecting millions of people from both urban and rural areas. Landslide control requires both increasing plant cover and engineering practices (see Table 8).

For *pollution management*, including acidification, Anenberg et al. (2012) estimated that, for PM2.5 and ozone, respectively, fully implementing reduction measures could reduce global population‐weighted average surface concentrations by 23%–34% and 7%–17% and avoid 0.6–4.4 and 0.04–0.52 million annual premature deaths globally in 2030. UNEP and WMO (2011) considered emission control measures to reduce ozone and black carbon (BC) and estimated that 2.4 million annual premature deaths (with a range of 0.7–4.6 million) from outdoor air pollution could be avoided. West et al. (2013) estimated global GHG mitigation brings co‐benefits for air quality and would avoid 0.5 ± 0.2, 1.3 ± 0.5, and 2.2 ± 0.8 million premature deaths in 2030, 2050, and 2100, respectively.

There are no global data on the impacts of *management of invasive species*/encroachment on adaptation.

Coastal wetlands provide a natural defense against coastal flooding and storm surges by dissipating wave energy, reducing erosion, and by helping to stabilize shore sediments, so restoration may provide significant benefits for adaptation. The Ramsar Convention on Wetlands covers 1.5 Mkm^2^ across 1674 sites (Keddy, Fraser, Solomeshch, and Junk, 2009) Coastal floods currently affect 93–310 million people (in 2010) globally, and this could rise to 600 million people in 2100 with sea level rise, unless adaptation measures are taken (Hinkel et al., 2014). The proportion of the flood‐prone population that could avoid these impacts through *restoration of coastal wetlands* has not been quantified, but this sets an upper limit.

*Avoided peat impacts and peatland restoration* can help to regulate water flow and prevent downstream flooding (Munang, Andrews, Alverson, & Mebratu, 2014), but the global potential (in terms of number of people who could avoid flooding through peatland restoration) has not been quantified.

There are no global estimates of the potential of *biodiversity conservation* to improve the adaptation and resilience of local communities to climate change. Nevertheless, it is widely recognized that biodiversity, ecosystem health, and resilience improve adaptation potential (Jones, Hole, & Zavaleta, 2012). For example, mixes of tree species improves the resistance of stands to natural disturbances, such as drought, fires, and windstorms (Jactel et al., 2017), as well as stability against landslides (Kobayashi & Mori, 2017). Moreover, Protected Areas play a key role in improving adaptation (Lopoukhine et al., 2012; Watson, Dudley, Segan, & Hockings, 2014) by reducing water flow, stabilizing rock movements, creating physical barriers to coastal erosion, improving resistance to fires, and buffering storm damages (Dudley et al., 2010). Thirty‐three of 105 of the largest urban areas worldwide rely on protected areas for some, or all, of their drinking water (CBD, 2008), indicating that many millions are likely to benefit from conservation practices.

*Enhanced weathering of minerals* has been proposed as a mechanism for improving soil health and food security (Beerling et al., 2018), but there is no literature estimating the global adaptation benefits.

Large‐scale *bioenergy and BECCS* could require substantial amounts of cropland (Calvin et al., 2014; Popp et al., 2017; Smith, House, et al., 2016), forestland (Baker, Wade, Sohngen, Ohrel, & Fawcett, 2019; Favero & Mendelsohn, 2017), and water (Chaturvedi et al., 2013; Fuss et al., 2018; Hejazi et al., 2015; Popp, Dietrich, et al.,^2011^; Smith, Davis, et al., 2016; Smith, Haszeldine et al., 2016; Smith, House, et al., 2016) suggesting that bioenergy and BECCS could have adverse side effects on adaptation. In some contexts, for example, low inputs of fossil fuels and chemicals, limited irrigation, heat/drought tolerant species, and using marginal land, bioenergy can have co‐benefits for adaptation (Dasgupta et al., 2014; Noble et al., 2014). However, no studies quantify the magnitude of this effect.

#### 3.2.2 Practices based on value chain management

Decreases in pressure on land and decreases in production intensity associated with *sustainable healthy diets* or *reduced food waste* could also benefit adaptation. For example, Westhoek et al. (2014) estimate a 23% reduction of cropland in Europe through halving meat consumption. However, the size of this effect is not well quantified globally (Muller et al., 2017).

*Reducing food waste and losses* can relieve pressure on the global freshwater resource, thereby aiding adaptation. Food losses account for 215 km^3^/year of freshwater resources, which Kummu et al. (2012) report to be about 12%–15% of the global consumptive water use. Given that 35% of the global population is living under high water stress or shortage (Kummu, Ward, Moel, & Varis, 2010), reducing food waste could benefit 320–400 million people (12%–15% of the 2,681 million people affected by water stress/shortage).

While no studies report quantitative estimates of the effect of *material substitution* on adaptation, the effects are expected to be similar to reforestation and afforestation if the amount of material substitution leads to an increase in forest area. Additionally, some studies indicate that wooden buildings, if properly constructed, could reduce fire risk compared to steel, which softens when burned (Gustavsson et al., 2006; Ramage et al., 2017).

It is estimated that 500 million smallholder farmers depend on agricultural businesses in developing countries (World Bank, 2017), meaning that better promotion of value‐added products and *improved efficiency and sustainability of food processing and retailing* could potentially help up to 500 million people to adapt to climate change. However, how *sustainable sourcing* in general could help farmers and forest management is mostly unquantified. More than 1 million farmers have currently been certified through various schemes (Tayleur et al., 2017), but how much this has helped them prepare for adaptation is unknown.

*Management of supply chains* has the potential to reduce vulnerability to price volatility. Consumers in lower income countries are most affected by price volatility, with sub‐Saharan Africa and South Asia at highest risk (Fujimori et al., 2019; Regmi & Meade, 2013). However, understanding the stability of food supply is one of the weakest links in global food system research (Wheeler & von Braun, 2013) as instability is driven by a confluence of factors (Headey & Fan, 2008). Food price spikes in 2007 increased the number of people under the poverty line by between 100 million people (Ivanic & Martin, 2008) and 450 million people (Brinkman, De Pee, Sanogo, Subran, & Bloem, 2009), and caused welfare losses of 3% or more for poor households in many countries (Zezza, Carletto, Davis, Stamoulis, & Winters, 2009). Food price stabilization by China, India, and Indonesia alone in 2007–2008 led to reduced staple food prices for 2 billion people (Timmer, 2009). Spending less on food frees up money for other activities, including adaptation, but it is unknown by how much (Zezza et al., 2009; Ziervogel & Ericksen, 2010). Another example of a reduction in staple food prices occurred in Bangladesh with food stability policies saving rural households US$887 million in total (Torlesse, Kiess, & Bloem, 2003). Food supply stability through improved supply chains also potentially reduces conflicts (by avoiding food price riots, which occurred in countries with over 100 million total population in 2007–2008), and thus increases adaptation capacity (Raleigh, Choi, & Kniveton, 2015).

There are no global estimates of the contribution of *urban food systems*, in contributing to adaptation, but since the urban population in 2018 was 4.2 billion people, this sets the upper limit on those who could benefit.

*Improved energy use in food systems* in agriculture could benefit 65% (760 million people) of poor working adults who make a living through agriculture (World Bank, 2017).

#### 3.2.3 Practices based on risk management

*Reducing urban sprawl* is likely to provide adaptation co‐benefits via improved human health (Anderson, 2017; Frumkin, 2002), as sprawl contributes to reduced physical activity, worse air pollution, and exacerbation of urban heat island effects and extreme heat waves (Stone, Hess, & Frumkin, 2010). The most sprawling cities in the United States have experienced extreme heat waves more than double those of denser cities (Stone et al., 2010). Other adaption co‐benefits are less well understood; there are likely to be cost savings from managing or planning growth, as one study found 2% savings in metropolitan budgets, which could then be spent on adaptation planning (Deal & Schunk, 2004).

*Livelihood diversification* is a major adaptation strategy and form of risk management, as it can help households smooth out income fluctuations and provide a broader range of options for the future (Adger et al., 2011; Osbahr, Twyman, Neil Adger, & Thomas, 2008; Thornton & Herrero, 2014). Surveys of farmers in climate variable areas find that livelihood diversification is increasingly favored as an adaptation option (Bryan et al., 2013), although it is not always successful, since it can increase exposure to climate variability (Adger et al., 2011). There are over 570 million small farms in the world (Lowder, Skoet, & Raney, 2016); it is not clear, however, how many farmers have not yet practiced diversification and thus how many would be helped by supporting this practice (Rigg, 2006).

It has been estimated that currently more than half of smallholder farmers in the developing world still rely to some degree on *use of local seeds* (Altieri, Funes‐Monzote, & Petersen, 2012; McGuire & Sperling, 2016). Use of local seeds can potentially facilitate adaptation, as moving to use of commercial seeds can increase costs for farmers (Howard, 2015). Local seed networks and banks also protect local agrobiodiversity and landraces, which are important to facilitate adaptation, as they may be more resilient to some forms of climate change (Coomes et al., 2015; van Niekerk & Wynberg, 2017; Vasconcelos et al., 2013).

*Disaster risk management* is an essential part of adaptation strategies. For example, the Famine Early Warning System operating across three continents since the 1980s has provided millions of people across 34 countries early information on drought. Such information can assist communities and households in adapting to onset conditions (Hillbruner & Moloney, 2012). However, concerns have been raised as to how many people are actually reached by disaster risk management and early warning systems (Mahmud & Prowse, 2012), and that early warnings often do not translate into longer term livelihood adaptation (Birkmann et al., 2015).

Local *risk sharing instruments* such as rotating credit or loan groups can help facilitate adaptation. Both index and commercial crop insurance offer potential for adaptation, as insurance provides a means of buffering and transferring weather risk, saving farmers the cost of crop losses (Meze‐Hausken, Patt, & Fritz, 2009; Patt, Suarez, & Hess, 2010). However, overly subsidized insurance can undermine the market's role in pricing risks and thus depress more rapid adaptation strategies (Jaworski, 2016; Skees & Collier, 2012) and increase the riskiness of decision‐making (McLeman & Smit, 2006). For example, availability of crop insurance was observed to reduce farm‐level diversification in the United States, thereby reducing longer term adaptive capacity (Sanderson et al., 2013a, 2013b) and crop insurance‐holding soybean farmers in the United States have been less likely to adapt to extreme weather events than those not holding insurance (Annan & Schlenker, 2015). It is unclear how many people worldwide use insurance as an adaptation strategy; Platteau, De Bock, and Gelade (2017) suggest less than 30% of smallholders take out any form of insurance, but it is likely in the millions (Tables 9 and 10).

**Table 9:** Summary of adaptation effects of practices based on demand management

Practice	Potential	Confidence	Citation
Dietary change	No global estimates	No evidence	Muller et al. (2017)
Reduced post‐harvest losses	320–400 million people	Medium confidence	Kummu et al. (2012)
Reduced food waste (consumer or retailer)	No global estimates	No evidence	Muller et al. (2017)
Material substitution	No global estimates	No evidence	
Sustainable sourcing	>1 million	Low confidence	Tayleur et al. (2017)
Management of supply chains	>100 million	Medium confidence	Campbell et al. (2016), Ivanic and Martin (2008), Timmer (2009), Vermeulen et al. (2012)
Enhanced urban food systems	No global estimates	No evidence	
Improved food processing and retailing	500 million people	Low confidence	World Bank (2017)
Improved energy use in food systems	760 million	Low confidence	World Bank (2017)

**Table 10:** Summary of adaptation effects of practices based on risk management

Practice	Potential	Confidence	Citation
Management of urban sprawl	Unquantified but likely to be many millions	Low confidence	Stone et al. (2010)
Livelihood diversification	>100 million likely	Low confidence	Morton (2007), Rigg (2006)
Use of local seeds	Unquantified but likely to be many millions	Low confidence	Louwaars (2002), Santilli (2012)
Disaster risk management	>100 million	High confidence	Hillbruner and Moloney (2012)
Risk sharing instruments	Unquantified but likely to be several million	Low confidence	Platteau et al. (2017)

### 3.3 Potential of practices for addressing land degradation and desertification

#### 3.3.1 Practices based on land management

Burney et al. (2010) estimated that an additional global cropland area of 11.11–15.14 Mkm^2^ would have been needed if productivity had not increased between 1961 and 2000. Given that agricultural expansion is the main driver of land degradation and desertification, *increased food productivity* could have prevented this area from exploitation and land degradation (Table 11).

**Table 11:** Summary of effects on land degradation and desertification of practices based on land management

Practice	Potential	Confidence	Citation
Increased food productivity	11.1–15.1 Mkm^2^	Low confidence	Burney et al. (2010)
Improved cropland management	10 Mkm^2^	Low confidence	Lal (2015), Smith, House, et al. (2016), Schwilch et al. (2014)
Improved grazing land management	10 Mkm^2^	Low confidence	Smith, House, et al. (2016), Schwilch et al. (2014)
Improved livestock management	10 Mkm^2^	Low confidence	Lal (2015), Smith, House, et al. (2016), Miao et al. (2015), Squires and Karami (2005)
Agroforestry	10 Mkm^2^ (with >10% tree cover)	Medium confidence	Garrity (2012)
Agricultural diversification	1–5 Mkm^2^	Low confidence	Lambin and Meyfroidt (2011), Schwilch et al. (2014)
Reduced grassland conversion to cropland	Up to 17.4 thousand km^2^/year	Low confidence	Foley et al. (2011)
Integrated water management	10 thousand km^2^	Low confidence	Caon and Vargas (2017), UNCCD (2013)
Improved and sustainable forest management	>3 Mkm^2^	Low confidence	Caon and Vargas (2017), UNCCD (2013), Núñez et al. (2010)
Reduced deforestation and degradation	>3 Mkm^2^ by the end of the century	Low confidence	Keenan et al. (2015), Núñez et al. (2010)
Reforestation and forest restoration	>3 Mkm^2^ suitable for restoration	Medium confidence	UNCCD (2013), Wolff et al. (2018), Bastin et al. (2019)
Afforestation	up to 25.8 Mkm^2^ by the end of the century	Low confidence	Griscom et al. (2017), Kreidenweis et al. (2016), Popp et al. (2017)
Increased soil organic carbon content	Up to 11.37 Mkm^2^	Medium confidence	Lal (2001), Lal (2004)
Reduced soil erosion	Up to 11.37 Mkm^2^	Medium confidence	Lal (2001), Lal (2004)
Reduced soil salinization	0.77 Mkm^2^/year	Medium confidence	Oldeman et al. (1991)
Reduced soil compaction	10 Mkm^2^	Low confidence	FAO and ITPS (2015), Hamza and Anderson (2005)
Biochar addition to soil	No global estimates	No evidence	
Fire management	Up to 3.5–4.9 Mkm^2^/year	Medium confidence	Arora and Melton (2018), Randerson et al. (2012), Tansey et al. (2004)
Reduced landslides and natural hazards	1–5 Mkm^2^	Low confidence	FAO and ITPS (2015), Gariano and Guzzetti (2016), Djeddaoui, Chadli, and Gloaguen (2017), Noble et al. (2014)
Reduced pollution including acidification	1.03 Mkm^2^/year	Low confidence	Oldeman et al. (1991)
Management of invasive species/encroachment	No global estimates	No evidence	
Restoration and reduced conversion of coastal wetlands	0.29 Mkm^2^	Medium confidence	Griscom et al. (2017)
Restoration and reduced conversion of peatlands	0.46 Mkm^2^	Medium confidence	Griscom et al. (2017)
Biodiversity conservation	No global estimates	No evidence	
Enhanced weathering of minerals	Positive but not quantified	Low confidence	Beerling et al. (2018)
Bioenergy and BECCS	Potential impact on up to 15 Mkm^2^ with possible negative consequences; potential for positive impacts in some circumstances	Medium confidence	Clarke et al. (2014), Popp et al. (2017), Smith, Davis, et al. (2016), Smith, Haszeldine et al. (2016), Smith, House, et al. (2016)

Abbreviation: BECCS, bioenergy with carbon capture and strorage.

*Improved cropland, livestock*, and *grazing land management*, such as those outlined in the recently published Voluntary Guidelines for Sustainable Soil Management (FAO, 2017), are strategic options aiming to address land degradation and desertification. Management options include crop and animal selection, optimized stocking rates, changed tillage and/or cover crops, land use change from cropland to rangeland, increases in ground cover by vegetation, and protection against wind erosion (Bestelmeyer et al., 2015; Schwilch, Liniger, & Hurni, 2014). In many drylands, land cover is threatened by overgrazing, so management of stocking rates and grazing can help to prevent the advance of land degradation (Smith, House, et al., 2016). Considering the widespread distribution of degraded and desertified lands globally, more than 10 Mkm^2^ could benefit from improved management techniques.

*Agroforestry* can help stabilize soils to prevent land degradation and desertification, so given that there is around 10 Mkm^2^ of land with more than 10% tree cover (Garrity, 2012), agroforestry could benefit up to 10 Mkm^2^ of land.

*Agricultural diversification* usually aims to increase climate and food security resilience, for example, through “climate smart agriculture” (Table S4; Lipper et al., 2014) and may include the use of crops with manures, legumes, fodder legumes, and cover crops combined with conservation tillage systems (Schwilch et al., 2014). These practices are part of improved crop management options (see above) and aim to increase ground coverage by vegetation and control wind erosion losses. The objectives are closely related to land degradation prevention, potentially affecting 1–5 Mkm^2^.

Since shifting from grassland to the annual cultivation of crops increases erosion and soil loss, there are significant benefits for desertification control, by stabilizing soils in arid areas. Cropland expansion during 1985–2005 was 359,000 km^2^ or 17,400 km^2^/year (Foley et al., 2011). Not all of this expansion will be from grasslands or in degraded/desertified areas, but this value sets the maximum contribution of *preventing the conversion of grasslands to croplands*, a small global benefit for land degradation and desertification control (Table 11).

Most land degradation processes that are sensitive to climate change pressures (e.g., erosion, decline in soil organic matter, salinization, waterlogging, drying of wet ecosystems) benefit from *integrated water management*. Integrated water management options include management to reduce aquifer and surface water depletion, and to prevent over extraction, and provide direct co‐benefits for prevention of land degradation. Strategies such as water‐use efficiency and irrigation improve soil health through increases in soil organic matter content, thereby delivering benefits for prevention or reversal of desertification (Baumhardt, Stewart, & Sainju, 2015; Datta, De Jong, & Singh, 2000; Evans & Sadler, 2008; He, Cai, Ran, Zhao, & Jiang, 2015). Climate change will amplify existing stresses on water availability and on agricultural systems, particularly in semiarid environments. In 2011, semiarid ecosystems in the southern hemisphere contributed 51% of the global net carbon sink (Poulter et al., 2014). These results suggest that arid ecosystems could be an important global carbon sink, depending on soil water availability. Globally, water erosion is estimated to result in the loss of 23–42 MtN and 14.6–26.4 MtP annually (Caon & Vargas, 2017). Forests influence the storage and flow of water in watersheds (Eisenbies, Aust, Burger, & Adams, 2007) and are therefore important for regulating how climate change will impact landscapes.

Forests are important in helping to stabilize land and regulate water and microclimate (Locatelli, Catterall, et al., 2015; Locatelli, Pavageau, et al., 2015). Based on the extent of forests exposed to degradation (Gibbs & Salmon, 2015) and dry forests at risk of desertification (Bastin et al., 2017; Núñez et al., 2010), the estimated global potential effect for reduced land degradation and avoided desertification is large for both *forest management* and for *reduced deforestation and forest degradation* when accumulated until the end of the century (Table 11). Uncertainty in these global estimates is high. More robust estimates are available at regional levels. For example, land management may have contributed to 26% of the total land reverted from desertification in Northern China between 1981 and 2010 (Xu, Song, Li, Ding, & Wang, 2018). In Thailand, desertification risk was reduced when bare lands were converted to agriculture and forests, and from non‐forests to forests (Wijitkosum, 2016).

Forest restoration is a key option in achieving the overarching aim of reducing land degradation globally, such as through land degradation neutrality (Table S4), not only in drylands (Safriel, 2017). Indeed, it has been estimated that more than 20 Mkm^2^ are potentially suitable for forest and landscape restoration, of which 15 Mkm^2^ may be devoted to mosaic restoration (UNCCD, 2013). Excluding agricultural and urban areas, Bastin et al. (2019) suggest a global tree restoration potential of 9 Mkm^2^. Under a restoration and protection scenario, Wolff, Schrammeijer, Schulp, and Verburg (2018) simulated that there will be a global increase in net tree cover of about 4 Mkm^2^ by 2050. Moreover, under the Bonn Challenge, countries aim to restore 1.5 Mkm^2^ of deforested and degraded land by 2020, and 3.5 Mkm^2^ by 2030 (http://www.bonnchallenge.org/content/challenge). At local level, Brazil's Atlantic Restoration Pact aims to restore 0.15 Mkm^2^ of forest areas in 40 years (Melo et al., 2013). The Y Ikatu Xingu campaign in Brazil (launched in 2004) aims to contain deforestation and degradation processes by reversing forest loss on 3,000 km^2^ in the Xingu Basin (Durigan, Guerin, & Costa, 2013).

*Afforestation, reforestation*, and *forest restoration* are also used to prevent desertification. Forests tend to maintain water and soil quality by reducing runoff and trapping sediments and nutrients (Idris Medugu, Majid, Johar, & Choji, 2010; Salvati, Sabbi, Smiraglia, & Zitti, 2014), but planting of non‐native species in semiarid regions can deplete soil water resources if they have high evapotranspiration rates (Feng, Gong, Mei, & Cui, 2016). *Afforestation* and *reforestation* programs can be deployed over large areas of the Earth, so can create synergies in areas prone to desertification. Global estimates of land potentially available for afforestation are up to 25.8 Mkm^2^ by the end of the century, depending on a variety of assumptions on socioeconomic developments and climate policies (Griscom et al., 2017; Kreidenweis et al., 2016; Popp et al., 2017). The higher end of this range is achieved under the assumption of a globally uniform reward for carbon uptake in the terrestrial biosphere, and is halved by considering tropical and subtropical areas only to minimize albedo feedbacks (Kreidenweis et al., 2016). When safeguards are introduced (e.g., excluding existing cropland for food security, boreal areas, etc.), the area available declines to about 6.8 Mkm^2^ (95% confidence interval of 2.3 and 11.25 Mkm^2^), of which about 4.7 Mkm^2^ is in the tropics and 2.1 Mkm^2^ is in temperate regions (Griscom et al., 2017; Table 11). These estimates largely overlap with those for forest restoration.

*Increasing soil organic matter content* is a measure to address land degradation. With around 120 thousand km^2^ lost to degradation every year, and over 3.2 billion people negatively impacted by land degradation globally (IPBES, 2018), practices designed to increase soil organic carbon have a large potential to address land degradation (Lal, 2004). With over 2.7 billion people affected globally by desertification (IPBES, 2018), practices to increase soil organic carbon content could be applied to an estimated 11.37 Mkm^2^ of desertified land (Lal, 2001; Table 11).

*Control of soil erosion* could have large benefits for desertification control. Using figures from FAO, IPBES (2018) estimated that land losses due to erosion to 2050 are equivalent to 1.5 Mkm^2^ of land from crop production, or 45 thousand km^2^/year (Foley et al., 2011), so soil erosion control could benefit up to 1.50 Mkm^2^ of land in the coming decades. Lal (2001) estimated that desertification control (using soil erosion control as one practice) could benefit 11.37 Mkm^2^ of degraded and desertified land globally (Table 11).

Oldeman, Hakkeling, and Sombroek (1991) estimated the global extent soil affected by salinization is 0.77 Mkm^2^/year, which sets the upper limit on the area that could benefit from measures to address soil salinization. The global extent of chemical soil degradation (salinization, pollution, and acidification) is about 1.03 Mkm^2^ (Oldeman et al., 1991) giving the maximum extent of land that could benefit from the management of pollution and acidification (Table 11). In degraded arid grasslands, shrublands, and rangelands, desertification can be reversed by alleviation of soil compaction through installation of enclosures and removal of domestic livestock (Allington & Valone, 2010), but there are no global estimates of the potential benefits of doing this (Table 11).

*Biochar* could deliver benefits in efforts to address land degradation and desertification through improving water and nutrient holding capacity (Sohi, 2012; Woolf et al., 2010), and stimulating nutrient cycling and biological activity, but the global effect is not quantified.

For *fire management*, Arora and Melton (2018) estimated, using models and GFED4.1s0 data, that burned area over the 1997–2014 period was 4.834–4.855 Mkm^2^/year. Randerson, Chen, Werf, Rogers, and Morton (2012) estimated small fires increased total burned area globally by 35% from 3.45 to 4.64 Mkm^2^/year during the period 2001–2010. Tansey et al. (2004) estimated over 3.5 Mkm^2^/year of burned areas were detected in the year 2000 (Table 11).

Management of landslides and natural hazards aims to control a severe land degradation process affecting sloped and hilly areas, many of them with poor rural inhabitants (Gariano & Guzzetti, 2016), but the global potential has not been quantified.

There are no global data on the impacts of *management of invasive species*/encroachment on desertification, though the impact is presumed to be positive. There are no global studies examining the potential role of *restoration and avoided conversion of coastal wetlands* on desertification. However, since degradation of coastal wetlands is widespread, *restoration of coastal wetlands* could potentially deliver moderate benefits for addressing land degradation, with 0.29 Mkm^2^ globally considered feasible for restoration (Griscom et al., 2017; Table 11).

Large areas (0.46 Mkm^2^) of global peatlands are degraded and so, considered suitable for restoration (Griscom et al., 2017). Thus, *peatland restoration* could deliver moderate benefits for addressing land degradation (Table 11).

There are no global estimates of the effects of *biodiversity conservation* on reducing degraded lands. However, at the local scale, biodiversity conservation programs have been demonstrated to stimulate gains in forest cover over large areas over the last three decades (e.g., in China; Zhang et al., 2013). Management of wild animals can influence land degradation processes by grazing, trampling, and compacting soil surfaces, thereby altering surface temperatures and chemical reactions affecting sediment and carbon retention (Cromsigt et al., 2018).

While spreading of crushed minerals onto land as part of *enhanced mineral weathering* may provide soil/ plant nutrients in nutrient‐depleted soils (Beerling et al., 2018), there is no literature reporting on the potential global impacts of this practice in addressing land degradation or desertification.

Large‐scale production of *bioenergy and BECCS* requires significant amounts of land (Clarke et al., 2014; Popp et al., 2017; Smith, Haszeldine, et al., 2016), with as much as 15 Mkm^2^ in 2100 in 2°C scenarios (Popp et al., 2017), increasing pressures for land degradation and desertification (Table 11). However, bioenergy production can either increase (Mello et al., 2014; Robertson et al., 2017) or decrease (FAO, 2011; Lal, 2014) soil organic matter, depending on where it is produced and how it is managed. Since no global estimates of these impacts are available, they are not included in the quantification in Table 11.

#### 3.3.2 Practices based on value chain management

*Dietary change* and *waste reduction* both result in decreased cropland and pasture extent (Bajželj et al., 2014; Stehfest et al., 2009; Tilman & Clark, 2014), reducing the pressure for land degradation (Table 12). *Reduced post‐harvest losses* could spare 1.98 Mkm^2^ of cropland globally (Kummu et al., 2012) meaning that land degradation pressure could be relieved from this land area through reduction of post‐harvest losses. The effects of *material substitution* on land degradation depend on management practice; some forms of logging can lead to increased land degradation. No studies were found linking material substitution to desertification (Table 13).

**Table 12:** Summary of effects on land degradation and desertification of practices based on value chain management

Practice	Potential	Confidence	Citation
Dietary change	4–28 Mkm^2^	High confidence	Alexander et al. (2016), Bajželj et al. (2014), Stehfest et al. (2009), Tilman and Clark (2014)
Reduced post‐harvest losses	1.98 Mkm^2^	Low confidence	Kummu et al. (2012)
Reduced food waste (consumer or retailer)	7 Mkm^2^	Medium confidence	Bajželj et al. (2014)
Material substitution	No global estimates	No evidence	
Sustainable sourcing	>4 Mkm^2^	Low confidence	Auld, Gulbrandsen, and McDermott (2008)
Management of supply chains	No global estimates	No evidence	
Enhanced urban food systems	No global estimates	No evidence	
Improved food processing and retailing	No global estimates	No evidence	
Improved energy use in food systems	No global estimates	No evidence	

**Table 13:** Summary of effects on land degradation and desertification of practices based on risk management

Practice	Potential	Confidence	Citation
Management of urban sprawl	>5 thousand km^2^	Low confidence	Barbero‐Sierra, Marques, and Ruíz‐Pérez (2013), Chen, 2007, Zhang (2001)
Livelihood diversification	>0.1 Mkm^2^	Low confidence	Herrmann and Hutchinson (2005), Liu and Lan (2015)
Use of local seeds	No global estimates	No evidence	
Disaster risk management	No global estimates	No evidence	Pozzi et al. (2013)
Risk sharing instruments	Variable, but negative impact on >5 thousand km^2^ in Upper Midwest USA	Low confidence	Goodwin and Smith (2003), Wright and Wimberly (2013), Claassen et al. (2011)

There are no global estimates of the impact on land degradation of *enhanced urban food systems*, *improved food processing and retailing*, or *improved energy use in food systems*.

There is evidence that *sustainable sourcing* could reduce land degradation, as the explicit goal of sustainable certification programs is often to reduce deforestation or other unsustainable land uses. Over 4 Mkm^2^ of forests are certified for sustainable harvesting (PEFC/FSC, 2018), although it is not clear if all of these lands would be at risk of degradation without certification. Improved management of supply chains also may have an impact on reduced land degradation, although figures have not been quantified; for example, food price instability in 2007–2008 increased financial investment in cropland expansion (especially through so‐called land grabbing which has been associated with some land degradation), and thus, better *management of supply chains* might have reduced this (McMichael, 2012; McMichael & Schneider, 2011).

#### 3.3.3 Practices based on risk management

For *management of urban sprawl*, urban expansion has been identified as a major cause of soil degradation in some countries; for example, urban expansion in China has now affected 0.2 Mkm^2^, or almost one‐sixth of the cultivated land total, causing an annual grain yield loss of up to 10 Mt, or around 5%–6% of cropland production. Global cropland production losses of 8%–10% by 2030 are expected under modeled scenarios of urban expansion (Bren d'Amour et al., 2016).

Degradation can be a driver of *livelihood diversification* (Batterbury, 2001; Lestrelin & Giordano, 2007), which can be reversed if diversification involves adding nontraditional crops or trees that reduce the need for tillage (Antwi‐Agyei, Stringer, & Dougill, 2014). China's Sloping Land conversion program has had livelihood diversification benefits and may have prevented degradation on 93 thousand km^2^ of land (Liu et al., 2015). However, there is conflicting evidence from some areas that that more diverse‐income households may also increase land degradation (Palacios et al., 2013; Warren, 2002).

*Use of local seeds* may play a role in addressing land degradation as they reduce need for inputs such as chemical fertilizers or mechanical tillage (Mousseau, 2015; Reisman, 2017). Some anti‐desertification programs have also shown more success using local seed varieties (Bassoum & Ghiggi, 2010; Nunes et al., 2016). However, there are no global estimates to support this.

*Disaster Risk Management* systems can have some positive impacts on prevention and reversal of land degradation, such as the Global Drought Early Warning System (Pozzi et al., 2013). However, there are no figures for how much land area is covered by early warning systems.

*Risk sharing instruments* could have benefits for reduced degradation, but there are no global estimates. Furthermore, commercial crop insurance is likely to deliver no co‐benefits for the prevention or reversal of degradation. One study found a 1% increase in farm receipts generated from subsidized farm programs (including crop insurance and others) increased soil erosion by 0.3 t/ha (Goodwin & Smith, 2003). Wright and Wimberly (2013) found a 5,310 km^2^ decline in grasslands in the Upper Midwest of the United States during 2006–2010 due to crop conversion driven by higher prices and access to insurance. Crop insurance could have been responsible for shifting up to 0.9% of rangelands to cropland in the Upper US Midwest (Claassen et al., 2011).

### 3.4 Potential of the practices for addressing food security

#### 3.4.1 Practices based on land management

*Increased food productivity* has fed many millions of people. Erisman, Sutton, Galloway, Klimont, and Winiwarter (2008), for example, estimated that over 3 billion people worldwide could not have been fed without increased food productivity arising from N fertilization (Table 14).

**Table 14:** Summary of effects on food security of land management practices

Practice	Potential	Confidence	Citation
Increased food productivity	3,000 million people	High confidence	Erisman et al. (2008)
Improved cropland management	>1,000 million people	Low confidence	Campbell et al. (2014), Lipper et al. (2014)
Improved grazing land management	>1,000 million people	Low confidence	Herrero et al. (2016)
Improved livestock management	>1,000 million people	Low confidence	Herrero et al. (2016)
Agroforestry	Up to 1,300 million people	Low confidence	IPBES (2018)
Agricultural diversification	>1,000 million people	Low confidence	Birthal et al. (2015), Massawe et al. (2016), Waha et al. (2018)
Reduced grassland conversion to cropland	Negative impact on 16.4 million people	Low confidence	Clark and Tilman (2017), FAO (2018)
Integrated water management	>1,000 million people	High confidence	Campbell et al. (2016)
Improved and sustainable forest management	Positive impact on <100 million people	Low confidence	FAO, IFAD, and WFP (2015), Rowland et al. (2017)
Reduced deforestation and degradation	Positive impact on >100 million people	Low confidence	FAO, IFAD, and WFP (2015), Keenan et al. (2015), Rowland et al. (2017), Lawrence and Vandercar (2015)
Reforestation and forest restoration	See afforestation		
Afforestation	Estimates range from positive impact on >100 million people to a negative impact on >100 million people	Medium confidence	Boysen et al. (2017), Frank et al. (2017), Kreidenweis et al. (2016)
Increased soil organic carbon content	60–225 million people	Low confidence	Frank et al. (2017)
Reduced soil erosion	633 million people/year	Low confidence	FAO (2018), FAO et al. (2018), Lal (1998), Pradhan et al. (2013), World Bank (2018)
Reduced soil salinization	1–100 million people	Low confidence	Qadir et al. (2013)
Reduced soil compaction	1–100 million people	Low confidence	Anderson and Peters (2016)
Biochar addition to soil	Range from positive impact in the tropics from biochar addition to soil to a maximum potential negative impact on >100 million people by worst‐case conversion of 20% of global cropland	Low confidence	Jeffery et al. (2017), worse case negative impacts calculated from area values in Smith (2016)
Fire management	~62 million people	Low confidence	FAO (2015), FAO (2018), FAO et al. (2018), Pradhan et al. (2013), World Bank (2018), Forest and Climate Change Government of India Ministry of Environment and World Bank (2018)
Reduced landslides and natural hazards	1–100 million people	Low confidence	Campbell (2015)
Reduced pollution including acidification	Increase annual crop yields 30–135 Mt globally; feeds 100–450 million people	Low confidence	Shindell et al. (2012), FAO (2018), FAO et al. (2018), Pradhan et al. (2013), World Bank (2018)
Management of invasive species/encroachment	No global estimates	No evidence	
Restoration and reduced conversion of coastal wetlands	Very small negative impact but not quantified	Low confidence	
Restoration and reduced conversion of peatlands	Potential negative impact on 21–31 million people	Low confidence	Clark and Tilman (2017), FAO (2018)
Biodiversity conservation	No global estimates	No evidence	
Enhanced weathering of minerals	No global estimates	No evidence	
Bioenergy and BECCS	Worst case: potential negative impact on up to 150 million people	Medium confidence	Baldos and Hertel (2014), Fujimori et al. (2019)

Abbreviation: BECCS, bioenergy with carbon capture and strorage.

*Improved cropland management* to achieve food security aims to close yield gaps by increasing use efficiency of essential inputs such as water and nutrients. Large production increases (45%–70% for most crops) are possible from closing yield gaps to 100% of attainable yield, by optimizing fertilizer use and irrigation, although overuse of nutrients causes adverse environmental impacts (Mueller et al., 2012). This improvement could affect 1,000 million people.

*Improved grazing land management* includes grasslands, rangelands, and shrublands, and all sites on which pastoralism is practiced. In general terms, continuous grazing may cause severe damage to topsoil quality through, for example, compaction. This damage may be reversed by short grazing exclusion periods under rotational grazing systems (Drewry, 2006; Greenwood & McKenzie, 2001; Taboada et al., 2011). Due to the widespread diffusion of pastoralism, improved grassland management may potentially affect more than 1,000 million people, many of them under subsistence agricultural systems.

Meat, milk, eggs, and other animal products, including fish and other seafoods, will play an important role in achieving food security (Reynolds, Wulster‐Radcliffe, Aaron, & Davis, 2015). *Improved livestock management* with different animal types and feeds may also impact one million people (Herrero et al., 2016). Ruminants are efficient converters of grass into human edible energy and protein and grassland‐based food production can produce food with a comparable carbon footprint to mixed systems (O'Mara, 2012). However, in the future, livestock production will increasingly be affected by competition for natural resources, particularly land and water, competition between food and feed and by the need to operate in a carbon‐constrained economy (Thornton, Steeg, Notenbaert, & Herrero, 2009).

Currently, over 1.3 billion people are on degrading agricultural land (IPBES, 2018), and the combined impacts of climate change and land degradation could reduce global food production by 10% by 2050. Since *agroforestry* could help to address land degradation, up to 1.3 billion people could benefit in terms of food security through agroforestry.

*Agricultural diversification* is not always economically viable; technological, biophysical, educational, and cultural barriers may emerge that limit the adoption of more diverse farming systems. Nevertheless, diversification could benefit 1,000 million people, many of them under subsistence agricultural systems (Birthal, Roy, & Negi, 2015; Massawe, Mayes, & Cheng, 2016; Waha et al., 2018).

Cropland expansion during 1985–2005 was 17,000 km^2^/year (Foley et al., 2005). Given that cropland productivity (global average of 250 kg protein ha^−1^ year^−1^ for wheat; Clark & Tilman, 2017) is greater than that of grassland (global average of about 10 kg protein ha^−1^ year^−1^ for beef/mutton; Clark & Tilman, 2017), *prevention of conversion of grassland to cropland* would have led to a loss of about 0.4 Mt protein/year globally. Given an average protein consumption in developing countries of 25.5 kg protein/year (equivalent to 70 g person^−1^ day^−1^; FAO, 2018), this is equivalent to the protein consumption of 16.4 million people each year (Table 14).

*Integrated water management* provides direct benefits to food security by improving agricultural productivity (Godfray & Garnett, 2014; Tilman, Balzer, Hill, & Befort, 2011), thereby potentially affecting the livelihood and well‐being of >1,000 million people (Campbell et al., 2016) suffering hunger and highly vulnerable by climate change. Increasing water availability through reliable supply of water for agricultural production using different techniques of water harvesting, storage, and its judicious utilization through farm ponds, dams, and community tanks in rainfed agriculture areas (Rao, Rejani, et al., 2017; Rivera‐Ferre et al., 2016), thereby potentially affecting the livelihood and well‐being of >1,000 million people (Campbell et al., 2016) suffering hunger and highly vulnerable by climate change.

Forests play a major role in providing food to local communities (non‐timber forest products, mushrooms, fodder, fruits, berries, etc.), and diversify daily diets directly or indirectly through improving productivity, hunting, diversifying tree–cropland–livestock systems, and grazing in forests. Based on the extent of forest contributing to food supply, considering the people undernourished (FAO, IFAD, & WFP, 2015; Rowland, Ickowitz, Powell, Nasi, & Sunderland, 2017) and the annual deforestation rate (Keenan et al., 2015), the global potential to enhance food security is moderate for *improved forest management* and large for *reduced deforestation* (Table 14).

Deforestation could reduce local precipitation by 20%, severely impacting non‐irrigated agricultural lands (Lawrence & Vandercar, 2015). A 20% decrease in water availability close to tropical forests could impact 100s of millions of people. For example, if 50% of the Amazon and Congo Basins were deforested, 115 million people would be impacted given that the population of people within ~1,000 km of these basins is 578 million people, if only 20% of the population is negatively impacted. Impacts on people in other countries affected by teleconnections or exports are not included in this conservative estimate, which is also conservative since 60% of population in Congo Basin are farmers, most on unirrigated farms with large poor population centers, and 10% of people in South America work in the agriculture sector with large population centers relying on food produced close to the Amazon region. *Reduced deforestation and degradation* could therefore deliver benefits for food security for many more than 100 million people (Table 14).

The uncertainty of these global estimates is high. More robust qualitative and some quantitative estimates are available at the regional level. For example, managed natural forests, shifting cultivation, and agroforestry systems are demonstrated to be crucial to food security and nutrition for hundreds of million people in rural landscapes worldwide (Sunderland et al., 2013; Vira, Wildburger, & Mansourian, 2015). According to Erb et al. (2016), deforestation would not be needed to feed the global population by 2050, in terms of quantity and quality of food. At the local level, Cerri et al. (2018) suggested that reduced deforestation, along with integrated cropland‐livestock management, would positively affect more than 120 million people in the Cerrado, Brazil. In sub‐Saharan Africa, where population and food demand are projected to continue to rise substantially, reduced deforestation may have strong positive effects on food security (Doelman et al., 2018).

*Afforestation* and *reforestation* may negatively affect food security (Boysen, Lucht, & Gerten, 2017; Frank et al., 2017; Kreidenweis et al., 2016). It is estimated that large‐scale afforestation plans could increase food prices by 80% by 2050 (Kreidenweis et al., 2016), and more general mitigation measures in the agriculture, forestry and other land use sector could cause undernourishment in 80–300 million people (Frank et al., 2017; Table 14). For reforestation, the potential adverse side affects with food security are smaller than afforestation, because forest regrows on recently deforested areas, and its impact would be felt mainly through impeding possible expansion of agricultural areas.

On a smaller scale and when implemented sustainably, forested land also offers benefits in terms of food supply, especially when forest is established on degraded land and other land that cannot be used for agriculture. For example, food from forests represents a safety net during times of food and income insecurity (Wunder, Angelsen, & Belcher, 2014), and wild‐harvested meat and fish provide 30%–80% of protein intake from many rural communities (McIntyre, Liermann, & Revenga, 2016; Nasi, Taber, & Vliet, 2011). An example of how an afforestation/reforestation program has improved food security for >100 million people is the “Grain for Green” program in China. The results indicate that the area of land affected by heavy and severe soil erosion has decreased by 55.2% and 53.6%, respectively, while the water holding capacity was 25.2% higher in 2009 than that in 1990. Increased grain yields and agricultural productivity have been recorded following Grain for Green (Yao & Li, 2010), and the results strongly indicate a positive impact of cropland conversion on soil C stocks (which can increase fertility and soil water retention; Deng, Liu, & Shangguan, 2014). Most studies concur that the physical properties of the soil, including soil fertility, porosity, and nutrients, have improved, and soil erosion and river sedimentation have slowed down (Delang & Yuan, 2015). The increase in ecosystem quality measures, including fractional vegetation cover (0.1459% per year), leaf area index (0.0121 year^−1^), and net primary productivity (2.6958 g C m^−2^ year^−1^), and the mitigation of ecosystem services deterioration in soil water loss (−0.0841 t ha/year) and soil wind loss (−1.0071 t ha/year) in the Grain for Green region, indicated the positive ecological change in the Grain for Green region (Tang et al., 2019). Grain for Green has involved 124 million people in 1,897 counties in 25 provinces, which is a conservative estimate of those positively impacted, since it does not include all those potentially affected (including consumers; Table 14).

*Increasing soil organic matter stocks* can increase yield and improve yield stability (Lal, 2006; Pan, Smith, & Pan, 2009; Soussana et al., 2019), though this is not universally seen (Hijbeek et al., 2017). Lal (2006) concludes that crop yields can be increased by 20–70, 10–50, and 30–300 kg/ha for maize for wheat, rice, and maize, respectively, for every 1 t C/ha increase in soil organic carbon in the root zone. Increasing soil organic carbon by 1 t C/ha could increase food grain production in developing countries by 32 Mt/year (Lal, 2006). Frank et al. (2017) estimate that soil carbon sequestration could reduce calorie loss associated with agricultural mitigation measures by 65%, saving 60–225 million people from undernourishment compared to a baseline without soil carbon sequestration (Table 14).

Lal (1998) estimated the risks of global annual loss of food production due to accelerated erosion to be as high as 190 Mt/year of cereals, 6 Mt/year of soybean, 3 Mt/year of pulses, and 73 Mt/year of roots and tubers. Considering only cereals, if we assume per capita annual grain consumption in developing countries to be 300 kg/year (estimated based on data included in FAO, 2018; FAO, IFAD, UNICEF, WFP, & WHO, 2018; Pradhan et al., 2013; World Bank, 2018), the loss of 190 Mt/year of cereals (that could be prevented by *soil erosion control*) is equivalent to that consumed by 633 million people, annually (Table 14).

Although there are biophysical barriers, such as access to appropriate water sources and limited productivity of salt‐tolerant crops, *prevention/reversal of soil salinization* could benefit 1–100 million people (Qadir et al., 2013). Soil compaction affects crop yields, so *prevention of soil compaction* could benefit an estimated 1–100 million people globally (Anderson & Peters, 2016).

*Biochar*, on balance, could provide moderate benefits for food security by improving yields by 25% in the tropics, but with more limited impacts in temperate regions (Jeffery et al., 2017), or through improved water holding capacity and nutrient use efficiency (Sohi, 2012). These benefits could, however, be tempered by additional pressure on land if large quantities of biomass are required as feedstock for biochar production, thereby causing potential conflicts with food security (Smith, 2016). Smith (2016) estimated that 0.4–2.6 Mkm^2^ of land would be required for biomass feedstock to deliver 2.57 Gt CO_2_eq/year of CO_2_ removal. If biomass production occupied 2.6 Mkm^2^ of cropland, equivalent to around 20% of the global cropland area, this could potentially have a large effect on food security, although Woolf et al. (2010) argue that abandoned cropland could be used to supply biomass for biochar, thus avoiding competition with food production. Similarly, Woods et al. (2015) estimate that 5–9 Mkm^2^ of land is available for biomass production without compromising food security and biodiversity, considering marginal and degraded land and land released by pasture intensification (Table 14).

FAO (2015) calculated that damage from forest fires between 2003 and 2013 affected a total of 49 thousand km^2^ of crops with the vast majority in Latin America. Based on the world cereal yield in 2013 reported by Word Bank (2018; 3.8 t/ha), the loss of 49 thousand km^2^ of crops is equivalent to 18.6 Mt/year of cereals lost. Assuming annual grain consumption per capita to be 300 kg/year (estimated based on data included in FAO, 2018; FAO et al., 2018; Pradhan et al., 2013; World Bank, 2018), the loss of 18.6 Mt/year would remove cereal crops equivalent to that consumed by 62 million people, providing an estimate of the potential of *fire management* to contribute to food security (Table 14).

*Landslides and other natural hazards* affect 1–100 million people globally, so preventing them could provide food security benefits to this many people.

In terms of *measures to tackle pollution*, including acidification, Shindell et al. (2012) considered about 400 emission control measures to reduce ozone and BC. This strategy increases annual crop yields by 30–135 Mt due to ozone reductions in 2030 and beyond. If annual grain consumption per capita is assumed as 300 kg/year (estimated based on data included in FAO, 2018; FAO et al., 2018; Pradhan et al., 2013; World Bank, 2018), increase in annual crop yields by 30–135 Mt feeds 100–450 million people.

There are no global data on the impacts of *management of invasive species*/encroachment on food security.

Since large areas of converted coastal wetlands are used for food production (e.g., mangroves converted for aquaculture; (Naylor et al., 2000), *restoration of coastal wetlands* could potentially displace food production and damage local food supply, potentially leading to adverse impacts on food security, though these effects are likely to be very small given that a small proportion of human food comes from the oceans and other aquatic ecosystems (Pimentel, 2006). These impacts could be offset by careful management, such as the careful siting of ponds within mangroves (Naylor et al., 2000; Table 14).

Around 14%–20% (0.56–0.80 Mkm^2^) of the global 4 Mkm^2^ of peatlands are used for agriculture, mostly for meadows and pasture, meaning that if under *peatland restoration*, all of these peatlands were removed from production, 0.56–0.80 Mkm^2^ of agricultural land would be lost. Assuming livestock production on this land (since it is mostly meadow and pasture) with a mean productivity of 9.8 kg protein ha^−1^ year^−1^ (calculated from land footprint of beef/mutton in Clark & Tilman, 2017), and average protein consumption in developing countries of 25.5 kg protein/year (equivalent to 70 g person^−1^ day^−1^; FAO, 2018), this would be equivalent to 21–31 million people no longer fed from this land (Table 14).

There are no global estimates on how *biodiversity conservation* improves nutrition (i.e., number of nourished people), but biodiversity, and its conservation, is crucial for improving sustainable and diversified diets (Global Panel on Agriculture & Food Systems for Nutrition, 2016). Indirectly, the loss of pollinators (due to combined causes, including the loss of habitats and flowering species) would contribute to 1.42 million additional deaths per year from noncommunicable and malnutrition‐related diseases, and 27 million lost disability‐adjusted life‐years per year (Smith et al., 2015). However, at the same time, some options to preserve biodiversity, such as protected areas, may potentially conflict with food production by local communities (Molotoks, Kuhnert, Dawson, & Smith, 2017).

The spreading of crushed minerals on land as part of *enhanced mineral weathering* on nutrient‐depleted soils can potentially increase crop yield by replenishing plant available silicon, potassium, and other nutrients (Beerling et al., 2018), but there are no estimates of the potential magnitude of this effect for global food production.

Although Woods et al. (2015) estimate that 5–9 Mkm^2^ of land could be available for bioenergy feedstock production without compromising food security or biodiversity, competition for land between bioenergy and food crops could lead to adverse side effects for food security. Many studies indicate that *bioenergy/BECCS* could increase food prices (Calvin et al., 2014; Popp et al., 2017; Wise et al., 2009). Only three studies were found that link bioenergy to the population at risk of hunger, but they estimate an increase in this population of between 2 million and 150 million people (Table 14).

#### 3.4.2 Practices based on value chain management

*Dietary change* can free up agricultural land for additional production (Bajželj et al., 2014; Stehfest et al., 2009; Tilman & Clark, 2014) and reduce the risk of some diseases (Aleksandrowicz et al., 2016; Tilman & Clark, 2014), with large positive impacts on food security (Table 15).

**Table 15:** Summary of effects on food security of demand management options

Practice	Potential	Confidence	Citation
Dietary change	821 million people	High confidence	Aleksandrowicz et al. (2016), Tilman and Clark (2014)
Reduced post‐harvest losses	1,000 million people	Medium confidence	Kummu et al. (2012)
Reduced food waste (consumer or retailer)	700–1000 million people	Medium confidence	FAO (2018), Kummu et al. (2012)
Material substitution	No global estimates	No evidence	
Sustainable sourcing	>1 million people	Low confidence	Tayleur et al. (2017)
Management of supply chains	>1 million people	Low confidence	FAO (2018), Kummu et al. (2012)
Enhanced urban food systems	Up to 1,260 million people	Low confidence	Benis and Ferrão (2017), de Zeeuw and Drechsel (2015), Padgham, Jabbour, and Dietrich (2014), Specht et al. (2014)
Improved food processing and retailing	500 million people	Low confidence	World Bank (2017)
Improved energy use in food systems	Up to 2,500 million people	Low confidence	IEA (2014)

Kummu et al. (2012) estimate that an additional 1 billion people could be fed if food waste was halved globally. This includes both *post‐harvest losses* and *retail and consumer waste*, and measures such as improved food transport and distribution (Table 15).

While no studies quantified the effect of *material substitution* on food security, the effects are expected to be similar to reforestation and afforestation if the amount of material substitution leads to an increase in forest area.

Since 821 million people are undernourished (FAO, 2018), this sets the maximum number of those who could potentially benefit from better food access through *sustainable sourcing* or *better management of supply chains*. Currently, however, only 1 million people are estimated to benefit from sustainable sourcing (Tayleur et al., 2017). *Supply chain management* has a direct effect on food security; for example, food price spikes affect food security and health, with clearly documented effects of stunting among young children as a result of the 2007–2008 food supply crisis (Arndt, Hussain, & Østerdal, 2012; Brinkman et al., 2009; de Brauw, 2011; Darnton‐Hill & Cogill, 2010) with a 10% increase in wasting attributed to the crisis in South Asia alone (Vellakkal et al., 2015). There is conflicting evidence on the impacts of different food price stability options for supply chains, and little quantification of these (Alderman, 2010; Byerlee, Jayne, & Myers, 2006; del Ninno, Dorosh, & Subbarao, 2007; von Braun, Algieri, & Kalkuhl, 2014). Reduction in staple food prices due to price stabilization resulted in more expenditure on other foods and increased nutrition (e.g., oils, animal products), leading to a 10% reduction in malnutrition among children in one study (Torlesse et al., 2003), while protectionist policies (food price controls) and safety nets to reduce price instability resulted in a 20% decrease in risk of malnutrition in another (Nandy, Daoud, & Gordon, 2016). Models using policies for food aid and domestic food reserves to achieve food supply and price stability showed the highest effectiveness of all options in achieving climate mitigation and food security goals (e.g., more effective than carbon taxes) as they did not exacerbate food insecurity and did not reduce ambitions for achieving temperature goals (Fujimori et al., 2019).

For *urban food systems*, increased food production in cities combined with governance systems for distribution and access can improve food security, with a potential to produce 30% of food consumed in cities. The urban population in 2018 was 4.2 billion people, so 30% represents 1,230 million people who could benefit in terms of food security from improved urban food systems (Table 15).

It is estimated that 500 million smallholder farmers depend on agricultural businesses in developing countries (World Bank, 2017), which set the maximum number of people who could benefit from *improved food processing and retailing*.

Up to 2,500 million people could benefit from *improved energy efficiency* in agriculture, based on the estimated number of people worldwide lacking access to clean energy and instead relying on biomass fuels for their household energy needs (IEA, 2014).

#### 3.4.3 Practices based on risk management

Unregulated *urban sprawl* can affect food security; highly productive soils have experienced the highest rate of conversion of any soil type in the United States (Nizeyimana et al., 2001). Specific types of agriculture are often practiced in urban‐influenced fringes, such as fruits, vegetables, and poultry and eggs, the loss of which can have an impact on the types of nutritious foods available in urban areas (Francis et al., 2012). China experienced a loss of 30 Mt of grain production from 1998 to 2003 attributed to urbanization (Chen, 2007). However, overall global quantification has not been attempted (Table 16).

**Table 16:** Summary of effects on food security of risk management options

Practice	Potential	Confidence	Citation
Management of urban sprawl	>1 million likely	Low confidence	Bren d'Amour et al. (2016), Chen (2017)
Livelihood diversification	>100 million	Low confidence	Morton (2007)
Use of local seeds	>100 million	Low confidence	Altieri et al. (2012)
Disaster risk management	> 100 million	Medium confidence	Genesio et al. (2011), Hillbruner and Moloney (2012)
Risk sharing instruments	>1 million likely	Low confidence	Claassen et al. (2011), Goodwin et al. (2004)

*Livelihood diversification* is associated with increased welfare and incomes and decreased levels of poverty in several country studies (Arslan et al., 2018; Asfaw, Pallante, & Palma, 2018). These are likely to have large food security benefits (Barrett, Reardon, & Webb, 2001; Niehof, 2004), but there is little global quantification.

*Use of local seeds* can provide considerable benefits for food security because of the increased ability of farmers to revive and strengthen local food systems (McMichael & Schneider, 2011); studies have reported more diverse and healthy food in areas with strong food sovereignty networks (Bisht et al., 2018; Coomes et al., 2015). Women in particular may benefit from seed banks for low value, but nutritious crops (Patnaik, Jongerden, & Ruivenkamp, 2017). However, there may be lower productivity yields from local and unimproved seeds, so the overall impact on food security is ambiguous (McGuire & Sperling, 2016).

*Disaster risk management* approaches can have important impacts on reducing food insecurity, and current systems for drought warning and other storms currently reach over 100 million people. When these early warning systems help farmers harvest crops in advance of impending weather events, or make agricultural decisions to prepare for adverse events, they are likely to have positive impacts on food security (Fakhruddin, Kawasaki, & Babel, 2015). Famine early warning systems have been successful in Sahelian Africa to alert authorities of impending food shortages so that food acquisition and transportation from outside the region can begin, potentially helping millions of people (Genesio et al., 2011; Hillbruner & Moloney, 2012).

*Risk sharing instruments* are often aimed at sharing food supplies, and thus are likely to have important, but unquantified, benefits for food security. Crop insurance in particular has generally led to (modest) expansions in cultivated land area and increased food production (Claassen et al., 2011; Goodwin, Vandeveer, & Deal, 2004).

### 3.5 Summary of the potentials of practices across mitigation, adaptation, desertification, land degradation, and food security

Table 17 provides a summary of the potentials of practices across mitigation, adaptation, desertification, land degradation, and food security, using the thresholds given in Table 4.

**Table 17:** Summary of the global potentials of practices across mitigation, adaptation, desertification, land degradation, and food security, using the thresholds given in Table 4

Category	Practice	Mitigation	Adaptation	Land degradation and desertification	Food security
Agriculture	Increased food productivity	l	m	m	h
	Agroforestry	m	m	m	l
	Improved cropland management	m	l	l	l
	Improved livestock management	m	l	l	l
	Agricultural diversification	l	l	m	l
	Improved grazing land management	m	l	l	l
	Integrated water management	l	l	l	l
	Reduced grassland conversion to cropland	l	ND	l	l
Forestry	Forest management	m	l	l	l
	Reduced deforestation and degradation	h	l	l	l
	Reforestation and forest restoration	m	m	m	m
	Afforestation	m	m	l	m
Soils	Increased soil organic carbon content	H	l	m	l
	Reduced soil erosion	L	l	m	l
	Reduced soil salinization	ND	l	l	l
	Reduced soil compaction	ND	1	l	l
	Biochar addition to soil	M	ND	l	l
Other ecosystems	Fire management	M	m	m	l
	Reduced landslides and natural hazards	L	l	l	l
	Reduced pollution including acidification	M	m	l	l
	Restoration and reduced conversion of coastal wetlands	M	l	m	l
	Biodiversity conservation	L	l	ND	ND
	Restoration and reduced conversion of peatlands	M	ND	m	l
	Management of invasive species/encroachment	ND	ND	ND	ND
CDR	Enhanced weathering of minerals	M	ND	l	ND
	Bioenergy and BECCS	H	l	l	l
Demand	Reduced post‐harvest losses	H	m	m	m
	Dietary change	H	ND	h	h
	Reduced food waste (consumer or retailer)	H	ND	m	m
	Material substitution	M	ND	ND	ND
Supply	Sustainable sourcing	ND	l	l	l
	Improved food processing and retailing	l	l	ND	l
	Improved energy use in food systems	l	l	ND	l
	Management of supply chains	ND	m	ND	l
	Enhanced urban food systems	ND	ND	ND	l
Risk	Livelihood diversification	ND	l	l	l
	Use of local seeds	ND	l	ND	l
	Disaster risk management	ND	h	ND	m
	Management of urban sprawl	ND	l	m	l
	Risk sharing instruments	l	l	l	l

Note

Cell colors correspond to the large, moderate, and small categories shown in Table 4. Dark blue = large positive; mid‐blue = moderate positive; light blue = small positive; no color = no effect; light red = small negative; mid‐red = moderate negative; dark red = large negative; green = variable. Hatching for the cell showing land degradation and desertification impacts of Bioenergy and BECCS indicates uncertainty in the magnitude of the negative impact; while large‐scale production of bioenergy could require up to 15 Mkm^2^ in 2100 in 2°C scenarios, it is not known how much of this land would be degraded/desertified by such land use change. Letters in cells: l, m, and h correspond to low, medium, and high confidence that the largest estimated potential is within the indicated magnitude category. ND = no data on global impact (even though regional data may exist).

Abbreviation: CDR, carbon dioxide removal; BECCS, bioenergy with carbon capture and strorage.

## 4 DISCUSSION

Understanding the potential of practices to address the land challenges is extremely important in supporting ongoing, near‐term, future policy‐making (e.g., Paris Agreement) and to attempt to bridge the gap between science, policy makers, and the general public. Moreover, the main findings are obtained by an extended literature review, which makes the study comprehensive (40 options across four land challenges) and as robust as possible (thousands of items of information). Indeed, such a wide‐ranging and inclusive assessment has not previously been conducted. The main findings, limitations, and conclusions are presented below.

### 4.1 Co‐delivery across the land challenges

Nine options deliver medium to large benefits for all four land challenges; *increased food productivity, improved cropland management, improved grazing land management, improved livestock management, agroforestry, improved forest management, increased soil organic carbon content, fire management*, and *reduced post‐harvest losses*. A further two options, *dietary change* and *reduced food waste*, have no global estimates for adaptation but have medium to large benefits for all other land challenges.

Five options have large mitigation potential (>3 Gt CO_2_eq/year) without adverse impacts on the other land challenges; *increased food productivity, reduced deforestation and degradation, increased soil organic carbon content, fire management* and *reduced post‐harvest losses*. Two further options with large mitigation potential, *dietary change* and *reduced food waste*, have no global estimates for adaptation, but show no negative impacts across the other land challenges. Five options, *improved cropland management, improved grazing land managements, agroforestry, integrated water management*, and *forest management*, have moderate mitigation potential, with no adverse impacts on the other land challenges.

Sixteen practices have large adaptation potential (>25 million people benefit), without adverse side effects on other land challenges; *increased food productivity, improved cropland management, agroforestry, agricultural diversification, improved forest management, increased soil organic carbon content, reduced landslides and natural hazards, restoration and reduced conversion of coastal wetlands, reduced post‐harvest losses, sustainable sourcing, management of supply chains, improved food processing and retailing, improved energy use in food systems, livelihood diversification, use of local seeds,* and *disaster risk management*.

Thirty‐three of the 40 practices can be applied without competing for available land. However, seven options result in competition for land. A large number of practices do not require dedicated land, including several land management options, all value chain options, and all risk management options. Four options could potentially greatly increase competition for land if applied at scales consistent with GHG removals of >3 Gt CO_2_eq/year; *afforestation, reforestation, and* land used to provide feedstock for *bioenergy/BECCS* and *biochar*. Three further options, *reduced grassland conversion to croplands, restoration and reduced conversion of peatlands*, and *restoration and reduced conversion of coastal wetlands*, have smaller or variable impacts on the competition for land.

All options are scale dependent. The potential negative impacts of *afforestation*, *reforestation*, and land used to provide feedstock for *bioenergy/BECCS* or *biochar* when applied at scales consistent with GHG removals of >3 Gt CO_2_eq/year could be at least partially ameliorated if applied on a smaller land area, or if integrated into sustainably managed landscapes. For example, the climate change mitigation potential for *bioenergy and BECCS* is large (up to 11 Gt CO_2_eq/year), but the effects of bioenergy production on land degradation, food insecurity, water scarcity, GHG emissions, and other environmental goals are scale and context specific. These effects depend on the scale of deployment, previous land use, land use producing biomass feedstock, initial carbon stocks, climatic region, and management regime. Large areas of monoculture bioenergy crops that displace other land uses can result in land competition, with adverse effects on food production, food consumption, and thus food security, as well as adverse effects for land degradation, biodiversity, and water scarcity. Integration of bioenergy into sustainably managed agricultural landscapes, however, can ameliorate these adverse impacts and can deliver co‐benefits (e.g., Rowe et al., 2011).

Some practices are more effective when applied together. For example, *dietary change* and *waste reduction* expand the potential to apply other options by freeing‐up as much as 25 Mkm^2^ of land (4–25 Mkm^2^ for *dietary change*; Alexander, Brown, Arneth, Finnigan, & Rounsevell, 2016; Bajželj et al., 2014; Stehfest et al., 2009; Tilman & Clark, 2014 and 7 Mkm^2^ for *reduced food waste*; Bajželj et al., 2014).

Most agricultural land management practices (except for *reduced grassland conversion to cropland*, which potentially adversely affects food security), deliver benefits across the four land challenges. Among the forest land management options, *afforestation* and *reforestation* have the potential to deliver large co‐benefits across all land challenges except potentially for food security, where the evidence is mixed. Some studies suggest possible adverse impacts of *afforestation/reforestation* on food security due to adverse impacts on food prices (Kreidenweis et al., 2016), while others suggest that food productivity can be increased by reducing soil erosion and increasing agricultural productivity (Yao & Li, 2010). Among the soil‐based practices, some global data are missing, but none except *biochar* (if large areas are dedicated to feedstock production) shows any potential for negative impacts. Potential negative impacts could arise from additional pressure on land if large quantities of biomass feedstock are required for biochar production (Smith, 2016), through land competition can be minimized by sustainable location and management (Woolf et al., 2010), and biochar addition to soils can improve productivity (Jeffery et al., 2017). Where global data exist, most practices in other/all ecosystems deliver benefits except for a potential moderate negative impact on food security by *restoring peatlands* currently used for agriculture. Of the two practices specifically targeted at CDR, there are missing data for *enhanced weathering of minerals* for three of the land challenges, but large‐scale *bioenergy and BECCS* show a potential large benefit for mitigation, but small to large adverse impacts on the other three land challenges, mainly driven by increased pressure on land due to feedstock demand, though again, this could be managed by sustainable location and management of the land used for feedstock production (Woods et al., 2015).

While data allow the impact of *material substitution* to be assessed only for mitigation, the three other demand‐side practices: *dietary change, reduced post‐harvest losses*, and *reduced food waste provide* large or moderate benefits across all land challenges for which data exist. Data are lacking to assess the impact of the supply‐side practices on more than three of the land challenges, but there are large to moderate benefits for all those for which data are available. Data are not available to assess the impact of risk management‐based practices on all of the land challenges, but there are small to large benefits for all of those for which data are available.

### 4.2 Study limitations and data/knowledge gaps

The analysis presented here is based on an aggregation of information from studies with a wide variety of assumptions about how response options are implemented and the contexts in which they occur. Response options implemented differently at local to global scales could lead to different outcomes. The potential magnitude of impacts of each practice is assessed using values from the literature, many of which may consider potentials in isolation of other practices. While some practices may be compatible with others, it is not possible to add the potentials together, since many are known not to be additive. Furthermore, a number of practices are mutually exclusive since they cannot be practiced on the same land, for example, afforestation cannot be practiced on the same land as cropland management. In addition, the potentials of practices quoted in literature overlap between options. For example, a component of the potential of cropland management for mitigation or adaptation may arise from soil carbon sequestration, for which there are separate estimates of potential. As a result of these issues, the potentials for each practice cannot be simply summed to get a total global potential for any of the land challenges. Assessing the combined potential requires that the practices be considering in the same framework that conserves land, excludes mutually exclusive practices on the same land area, and considers nonoverlapping practices, so cannot be done with a purely literature‐based approach.

Assessing the magnitude at global scale means that many important, context‐specific interactions, for example, by location, ecosystem type, administrative unit, cannot be accounted for, and that important regional data have not been condoered. In terms of knowledge gaps, most of the practices for which information was available have medium to high positive potential for addressing land challenges (see Table 17). However, many of the estimates have low to medium confidence and many options have no data, showing that there are considerable knowledge gaps. Knowledge of the impacts of some practice–land challenge relationships is more robust and well established in the scientific literature or other information sources (statistics, inventory data) than others (e.g., high confidence: “h” in Table 17), such as increased food productivity with food security, and reduced deforestation and forest degradation with mitigation).

The low to medium confidence may also derive from some flexibility related to the criteria used to define magnitude of impact of each practice (see Table 4). For example, magnitude criteria needed to be defined to be comparable across options from different sectors (agriculture, forestry, soil), but in defining them in this way, the interpretation of the effects of each contribution to specific land challenges may be oversimplified, (see e.g., “low confidence” for forest management and reduced deforestation and forest degradation for all land challenges except for mitigation). Furthermore, the magnitude of contribution (low, medium, high) and trend (positive, negative) may have been affected by the selected criteria (see Table 4; e.g., relevant information not found for missing thresholds).

Many practices are known to be important for at least one land challenge by lack global estimates of potential across the other land challenges, even if an impact has been demonstrated at regional level (hence the large number of “no data on global impact” cells in Table 17). This particularly affects the supply chain and risk management practices but also affects some land management practices. For example, there are no global estimates of the potential for *management of invasive species/encroachment* for any of the land challenges, despite its acknowledged benefits for preventing land degradation and desertification locally. We have retained it in the list of practices to acknowledge its potential importance, and to highlight the knowledge gap of its impact at global scale.

### 4.3 Conclusions

Most mitigation practices can be applied without competing for available land and have the potential to provide multiple co‐benefits. A further set of practices have the potential to reduce demand for land conversion, thereby enhancing the potential for other practices to deliver across climate change mitigation and adaptation, combatting land degradation and desertification, and enhancing food security. Many practices contribute positively to sustainable development and other societal goals (McElwee et al., 2019).

A number of land management options, such as improved *cropland management*, *improved forest management*, and *increased soil organic carbon content*, do not require land use change and do not create demand for more land conversion. Furthermore, a number of practices such as *increased food productivity, dietary change*, and *reduced food loss and waste* can reduce demand for land conversion, thereby potentially freeing‐up land and creating opportunities for enhanced implementation of other practices. Portfolios of different practices that reduce competition for land are possible and are applicable across a range of scales.

A wide range of adaptation and mitigation responses, for example, preserving natural ecosystems such as peatland, coastal lands and forests, reducing competition for land, fire management, soil management, and most risk management options have the potential to make positive contributions to sustainable development, ecosystem services, and other societal goals (McElwee et al., 2019).

Most of the land management‐based practices that do not increase competition for land, and almost all options based on value chain management and risk management, can contribute to eradicating poverty and eliminating hunger, while promoting good health and well‐being, clean water and sanitation, climate action, and life on land (McElwee et al., 2019). Land management‐based options that require land use change can adversely affect efforts to eradicate poverty and eliminate hunger (Molotoks et al., 2018).

Although most practices can be applied without competing for available land, some, such as land to provide feedstock for *bioenergy/BECCS* (and under some circumstances, large‐scale *afforestation*), could potentially increase demand for land conversion. If applied at scales necessary to remove CO_2_ from the atmosphere at the scales of several Gt CO_2_eq/year, this increased demand for land could lead to adverse side effects for adaptation, food security, and potentially on land degradation and desertification, so safeguards are required to ensure that expansion of energy crops does not impact natural systems and food security. If applied on a limited share of total land and integrated into sustainably managed landscapes, there will be fewer adverse side effects and some positive co‐benefits could be realized.

Reduced grassland conversion to croplands, restoration and reduced conversion of peatlands, and restoration and reduced conversion of coastal wetlands affect smaller land areas globally, so the impacts of these options are smaller globally, but could be locally significant.

Further scientific efforts are thus needed to provide policy with robust, comprehensive, and transparent approaches, models, and tools for land use forecasting, incorporating multiple side effects, that is, biophysical, economic, and social. While policies and respective support from the scientific community remain sectoral, cross‐linkages between sustainable land management and human well‐being may be missed.
