# Supplementary material for: Which practices co‐deliver food security, climate change mitigation and adaptation, and combat land degradation and desertification?
Source: Glob Chang Biol. 2019 Dec 14;26(3):1532–75. doi: 10.1111/gcb.14878 (PMC7079138; doi:10.1111/gcb.14878)
Supplement: Supplementary file 1 [file GCB-26-1532-s001.docx]

**Supplementary Online Material for “Which practices co-deliver food security, climate change mitigation and adaptation, and combat land-degradation and desertification?”**

Pete Smith^1^, Katherine Calvin^2^, Johnson Nkem^3^, Donovan Campbell^4^, Francesco Cherubini^5^, Giacomo Grassi^6^, Vladimir Korotkov^7^, Anh Le Hoang^8^, Shuaib Lwasa^9^, Pamela McElwee^10^, Ephraim Nkonya^11^, Nobuko Saigusa^12^, Jean-Francois Soussana^13^, Miguel Angel Taboada^14^, Frances Manning^1^, Dorothy Nampanzira^15^, Cristina Arias-Navarro^13^, Matteo Vizzarri^6^, Jo House^16^, Stephanie Roe^17,18^, Annette Cowie^19^, Mark Rounsevell^20,21^ & Almut Arneth^20^

Table S1 Context, caveats and supporting evidence for land management-based practices considered in this study.

| Practice | Context and caveats | Supporting evidence |
| --- | --- | --- |
| Increased food productivity | Many practices to increase food production, particularly those predicated on very large inputs of agro-chemicals, have a wide range of negative externalities leading to the proposal of sustainable intensification as a mechanism to deliver future increases in productivity that avoid these adverse outcomes. Intensification through additional input of N fertiliser, for example, would result in negative impacts on climate, soil, water and air pollution. Similarly, if implemented in a way that over-exploits the land significant negative impacts would occur, but if achieved through sustainable intensification, and used to spare land, it could reduce the pressure on land. | Burney et al. 2010; Foley et al. 2011; Garnett et al. 2013; Godfray et al. 2010; Lal 2016; Lamb et al. 2016; Lobell et al 2008.; Shcherbak et al. 2014; Smith et al. 2013; Tilman et al. 2014; Scholes et al. 2018; Balmford et al. 2018 |
| Improved cropland management | Improved cropland management can reduce greenhouse gas emissions and create soil carbon sinks, though if poorly implemented, it could increase N_2_O and CH_4_ emissions from N fertilisers, crop residues and organic amendments. It can improve resilience of food crop production systems to climate change and can be used to tackle desertification and land degradation by improving sustainable land management. It can also contribute to food security by closing crop yield gaps to increase food productivity. | Bryan et al. 2009; Chen et al. 2010; Labrière et al. 2015; Lal 2011; Poeplau and Don 2015; Porter et al. 2014; Smith et al. 2014; Smith 2008; Tilman et al. 2011 |
| Improved grazing land management | Improved grazing land management can increase soil carbon sinks, reduce greenhouse gas emissions, improve the resilience of grazing lands to future climate change, help reduce desertification and land degradation by optimising stocking density and reducing overgrazing, and can enhance food security through improved productivity. | Archer et al. 2011; Briske et al. 2015; Conant et al. 2017; Herrero et al. 2016; Porter et al. 2014; Schwilch et al. 2014; Smith et al. 2014; Tighe et al. 2012 |
| Improved livestock management | Improved livestock management can reduce greenhouse gas emissions, particularly from enteric methane and manure management. It can improve the resilience of livestock production systems to climate change by breeding better adapted livestock. It can help with desertification and land degradation, e.g. through use of more efficient and adapted breeds to allow reduced stocking densities. Improved livestock sector productivity can also increase food production. | Archer et al. 2011; Herrero et al. 2016; Miao et al. 2015; Porter et al. 2014; Rojas-Downing et al. 2017; Smith et al. 2008, 2014; Squires et al. 2005; Tighe et al. 2012 |
| Agroforestry | Agroforestry sequesters carbon in vegetation and soils. The use of leguminous trees can enhance biological N fixation and resilience to climate change. Soil improvement and the provision of perennial vegetation can help to address desertification and land degradation. Agroforestry can increase agricultural productivity, with benefits for food security. Additionally, agroforestry can enable payments to farmers for ecosystem services and reduce vulnerability to climate shocks. | Antwi-Agyei et al. 2014; Mbow et al. 2014; Mutuo et al. 2005; Rosenstock et al. 2014; Sain et al. 2017; Sida et al. 2018; Vignola et al. 2015; Yirdaw et al. 2017 Benjamin et. al. 2018; Guo et al. 2018; Herder et al. 2017; Mosquera-Losada et al. 2018; Nair et al. 2014; Ram et al. 2017; Santiago-Freijanes et. al. 2018; |
| Agricultural diversification | Agricultural diversification is targeted at adaptation but could also deliver a small carbon sink, depending on how it is implemented. It could reduce pressure on land, benefiting desertification, land degradation, food security and household income. However, the potential to achieve household food security is influenced by the market orientation of a household, livestock ownership, non-agricultural employment opportunities, and available land resources. | Birthal et al. 2015; Campbell et al. 2014; Cohn et al. 2017; Lambin and Meyfroidt 2011; Lipper et al. 2014; Massawe et al. 2016; Pellegrini and Tasciotti 2014; Waha et al. 2018 |
| Reduced grassland conversion to cropland | Stabilising soils by retaining grass cover also improves resilience, benefiting adaptation, desertification and land degradation. Since conversion of grassland to cropland usually occurs to remedy food security needs, food security could be adversely affected, since more land is required to produce human food from livestock products on grassland than from crops on cropland. | Clark and Tilman 2017; Lal 2001; de Ruiter et al. 2017; Poore & Nemecek, 2018 |
| Integrated water management | These practices can reduce aquifer and surface water depletion, and prevent over extraction, and the management of climate risks. Many technical innovations, e.g., precision water management, can have benefits for both adaptation and mitigation, although trade-offs are possible. Maintaining the same level of yield through use of site-specific water management-based approach could have benefits for both food security and mitigation. | Brindha and Pavelic 2016; Jat et al. 2016; Jiang 2015; Keesstra et al. 2018; Liu et al. 2017; Nejad 2013; Rao et al. 2017; Shaw et al. 2014; Sapkota et al. 2017; Scott et al. 2011; Waldron et al. 2017 |
| Improved and sustainable forest management | Sustainable forest management can enhance the carbon stock in biomass, dead organic matter, and soil – while providing wood-based products to reduce emissions in other sectors through material and energy substitution. A trade-off exists between different management strategies: higher harvest decreases the carbon in the forest biomass in the short term but increases the carbon in wood products and the potential for substitution effects. Through close-to-nature silvicultural techniques, sustainable forest management can potentially offer many co-benefits in terms of climate change mitigation, adaptation, biodiversity conservation, microclimatic regulation, soil erosion protection, coastal area protection and water and flood regulation. Forest management strategies aimed at increasing the biomass stock levels may have adverse side-effects, such as decreasing the stand-level structural complexity, biodiversity and resilience to natural disasters. Forest management also affects albedo and evapotranspiration. | D’Amato et al. 2011; Dooley and Kartha 2018; Ellison et al. 2017; Erb et al. 2017; Grassi et al. 2018; Griscom et al. 2017; Jantz et al. 2014; Kurz et al. 2016; Locatelli 2011; Luyssaert et al. 2018; Nabuurs et al. 2017; Naudts et al. 2016; Putz et al. 2012; Seidl et al. 2014; Smith et al. 2014; Smyth et al. 2014; Stanturf et al. 2015; Forest Europe 2016 Pingoud et al. 2018 |
| Reduced deforestation and degradation | Reducing deforestation and degradation is a major strategy to reduce global GHG emissions. The combination of reduced GHG emissions and biophysical effects results in a large climate mitigation effect, with benefits also at local level. Reduced deforestation preserves biodiversity and ecosystem services more efficiently and at lower costs than afforestation/reforestation. Efforts to reduce deforestation and forest degradation may have potential adverse side-effects, for example, reducing availability of land for farming, restricting the rights and access of local people to forest resources (e.g. firewood), or increasing the dependence of local people to insecure external funding. | Alkama and Cescatti 2016; Baccini et al. 2017; Barlow et al. 2016; Bayrak et al. 2016; Caplow et al. 2011; Curtis et al. 2018; Dooley and Kartha 2018; Griscom et al. 2017; Hansen et al. 2013; Hosonuma et al. 2012; Houghton et al. 2015; Lewis et al. 2015; Pelletier et al. 2016; Rey Benayas et al. 2009 |
| Reforestation and forest restoration | Reforestation is similar to afforestation with respect to the co-benefits and adverse side-effects among climate change mitigation, adaptation, desertification, land degradation and food security (see row on Afforestation below). Forest restoration can increase terrestrial carbon stocks in deforested or degraded forest landscapes and can offer many co-benefits in terms of increased resilience of forests to climate change, enhanced connectivity between forest areas and conservation of biodiversity hotspots. Forest restoration may threaten livelihoods and local access to land if subsistence agriculture is targeted. | Dooley and Kartha 2018; Ellison et al. 2017; Locatelli 2011; Locatelli et al. 2015; Smith et al. 2014; Stanturf et al. 2015 |
| Afforestation | Afforestation increases terrestrial carbon stocks but can also change the physical properties of land surfaces, such as surface albedo and evapotranspiration with implications for local and global climate. In the tropics, enhanced evapotranspiration cools surface temperatures, reinforcing the climate benefits of CO_2_ sequestration in trees. At high latitudes and in areas affected by seasonal snow cover, the decrease in surface albedo after afforestation becomes dominant and causes an annual average warming that counteracts carbon benefits. Net biophysical effects on regional climate from afforestation is seasonal and can reduce the frequency of climate extremes, such as heat waves, improving adaptation to climate change and reducing the vulnerability of people and ecosystems. Afforestation helps to address land degradation and desertification, as forests tend to maintain water quality by reducing runoff, trapping sediments and nutrients, and improving groundwater recharge. However, food security could be hampered since an increase in global forest area can increase food prices through land competition. Other adverse side-effects occur when afforestation is based on non-native species, especially with the risks related to the spread of exotic fast-growing tree species. For example, exotic species can upset the balance of evapotranspiration regimes, with negative impacts on water availability, particularly in dry regions. | Alkama and Cescatti 2016; Arora and Montenegro 2011; Bonan 2008; Boysen et al. 2017; Brundu and Richardson 2016; Cherubini et al. 2017; Ciais et al. 2013; Ellison et al. 2017; Findell et al. 2017; Idris Medugu et al. 2010; Kongsager et al. 2016; Kreidenweis et al. 2016; Lejeune et al 2018.; Li et al. 2015; Locatelli et al. 2015; Perugini et al. 2017; Salvati et al. 2014; Smith et al. 2013, 2014; Trabucco et al. 2008; |
| Increased soil organic carbon content | Increasing soil carbon stocks removes CO_2_ from the atmosphere and increases the water holding capacity of the soil thereby conferring resilience to climate change and enhancing adaptation capacity. It is a key strategy for addressing both desertification and land degradation. There is some evidence that crop yields and yield stability increase by increased organic matter content, though some studies show equivocal impacts. Some practices to increase soil organic matter stocks vary in their efficacy. For example, the impact of no till farming and conservation agriculture on soil carbon stocks is often positive, but can be neutral or even negative, depending on the amount of crop residues returned to the soil. If soil organic carbon stocks were increased by increasing fertiliser inputs to increase productivity, emissions of nitrous oxide from fertiliser use could offset any climate benefits arising from carbon sinks. Similarly, if any yield penalty is incurred from practices aimed at increasing soil organic carbon stocks (e.g. through extensification), emissions could be increased through indirect land use change, and there could also be adverse side-effects on food security. | Bestelmeyer and Briske 2012; Cheesman et al. 2016; Frank et al. 2017; Gao et al. 2018; Keesstra et al 2016.; Lal 2016, 2006; Lambin and Meyfroidt 2011; de Moraes Sá et al. 2017; Palm et al. 2014; Pan et al. 2009; Paustian et al. 2016; Powlson et al. 2014, 2016, Smith et al. 2013, 2016a, 2014; Soussana et al. 2019; Steinbach et al 2006.; VandenBygaart 2016; Hijbeek et al., 2017; Schjønning et al., 2018; |
| Reduced soil erosion | The fate of eroded soil carbon is uncertain, with some studies indicating a net source of CO_2_ to the atmosphere and others suggesting a net sink. Reduced soil erosion has benefits for adaptation as it reduces vulnerability of soils to loss under climate extremes, increasing resilience to climate change. Some management practices implemented to control erosion, such as increasing ground cover, can reduce the vulnerability of soils to degradation / landslides, and prevention of soil erosion is a key measure used to tackle desertification. Because it protects the capacity of land to produce food, it also contributes positively to food security. | Chen 2017; Derpsch et al. 2010; FAO and ITPS 2015; FAO 2015; Garbrecht et al. 2015; Jacinthe and Lal 2001; de Moraes Sá et al. 2017; Poeplau and Don 2015; Smith et al. 2001; Stallard 1998; Lal and Moldenhauer 1987; Van Oost et al. 2007; Lugato et al. 2016; Smith et al. 2005; Lal 2001a |
| Reduced soil salinisation | Techniques to prevent and reverse soil salinisation may have small benefits for mitigation by enhancing carbon sinks. These techniques may benefit adaptation and food security by maintaining existing crop systems and closing yield gaps for rainfed crops. These techniques are central to reducing desertification and land degradation, since soil salinisation is a primary driver of both. | Baumhardt et al. 2015; Dagar et al. 2016; Datta et al. 2000; DERM 2011; Evans and Sadler 2008; He et al. 2015; D’Odorico et al. 2013; Prathapar 1988; Qadir et al. 2013; Rengasamy 2006; Singh 2009; UNCTAD 2011; Wong et al. 2010 |
| Reduced soil compaction | Techniques to reduce soil compaction have variable impacts on GHG emissions but may benefit adaptation by improving soil climatic resilience. Since soil compaction is a driver of both desertification and land degradation, a reduction of soil compaction could benefit both. It could also help close yield gaps in rainfed crops. | Chamen et al. 2015; Epron et al. 2016; ITPS-FAO 2015; Hamza and Anderson 2005; Soane and van Ouwerkerk 1994; Tullberg et al. 2018 |
| Biochar addition to soil | The use of biochar increases carbon stocks in the soil. It can enhance yields in the tropics (but less so in temperate regions), thereby benefiting both adaptation and food security. Since it can improve soil water holding capacity and nutrient use efficiency, and can ameliorate heavy metal pollution and other impacts, it can benefit desertification and land degradation. The positive impacts could be tempered by additional pressure on land if large quantities of biomass are required as feedstock for biochar production. | Jeffery et al. 2017; Smith 2016; Sohi 2012; Woolf et al. 2010 |
| Fire management | The frequency and severity of large wildfires have increased around the globe in recent decades, which has impacted forest carbon budgets. Fire can cause various greenhouse gas emissions such as CO_2_, CH_4_, and N_2_O, and others such as CO, volatile organic carbon, and smoke aerosols. Fire management can reduce GHG emissions and can reduce haze pollution, which has significant health and economic impacts. Fire management helps to prevent soil erosion and land degradation and is used in rangelands to conserve biodiversity and to enhance forage quality. | Esteves et al. 2012; FAO 2006; Lin et al. 2017; O’Mara 2012; Rulli et al. 2006; Scasta et al. 2016; Seidl et al. 2014; Smith et al. 2014; Tacconi 2016; Valendik et al. 2011; Westerling et al. 2006; Whitehead et al. 2008; Yong and Peh 2016 |
| Reduced landslides and natural hazards | Management of landslides and natural hazards is important for adaptation and is a very important practice for managing land degradation, since landslides and natural hazards are among the most severe degradation processes. In countries where mountain slopes are planted with food crops, reduced landslides will help deliver benefits for food security. Most deaths caused due to different disasters have occurred in developing countries, in which poverty, poor education and health facilities, and other aspects of development increase exposure, vulnerability and risk. | Arnáez J et al. 2015; Campbell 2015; ITPS-FAO 2015; Gariano and Guzzetti 2016; Mal et al. 2018 |
| Reduced pollution including acidification | There are a few potential adverse side effects of reduction in air pollution to carbon sequestration in terrestrial ecosystems, because some forms of air pollutants can enhance crop productivity by increasing diffuse sunlight, compared to direct sunlight. Reactive N deposition could also enhance CO_2_ uptake in boreal forests and increase soil carbon pools to some extent. Air pollutants have different impacts on climate depending primarily on the composition, with some aerosols (and clouds seeded by them) increasing the reflection of solar radiation to space leading to net cooling, while others (e.g. black carbon and tropospheric ozone) having a net warming effect. Therefore, control of these different pollutants will have both positive and negative impacts on climate mitigation. | Anderson et al. 2017; Chum et al. 2013; Carter et al. 2015; Coakley; Maaroufi et al. 2015; Markandya et al. 2018; Melamed and Schmale 2016; Mostofa et al 2016.; Nemet et al. 2010; Ramanathan et al. 2001; Seinfeld and Pandis; Smith et al. 2015; UNEP 2017; Wild et al. 2012  UNEP and WMO 2011; Xu & Ramanathan, 2017; Xu et al., 2013 |
| Management of invasive species / encroachment | Exotic species are used in forestry where local indigenous forests cannot produce the type, quantity and quality of forest products required. Planted forests of exotic tree species make significant contributions to the economy and provide multiple products and Nature’s Contributions to People. In general, exotic species are selected to have higher growth rates than native species and produce more wood per unit of area and time. In 2015, the total area of planted forest with non-native tree species was estimated to around 0.5 Mkm^2^. Introduced species were dominant in South America, Oceania and Eastern and Southern Africa, where industrial forestry is dominant. The use of exotic tree species has played an important role in the production of roundwood, fibre, firewood and other forest products. The challenge is to manage existing and future plantation forests of alien trees to maximise current benefits, while minimising present and future risks and negative impacts, and without compromising future benefits. In many countries or regions, non-native trees planted for production or other purposes often lead to sharp conflicts of interest when they become invasive, and to negative impacts on Nature’s Contributions to People and nature conservation. | Brundu and Richardson 2016; Cossalter and Pye-Smith 2003; Dresner et al. 2015; Payn et al. 2015; Pimentel et al. 2005; Vilà et al. 2011 |
| Restoration and reduced conversion of coastal wetlands | Coastal wetland restoration and avoided coastal wetland impacts have the capacity to increase carbon sinks and can provide benefits by regulating water flow and preventing downstream flooding. Coastal wetlands provide a natural defence against coastal flooding and storm surges by dissipating wave energy, reducing erosion and by helping to stabilise shore sediments. Since large areas of global coastal wetlands are degraded, restoration could provide benefits land degradation. Since some areas of coastal wetlands are used for food production, restoration could displace food production and damage local food supply (Section 6.4.4), though some forms (e.g. mangrove restoration) can improve local fisheries. | Griscom et al. 2017; Lotze et al. 2006; Munang et al. 2014; Naylor et al. 2000 |
| Restoration and reduced conversion of peatlands | Avoided peat impacts and peatland restoration can provide significant mitigation, though restoration can lead to an increase in methane emissions, particularly in nutrient rich fens. There may also be benefits for climate adaptation by regulating water flow and preventing downstream flooding. Considering that large areas of global peatlands are degraded, peatland restoration is a key tool in addressing land degradation. Since large areas of tropical peatlands and some northern peatlands have been drained and cleared for food production, their restoration could displace food production and damage local food supply, potentially leading to adverse impacts on food security locally, though the global impact would be limited due to the relatively small areas affected. | Griscom et al. 2017; Jauhiainen et al. 2008; Limpens et al. 2008; Munang et al. 2014 |
| Biodiversity conservation | Biodiversity conservation measures interact with the climate system through many complex processes, which can have either positive or negative impacts. For example, establishment of protected areas can increase carbon storage in vegetation and soil, and tree planting to promote species richness and natural habitats can enhance carbon uptake capacity of ecosystems. Management of wild animals can influence climate *via* emissions of GHGs (from anaerobic fermentation of plant materials in the rumen), impacts on vegetation (*via* foraging), changes in fire frequency (as grazers lower grass and vegetation densities as potential fuels), and nutrient cycling and transport (by adding nutrients to soils). Conserving and restoring megafauna in northern regions also prevents thawing of permafrost and reduces woody encroachment, thus avoiding methane emissions and increases in albedo. Defaunation affects carbon storage in tropical forests and savannahs. In the tropics, the loss of mega-faunal frugivores is estimated be responsible for up to 10% reduction in carbon storage of global tropical forests. Frugivore rewilding programmes in the tropics are seen as carbon sequestration options that can be equally effective as tree planting schemes. Biodiversity conservation measures generally favour adaptation, but can interact with food security, land degradation or desertification. Protected areas for biodiversity reduce the land available for food production, and abundancies in some species like large animals can influence land degradation processes by grazing, trampling and compacting soil surfaces, thereby altering surface temperatures and chemical reactions affecting sediment and carbon retention. | Bello et al. 2015; Campbell et al. 2008; Cromsigt et al. 2018; Kapos et al. 2008; Osuri et al. 2016; Schmitz et al. 2018; Secretariat of the Convention on Biological Diversity 2008 |
| Enhanced weathering of minerals | Enhanced mineral weathering can remove atmospheric CO_2_. Since ground minerals can increase pH, there could be some benefits for efforts to prevent or reverse land degradation where acidification is the driver of degradation. Since increasing soil pH in acidified soils can increase productivity, the same effect could provide some benefit for food security. Minerals used for enhanced weathering need to be mined, and mining has large impacts locally, though the total area mined is likely to be small on the global scale. | Lenton 2010; Schuiling and Krijgsman 2006; Smith et al. 2016c; Taylor et al. 2016; Beerling et al. 2018 |
| Bioenergy and BECCS | Bioenergy and BECCS can compete for land and water with other uses. Increased use of bioenergy and BECCS can result in large expansion of cropland area, significant deforestation, and increased irrigation water use and water scarcity. Large-scale use of bioenergy can result in increased food prices and can lead to an increase in the population at risk of hunger. As a result of these effects, large-scale bioenergy and BECCS can have negative impacts for food security. Interlinkages of bioenergy and BECCS with climate change adaptation, land degradation, desertification, and biodiversity are highly dependent on local factors such as the type of energy crop, management practice, and previous land use. For example, intensive agricultural practices aiming to achieve high crop yields, as is the case for some bioenergy systems, may have significant effects on soil health, including depletion of soil organic matter, resulting in negative impacts on land degradation and desertification. However, with low inputs of fossil fuels and chemicals, limited irrigation, heat/drought tolerant species, using marginal land, biofuel programs can be beneficial to future adaptation of ecosystems. Planting bioenergy crops, like perennial grasses, on degraded land can increase soil carbon and ecosystem quality (including biodiversity), thereby helping to preserve soil quality, reverse land degradation, prevent desertification processes, and reduce food insecurity. These effects depend on the scale of deployment, the feedstock, the prior land use, and which other practices are included. Large-scale production of bioenergy can require significant amounts of land, increasing potential pressures for land conversion and land degradation. Low levels of bioenergy deployment require less land, leading to smaller effects on forest cover and food prices; however, these land requirements could still be substantial. In terms of feedstocks, some feedstocks, grown in some regions, may not need irrigation, and thus would not compete for water with food crops. Additionally, the use of residues or microalgae could limit competition for land and biodiversity loss; however, residues could result in land degradation or decreased soil organic carbon. Whether woody bioenergy results in increased competition for land or not is disputed in the literature, with some studies suggesting reduced competition and others suggesting enhanced. One study noted that this effect changes over time, with complementarity between woody bioenergy and forest carbon sequestration in the near-term, but increased competition for land with afforestation/reforestation in the long-term. | Baker et al. 2019; Calvin et al. 2014; Chaturvedi et al. 2013; Chum et al. 2011; Clarke et al. 2014; Correa et al. 2017; Creutzig et al. 2015; Dasgupta et al. 2014; Don et al. 2012; Edelenbosch et al. 2017; Edenhofer et al. 2011; FAO 2011; Favero and Mendelsohn 2014; Fujimori et al. 2018; Fuss et al. 2016, 2018; Hejazi et al. 2015; Kemper 2015; Kline et al. 2017; Lal 2014; Lotze-Campen et al. 2013; Mello et al. 2014; Muratori et al. 2016; Noble et al. 2014; Obersteiner et al. 2016; Popp et al. 2011c, 2014a, 2017; Riahi et al. 2017; Robertson et al. 2017; Sánchez et al. 2017; Searchinger et al. 2018; Sims et al. 2014; Slade et al. 2014; Smith et al. 2016c; Torvanger 2018; van Vuuren et al. 2011, 2015b, 2016; Wise et al. 2015; Tian et al. 2018; |

Table S2 Context, caveats and supporting evidence for value chain management-based practices considered in this study.

| Practice | Context and caveats | Supporting evidence |
| --- | --- | --- |
| Dietary change | A dietary shift away from meat can reduce greenhouse gas emissions, reduce cropland and pasture requirements, enhance biodiversity protection, and reduce mitigation costs. Additionally, dietary change can both increase potential for other land-based practices and reduce the need for them by freeing land. By decreasing pressure on land, demand reduction through dietary change could also allow for decreased production intensity, which could reduce soil erosion and provide benefits to a range of other environmental indicators such as deforestation and decreased use of fertiliser (N and P), pesticides, water and energy, leading to potential benefits for adaptation, desertification, and land degradation. | Aleksandrowicz et al. 2016; Bajželj et al. 2014; Bonsch et al. 2016; Erb et al. 2016; Godfray et al. 2010; Haberl et al. 2011; Havlík et al. 2014; Muller et al. 2017; Smith et al. 2013; Springmann et al. 2018; Stehfest et al. 2009; Tilman and Clark 2014; Wu et al. 2019 |
| Reduced post-harvest losses | Differences exist between farm food waste reduction technologies between small-scale agricultural systems and large-scale agricultural systems. A suite of options includes farm level storage facilities, trade or exchange processing technologies including food drying, onsite farm processing for value addition, and improved seed systems. For large scale agri-food systems, options include cold chains for preservation, processing for value addition and linkages to value chains that absorb the harvests almost instantly into the supply chain. In addition to the specific options to reduce food loss and waste, there are more systemic possibilities related to food systems. Improving and expanding the ‘dry chain’ can significantly reduce food losses at the household level. Dry chains are analogous to the cold chain and refers to the ‘initial dehydration of durable commodities to levels preventing fungal growth’ followed by storage in moisture-proof containers. Regional and local food systems are now being promoted to enable production, distribution, access and affordability of food. Reducing post-harvest losses has the potential to reduce emissions and could simultaneously reduce food costs and increase availability. The perishability and safety of fresh foods are highly susceptible to temperature increase. | Ansah et al. 2017; Bajželj et al. 2014; Billen et al. 2018; Bradford et al. 2018; Chaboud and Daviron 2017; Göbel et al. 2015; Gustavsson et al. 2011; Hengsdijk and de Boer 2017; Hodges et al. 2011; Ingram et al. 2016; Kissinger et al. 2018; Kumar and Kalita 2017; Ritzema et al. 2017; Sheahan and Barrett 2017; Wilhelm et al. 2016 |
| Reduced food waste (consumer or retailer) | Reducing food waste could lead to a reduction in cropland area and GHG emissions, resulting in benefits for mitigation. By decreasing pressure on land, food waste reduction could allow for decreased production intensity, which could reduce soil erosion and provide benefits to a range of other environmental indicators such as deforestation and decreases in use of fertiliser (N and P), pesticides, water and energy, leading to potential benefits for adaptation, desertification, and land degradation. | Alexander et al. 2016; Bajželj et al. 2014; Gustavsson et al. 2011; Kummu et al. 2012; Muller et al. 2017; Smith et al. 2013; Vermeulen et al. 2012b |
| Material substitution | Material substitution reduces carbon emissions both because the biomass sequesters carbon in materials while re-growth of forests can lead to continued sequestration, and because it reduces the demand for fossil fuels, delivering a benefit for mitigation. However, a potential trade-off exists between conserving carbon stocks and using forests for wood products. If the use of material for substitution was large enough to result in increased forest area, then the adverse side-effects for adaptation and food security would be similar to that of reforestation and afforestation. In addition, some studies indicate that wooden buildings, if properly constructed, could reduce fire risk compared to steel, creating a co-benefit for adaptation. The effects of material substitution on land degradation depend on management practice; some forms of logging can lead to increased land degradation. Long-term forest management with carbon storage in long-lived products also results in atmospheric carbon dioxide (CO_2_) removal. | Dugan et al. 2018; Eriksson et al. 2012; Gustavsson et al. 2006; Kauppi et al. 2018; Leskinen et al. 2018; McLaren 2012; Oliver and Morecroft 2014; Ramage et al. 2017; Sathre and O’Connor 2010; Smyth et al. 2014; Kurz et al. 2016; Miner 2010; Iordan et al. 2018 |
| Sustainable sourcing | Sustainable sourcing is expanding but accounts for only a small fraction of overall food and material production; many staple food crops do not have strong sustainability standards. Sustainable sourcing provides potential benefits for both climate mitigation and climate adaptation by reducing drivers of unsustainable land management, and by diversifying and increasing flexibility in the food system to climate stressors and shocks. Sustainable sourcing can lower expenditures of food processors and retailers by reducing losses. Adding value to products can extend a producer’s marketing season and provide unique opportunities to capture niche markets thereby increasing their adaptive capacity to climate change. Sustainable sourcing can also provide significant benefits for food security, while simultaneously creating economic alternatives for the poor. Sustainable sourcing programmes often also have positive impacts on the overall efficiency of the food supply chain and can create closer and more direct links between producers and consumers. In some cases, processing of value-added products could lead to higher emissions or demand of resources in the food system, potentially leading to small adverse impacts on land degradation and desertification land challenges. | Accorsi et al. 2017; Bajželj et al. 2014; Bustamante et al. 2014; Clark and Tilman 2017; Garnett 2011; Godfray et al. 2010; Hertel 2015; Ingram et al. 2016; James and James 2010; Muller et al. 2017; Tilman and Clark 2014; Springer et al. 2015; Tayleur et al. 2017 |
| Management of supply chains | Successful implementation of supply chain management practices is dependent on organisational capacity, the agility and flexibility of business strategies, the strengthening of public-private policies and effectiveness of supply-chain governance. Existing practices include a) greening supply chains (e.g. utilising products and services with a reduced impact on the environment and human health), b) adoption of specific sustainability instruments among agri-food companies (e.g. eco-innovation practices ), c) adopting emission accounting tools (e.g. carbon and water foot-printing), and d) implementing “demand forecasting” strategies (e.g. changes in consumer preference for 'green' products). In terms of food supply, measures to improve stability in traded markets can include: 1) financial and trade policies, such as reductions on food taxes and import tariffs; 2) shortening food supply chains (SFSCs); 3) increasing food production; 4) designing alternative distribution networks; 5) increasing food market transparency and reducing speculation in futures markets; 6) increasing storage options; and 7) increasing subsidies and food-based safety nets. | Barthel and Isendahl 2013; Haggblade et al. 2017; Lewis and Witham 2012; Michelini et al. 2018; Minot 2014; Mundler and Rumpus 2012; Tadasse et al. 2016; Wheeler and von Braun 2013; Wilhelm et al. 2016; Wodon and Zaman 2010; The World Bank 2011 |
| Enhanced urban food systems | Urban territorial areas have a potential to reduce GHG emissions through improved food systems to reduce vehicle miles of food transportation, localised carbon capture and food waste reduction. The benefits of Urban food forests that are intentionally planted woody perennial food producing species, are also cited for their carbon sequestration potentials. However, new urban food systems may have diverse unexpected adverse side-effects with climate systems, such as lower efficiencies in food supply and higher costs than modern large-scale agriculture. Diversifying markets, considering value added products in the food supply system may help to improve food security by increasing its economic performance and revenues to local farmers. | Akhtar et al. 2016; Benis and Ferrão 2017; Brinkley et al. 2013; Chappell et al. 2016; Goldstein et al. 2016; Kowalski and Conway 2018; Lee-Smith 2010; Barthel and Isendahl 2013; Lwasa et al. 2014, 2015; Revi et al. 2014; Specht et al. 2014; Tao et al. 2015; UPAF (date) |
| Improved food processing and retailing | Improved food processing and retailing can provide benefits for climate mitigation since GHG-friendly foods can reduce agri-food GHG emissions from transportation, waste and energy use. In cases where climate extremes and natural disasters disrupt supply chain networks, improved food processing and retailing can benefit climate adaptation by buffering the impacts of changing temperature and rainfall patterns on upstream agricultural production. It can provide benefits for food security by supporting healthier diets and reducing food loss and waste. Successful implementation is dependent on organisational capacity, the agility and flexibility of business strategies, the strengthening of public-private policies and effectiveness of supply-chain governance. | Avetisyan et al. 2014; Garnett et al. 2013; Godfray et al. 2010; Mohammadi et al. 2014; Porter et al. 2016; Ridoutt et al. 2016; Song et al. 2017 |
| Improved energy use in food systems | Transformation to low carbon technologies such as renewable energy and energy efficiency can offer opportunities for significant climate change mitigation by providing a substitute to transport fuel (for example) that could benefit marginal agricultural resources, while simultaneously contributing to long term economic growth. In poorer nations, increased energy efficiency in agricultural value-added production, in particular, can provide large mitigation benefits. Under certain scenarios, the efficiency of agricultural systems can stagnate and could exert pressure on grasslands and rangelands, thereby impacting land degradation and desertification. Rebound effects can also occur, with adverse impacts on emissions. | Al-Mansour and Jejcic 2017; Baptista et al. 2013; Gunatilake et al. 2014; Begum et al. 2015; Jebli and Youssef 2017; van Vuuren et al. 2017b |

Table S3 Context, caveats and supporting evidence for risk management-based practices considered in this study.

| Practice | Context and caveats | Supporting evidence |
| --- | --- | --- |
| Management of urban sprawl | The prevention of uncontrolled urban sprawl may provide adaptation co-benefits, but adverse side effects for adaptation might arise due to restricted ability of people to move in response to climate change. | Barbero-Sierra et al. 2013; Bren d’Amour et al. 2016; Cai et al. 2013; Chen 2007; Francis et al. 2012; Gibson et al. 2015; Lee et al. 2015; Qian et al. 2015; Shen et al. 2017; Tan et al. 2009 |
| Livelihood diversification | Livelihood diversification offers benefits for desertification and land degradation, particularly through non-traditional crops or agroforestry systems which improve soil. It can increase on-farm biodiversity due to these investments in more ecosystem-mimicking production systems, like agroforestry and polycultures. Diversification into non-agricultural fields, such as wage labour or trading, is increasingly favoured by farmers as a low-cost strategy, particularly to respond to increasing climate risks. | Adger 1999; Ahmed and Stepp 2016; Antwi-Agyei et al. 2014; Barrett et al. 2001; Berman et al. 2012; Bryceson 1999; DiGiano and Racelis 2012; Ellis 1998, 2008; Ngigi et al. 2017; Rakodi 1999; Thornton and Herrero 2014; Little et al. 2001 |
| Use of local seeds | Use of local seeds is important in the many parts of the developing world that do not rely on commercial seed inputs. Promotion of local seed saving initiatives can include seed networks, banks and exchanges, and non-commercial open source plant breeding. These locally developed seeds can both help protect local agrobiodiversity and can often be more climate resilient than generic commercial varieties, although the impacts on food security and overall land degradation are inconclusive. | Bowman 2015; Campbell and Veteto 2015; Coomes et al. 2015; Kloppenberg 2010; Luby et al. 2015; van Niekerk and Wynberg 2017; Patnaik et al. 2017; Reisman 2017; Vasconcelos et al. 2013; Wattnem 2016 |
| Disaster risk management | Disaster risk management is dependent on how it is used; effective approaches must be ‘end-to-end,’ both reaching communities at risk and supporting and empowering vulnerable communities to take appropriate action. The most effective early warning systems are not simply technical systems of information dissemination, but utilise and develop community capacities, create local ownership of the system, and are based on a shared understanding of needs and purpose. Tapping into existing traditional or local knowledge has also been recommended to reduce vulnerability. | Ajibade and McBean 2014; Alessa et al. 2016; Bouwer et al. 2014; Carreño et al. 2007; Cools et al. 2016; Djalante et al. 2012; Garschagen 2016; Maskrey 2011; Mercer 2010; Sternberg and Batbuyan 2013; Thomalla et al. 2006; Vogel and O’Brien 2006; Schipper and Pelling 2006 |
| Risk sharing instruments | Locally developed risk pooling measures show general positive impacts on household livelihoods. However, more commercial approaches have mixed effects. Commercial crop insurance is highly subsidised in much of the developed world. Index insurance programmes have often failed to attract sufficient buyers or have remained financially unfeasible for commercial insurance sellers. The overall impact of index insurance on food production supply and access has also not been assessed. Traditional crop insurance has generally been seen as positive for food security as it leads to expansion of agricultural production areas and increased food supply. However, insurance may ‘mask’ truly risky agriculture and prevent farmers from seeking less risky strategies. Insurance can provide perverse incentives for farmers to bring additional lands into production, leading to greater risk of degradation. | Akter et al. 2016; Annan and Schlenker 2015; Claassen et al. 2011; Fenton et al. 2017; Giné et al. 2008; Goodwin and Smith 2003; Hammill et al. 2008; Havemenn and Muccione 2011; Jaworski 2016; Meze-Hausken et al. 2009; Morduch and Sharma 2002; Bhattamishra and Barrett 2010; Peterson 2012; Sanderson et al. 2013; Skees and Collier 2012; Smith and Glauber 2012 |

Table S4 Examples of overarching frameworks that consist of a range of practices, showing how various practices contribute to the overarching frameworks

| **Framework (definition used)** | Nature based solutions (IUCN) | Agro-ecology (FAO) | Climate smart agriculture (FAO) | Ecosystem based adaptation (CBD) | Conservation agriculture (FAO) | Community based adaptation (IIED) | Integrated landscape management including integrated coastal zone management (FAO) | Precision agriculture (FAO) | Sustainable forest management (UN) | Sustainable intensification (FAO) | Organic agriculture (FAO) |
| --- | --- | --- | --- | --- | --- | --- | --- | --- | --- | --- | --- |
| **Practices based on land management** |  | | | | | | | | | | |
| Increased food productivity |  |  | x |  | x |  | x | x |  | x |  |
| Improved cropland management |  | x | x |  | x | x | x | x |  | x | X |
| Improved grazing land management |  | x | x | x |  | x | x |  |  | x | X |
| Improved livestock management |  | x | x |  |  | x | x |  |  | x | X |
| Agroforestry |  | x | x | x |  | x | x |  |  | x | X |
| Agricultural diversification |  | x | x |  |  |  | x |  |  | x | X |
| Reduced grassland conversion to cropland |  | x |  | x |  | x | x |  |  |  |  |
| Integrated water management | x | x | x | x | x | x | x | x |  | x | X |
| Improved forest management | x |  |  | x |  | x | x |  | x |  |  |
| Reduced deforestation and degradation |  | x |  | x |  | x | x |  |  |  |  |
| Reforestation and forest restoration | x | x |  | x |  | x | x |  | x |  |  |
| Afforestation |  |  |  | x |  | x | x |  |  |  |  |
| Increased soil organic carbon content |  | x | x | x | x |  | x |  |  | x | X |
| Reduced soil erosion |  | x | x | x | x |  | x |  |  | x | X |
| Reduced soil salinisation |  | x | x | x | x |  | x | x |  | x | X |
| Reduced soil compaction |  | x | x | x | x |  | x |  |  | x | X |
| Biochar addition to soil |  | x | x |  |  |  |  |  |  |  |  |
| Fire management |  | x | x | x |  | x | x |  | x |  |  |
| Reduced landslides and natural hazards |  | x | x | x |  | x | x |  |  |  |  |
| Reduced pollution including acidification |  |  |  |  |  |  | x | x |  | x | X |
| Management of invasive species / encroachment | x | x |  | x |  | x | x |  | x |  | X |
| Restoration and reduced conversion of coastal wetlands |  | x |  | x |  | x | x |  |  |  |  |
| Restoration and reduced conversion of peatlands |  | x | x | x |  | x | x |  |  |  |  |
| Biodiversity conservation | x | x | x | x | x | x | x |  | x | x |  |
| Enhanced weathering of minerals |  |  |  |  |  |  |  |  |  |  |  |
| Bioenergy and BECCS |  |  |  |  |  |  | x |  |  |  |  |
| **Practices based on value chain management** |  | | | | | | | | | | |
| Dietary change |  | x |  |  |  |  |  |  |  |  | x |
| Reduced post-harvest losses |  | x | x |  |  | x |  | x |  |  | x |
| Reduced food waste (consumer or retailer) |  | x |  |  |  |  |  |  |  |  |  |
| Material substitution |  |  |  |  |  |  |  |  |  |  |  |
| Sustainable sourcing |  | x | x |  |  | x | x |  |  |  | x |
| Management of supply chains |  | x | x |  |  |  |  |  |  |  |  |
| Enhanced urban food systems |  | x | x |  |  | x | x | x |  | x | x |
| Improved food processing and retailing |  | x |  |  |  |  |  |  |  |  |  |
| Improved energy use in food systems |  | x | x |  | x |  |  | x |  | x |  |
| **Practices based on risk management** |  | | | | | | | | | | |
| Management of urban sprawl |  |  |  | x |  | x | x |  |  |  |  |
| Livelihood diversification |  | x | x | x |  | x | x | x |  |  |  |
| Use of local seeds | x | x | x | x |  | x | x |  |  |  |  |
| Disaster risk management | x |  |  | x |  | x | x |  |  |  | x |
| Risk sharing instruments |  |  |  |  |  |  |  |  |  | x |  |

Table S5 Mapping of practices considered in this study and the IPCC SR1.5 (2017)

| **Practices considered in this study** | **IPCC SR1.5 Options** |
| --- | --- |
| Afforestation | Afforestation |
| Reforestation and forest restoration | Reforestation and reduced land degradation and forest restoration |
| Agricultural diversification | Mixed crop-livestock systems |
| Agroforestry | Agroforestry and silviculture |
| Biochar addition to soil | Biochar |
| Biodiversity conservation | Biodiversity conservation |
| Bioenergy and BECCS | Biomass use for energy production with carbon capture and sequestration (BECCS) (through combustion, gasification, or fermentation) |
| Dietary change | Dietary changes, reducing meat consumption |
| Disaster risk management | Climate services |
|  | Community-based adaptation |
| Enhanced urban food systems | Urban and peri-urban agriculture and forestry |
| Enhanced weathering of minerals | Mineralisation of atmospheric CO2 through enhanced weathering of rocks |
| Fire management | Fire management and (ecological) pest control |
| Improved forest management | Forest management |
| Improved cropland management | Methane reductions in rice paddies |
| Improved cropland management | Nitrogen pollution reductions, e.g., by fertiliser reduction, increasing nitrogen fertiliser efficiency, sustainable fertilisers |
|  | Precision agriculture |
|  | Conservation agriculture |
| Improved food processing and retailing |  |
| Improved grazing land management | Livestock and grazing management, for example, methane and ammonia reductions in ruminants through feeding management or feed additives, or manure management for local biogas production to replace traditional biomass use |
| Improved livestock management |  |
|  | Manure management |
| Increased energy efficiency in food systems |  |
| Increased food productivity | Increasing agricultural productivity |
| Increased soil organic carbon content | Changing agricultural practices enhancing soil carbon |
|  | Soil carbon enhancement, enhancing carbon sequestration in biota and soils, e.g. with plants with high carbon sequestration potential (also AFOLU measure) |
| Integrated water management | Irrigation efficiency |
| Livelihood diversification |  |
| Management of invasive species / encroachment |  |
| Management of supply chains |  |
| Management of urban sprawl | Urban ecosystem services |
|  | climate resilient land use |
| Material substitution | Material substitution of fossil CO_2_ with bio-CO_2_ in industrial application (e.g. the beverage industry) |
|  | Carbon Capture and Usage – CCU; bioplastics (bio-based materials replacing fossil fuel uses as feedstock in the production of chemicals and polymers), carbon fibre |
| Reduced soil erosion |  |
| Reduced soil compaction |  |
| Reduced deforestation | Reduced deforestation, forest protection, avoided forest conversion |
| Reduced food waste (consumer or retailer) | Reduction of food waste (incl. reuse of food processing waste for fodder) |
| Reduced grassland conversion to cropland |  |
| Reduced landslides and natural hazards |  |
| Reduced pollution including acidification | Reduced air pollution |
| Reduced post-harvest losses |  |
| Reduced soil salinisation |  |
| Restoration and reduced conversion of coastal wetlands | Managing coastal stress |
|  | Restoration of wetlands (e.g., coastal and peat-land restoration, blue carbon) and wetlands management |
| Restoration and reduced conversion of peatlands |  |
| Risk sharing instruments | Risk sharing |
| Sustainable sourcing |  |
| Use of local seeds |  |

Additional References for Supplementary Material (see also main paper)

Accorsi, R., Gallo, A., & Manzini, R. (2017). A climate driven decision-support model for the distribution of perishable products. Journal of Cleaner Production, 165, 917–929. <https://doi.org/10.1016/j.jclepro.2017.07.170>

Adger, W. N. (1999). Social vulnerability to climate change and extremes in coastal Vietnam. World Development, 27(2), 249–269. <https://doi.org/10.1016/S0305-750X(98)00136-3>

Ahmed, S., & Stepp, J. R. (2016). Beyond yields: climate change effects on specialty crop quality and agroecological management. *Elementa: Science of the Anthropocene*, *4*, 92. https://doi.org/10.12952/journal.elementa.000092

Ajibade, I., & McBean, G. (2014). Climate extremes and housing rights: a political ecology of impacts, early warning and adaptation constraints in Lagos slum communities. *Geoforum*, *55*, 76–86. https://doi.org/10.1016/j.geoforum.2014.05.005

Akhtar, P., Tse, Y., Khan, Z., & Rao-Nicholson, R. (2016). Data-driven and adaptive leadership contributing to sustainability: global agri-food supply chains connected with emerging markets. *International Journal of Production Economics*, *181*, 392–401. Retrieved from https://www.sciencedirect.com/science/article/pii/S0925527315005125

Akter, S., Krupnik, T. J., Rossi, F., & Khanam, F. (2016). The influence of gender and product design on farmers’ preferences for weather-indexed crop insurance. *Global Environmental Change*, *38*, 217–229. https://doi.org/10.1016/j.gloenvcha.2016.03.010

Al-Mansour F, & Jejcic V. (2017). A model calculation of the carbon footprint of agricultural products: the case of Slovenia. *Energy*, *136*, 7–15. https://doi.org/10.1016/j.energy.2016.10.099

Alessa, L., Kliskey, A., Gamble, J., Fidel, M., Beaujean, G., & Gosz, J. (2016). The role of Indigenous science and local knowledge in integrated observing systems: moving toward adaptive capacity indices and early warning systems. *Sustainability Science*, *11*(1), 91–102. https://doi.org/10.1007/s11625-015-0295-7

Anderson, C. M., Field, C. B., & Mach, K. J. (2017). Forest offsets partner climate-change mitigation with conservation. *Frontiers in Ecology and the Environment*, *15*(7), 359–365. https://doi.org/10.1002/fee.1515

Ansah, I. G. K., Tetteh, B. K. D., & Donkoh, S. A. (2017). Determinants and income effect of yam postharvest loss management: evidence from the Zabzugu District of Northern Ghana. *Food Security*, *9*(3), 611–620. https://doi.org/10.1007/s12571-017-0675-1

Archer, S., Davies, K., Fulbright, T., McDaniel, K., Wilcox, B., Predick, K., & Briske, D. (2011). Brush management as a rangeland conservation strategy: a critical evaluation. In *Conservation benefits of rangeland practices: assessment, recommendations, and knowledge gaps.* (pp. 105–170). Retrieved from https://www.ars.usda.gov/research/publications/publication/?seqNo115=268913

Arora, V. K., & Montenegro, A. (2011). Small temperature benefits provided by realistic afforestation efforts. *Nature Geoscience*, *4*, 514–518. https://doi.org/10.1038/ngeo1182

Avetisyan, M., Hertel, T., & Sampson, G. (2014). Is local food more environmentally friendly? The GHG emissions impacts of consuming imported versus domestically produced food. *Environmental and Resource Economics*, *58*(3), 415–462. https://doi.org/10.1007/s10640-013-9706-3

Balmford, A., Amano, T., Bartlett, H., Chadwick, D., Collins, A., Edwards, D., Field, R., Garnsworthy, P., Green, R., Smith, P., Waters, H. (2018). The environmental costs and benefits of high-yield farming. *Nature Sustainability*, *1*(9), 477. https://doi.org/10.1038/s41893-018-0138-5

Baptista, F., Silva, L. L., De Visser, C., Gołaszewski, J., Meyer-Aurich, A., Briassoulis, D., Mikkola, H., Murcho, D. (2013). Energy efficiency in agriculture. In *Complete communications of the 5th International Congress on Energy and Environment Engineering and Management 2013,Lisbon, 17-19 Julho.* Retrieved from https://dspace.uevora.pt/rdpc/bitstream/10174/8648/1/Energy_efficiency_in_agriculture_Lisbon.pdf

Barlow, J., Lennox, G. D., Ferreira, J., Berenguer, E., Lees, A. C., . MacNally, R, Thomson, J.R., de Barros Ferraz, S.F., Louzada, J., Oliveira, V.H.F., Parry, L., de Castro Solar, R.R., Vieira, I.C.G., Aragão, L.E.O.C., Begotti, R.A., Braga, R.F., Cardoso, T.M., R.C. de Oliveira Jr, R.C., Souza Jr, C.M., Moura, N.G., Nunes, S.S., Siqueira, J.V., Pardini, R., Silveira, J.M., Vaz-de-Mello, F.Z., Veiga, R.C.S., Venturieri, A., Gardner, T. A. (2016). Anthropogenic disturbance in tropical forests can double biodiversity loss from deforestation. *Nature*, *535*(7610), 144–147. https://doi.org/10.1038/nature18326

Barthel, S., & Isendahl, C. (2013). Urban gardens, agriculture, and water management: sources of resilience for long-term food security in cities. *Ecological Economics*, *86*, 224–234. https://doi.org/10.1016/J.ECOLECON.2012.06.018

Bayrak, M., Marafa, L., Bayrak, M. M., & Marafa, L. M. (2016). Ten years of REDD+: a critical review of the impact of REDD+ on forest-dependent communities. *Sustainability*, *8*(7), 620. https://doi.org/10.3390/su8070620

Begum, R., Sohag, K., Abdullah, S., & Jaafar, M. (2015). CO2 emissions, energy consumption, economic and population growth in Malaysia. *Renewable and Sustainable Energy Reviews*, *41*, 594–601. https://doi.org/10.1016/j.rser.2014.07.205

Bello, C., Galetti, M., Pizo, M. A., Magnago, L. F. S., Rocha, M. F., Lima, R. A. F., Peres, C.A., Ovaskainen, O., Jordano, P. (2015). Defaunation affects carbon storage in tropical forests. *Science Advances*, *1*(11), e1501105. https://doi.org/10.1126/sciadv.1501105

Benjamin, E. O., Ola, O., & Buchenrieder, G. (2018). Does an agroforestry scheme with payment for ecosystem services (PES) economically empower women in sub-Saharan Africa? *Ecosystem Services*, *31*, 1–11. https://doi.org/10.1016/j.ecoser.2018.03.004

Berman, R., Quinn, C., & Paavola, J. (2012). Thea role of institutions in the transformation of coping capacity to sustainable adaptive capacity. *Environmental Development*, *2*, 86–100. https://doi.org/10.1016/j.envdev.2012.03.017

Bestelmeyer, B., & Briske, D. (2012). Grand challenges for resilience-based management of rangelands. *Rangeland Ecology and Management*, *65*(6), 654–663. https://doi.org/10.2111/REM-D-12-00072.1

Bhattamishra, R., & Barrett, C. B. (2010). Community-based risk management arrangements: a review. *World Development*, *38*, 923–932. https://doi.org/10.1016/j.worlddev.2009.12.017

Billen, G., Lassaletta, L., Garnier, J., Noë, J. L., Aguilera, E., & Sanz-Cobena, A. (2018). Opening to distant markets or local reconnection of agro-food systems? Environmental consequences at regional and global scales. In *Agroecosystem Diversity* (pp. 391–413). https://doi.org/10.1016/B978-0-12-811050-8.00025-X

Bonan, G. B. (2008). Forests and climate change: forcings, feedbacks, and the climate benefits of forests. *Science (New York, N.Y.)*, *320*(5882), 1444–1449. https://doi.org/10.1126/science.1155121

Bonsch, M., Humpenöder, F., Popp, A., Bodirsky, B., Dietrich, J. P., Rolinski, S., Biewald, A., Lotze-Campen, H., Weindl, I., Gerten, D., Stevanovic, M. (2016). Trade-offs between land and water requirements for large-scale bioenergy production. *GCB Bioenergy*, *8*(1), 11–24. https://doi.org/10.1111/gcbb.12226

Bouwer, L. M., Papyrakis, E., Poussin, J., Pfurtscheller, C., & Thieken, A. H. (2014). The costing of measures for natural hazard mitigation in Europe. *Natural Hazards Review*, *15*(4), 4014010. https://doi.org/10.1061/(ASCE)NH.1527-6996.0000133

Bowman, A. (2015). Sovereignty, risk and biotechnology: Zambia’s 2002 GM controversy in retrospect. *Development and Change*, *46*(6), 1369–1391. https://doi.org/10.1111/dech.12196

Bradford, K. J., Dahal, P., Van Asbrouck, J., Kunusoth, K., Bello, P., Thompson, J., & Wu, F. (2018). The dry chain: reducing postharvest losses and improving food safety in humid climates. *Trends in Food Science and Technology*, *71*, 84–93. https://doi.org/10.1016/j.tifs.2017.11.002

Brindha, K., & Pavelic, P. (2016). Identifying priority watersheds to mitigate flood and drought impacts by novel conjunctive water use management. *Mitigation and Adaptation Strategies for Global Change*, *75*, 399. https://doi.org/10.1007/s12665-015-4989-z

Brinkley, C., Birch, E., & Keating, A. (2013). Feeding cities: Charting a research and practice agenda towards food security. *Journal of Agriculture, Food Systems, and Community Development*, *3*(4), 81–87. https://doi.org/10.5304/jafscd.2013.034.008

Briske, D. D., Joyce, L. A., Polley, H. W., Brown, J. R., Wolter, K., Morgan, J. A., McCarl, B. A.,Bailey, D. W. (2015). Climate-change adaptation on rangelands: linking regional exposure with diverse adaptive capacity. *Frontiers in Ecology and the Environment*, *13*(5), 249–256. https://doi.org/10.1890/140266

Brundu, G., & Richardson, D. M. (2016). Planted forests and invasive alien trees in Europe: a code for managing existing and future plantings to mitigate the risk of negative impacts from invasions. *NeoBiota*, *30*, 5–47. https://doi.org/10.3897/neobiota.30.7015

Bryan, E., Deressa, T. T., Gbetibouo, G. A., & Ringler, C. (2009). Adaptation to climate change in Ethiopia and South Africa: options and constraints. *Environmental Science & Policy*, *12*(4), 413–426. https://doi.org/10.1016/J.ENVSCI.2008.11.002

Bryceson, D. F. (1999). African rural labour, income diversification & livelihood approaches: a long‐term development perspective. *Review of African Political Economy*, *26*(80), 171–189. https://doi.org/10.1080/03056249908704377

Bustamante, M., Robledo-Abad, C., Harper, R., Mbow, C., Ravindranat, N. H., Sperling, F., Haberl, H., de Siqueira Pinto, A., Smith, P. (2014). Co-benefitsc, trade-offs, barriers and policies for greenhouse gas mitigation in the agriculture, forestry and other land use (AFOLU) sector. *Global Change Biology*, *20*(10), 3270–3290. https://doi.org/10.1111/gcb.12591

Cai, H., Yang, X., & Xu, X. (2013). Spatiotemporal patterns of urban encroachment on cropland and its impacts on potential agricultural productivity in China. *Remote Sensing*, *5*(12), 6443–6460. https://doi.org/10.3390/rs5126443

Campbell, B. C., & Veteto, J. R. (2015). Free seeds and food sovereignty: anthropology and grassroots agrobiodiversity conservation strategies in the US South. *Journal of Political Ecology*, *22*, 445–465. https://doi.org/10.2458/v22i1.21118

Caplow, S., Jagger, P., Lawlor, K., & Sills, E. (2011). Evaluating land use and livelihood impacts of early forest carbon projects: Lessons for learning about REDD+. *Environmental Science and Policy*, *14*(2), 152–167. https://doi.org/10.1016/j.envsci.2010.10.003

Carreño, M. L., Cardona, O. D., & Barbat, A. H. (2007). A disaster risk management performance index. *Natural Hazards*, *41*(1), 1–20. https://doi.org/10.1007/s11069-006-9008-y

Carter, D. R., Fahey, R. T., Dreisilker, K., Bialecki, M. B., & Bowles, M. L. (2015). Assessing patterns of oak regeneration and C storage in relation to restoration-focused management, historical land use, and potential trade-offs. *Forest Ecology and Management*, *343*, 53–62. https://doi.org/10.1016/j.foreco.2015.01.027

Chaboud, G., & Daviron, B. (2017). Food losses and waste: navigating the inconsistencies. *Global Food Security*, *12*, 1–7. https://doi.org/10.1016/j.gfs.2016.11.004

Chappell, M. J., Moore, J. R., & Heckelman, A. A. (2016). Participation in a city food security program may be linked to higher ant alpha- and beta-diversity: an exploratory case from Belo Horizonte, Brazil. *Agroecology and Sustainable Food Systems*, *40*(8), 804–829. https://doi.org/10.1080/21683565.2016.1160020

Cheesman, S., Thierfelder, C., Eash, N. S., Kassie, G. T., & Frossard, E. (2016). Soil carbon stocks in conservation agriculture systems of Southern Africa. *Soil and Tillage Research*, *156*, 99–109. https://doi.org/10.1016/J.STILL.2015.09.018

Chen, L., Wang, J., Wei, W., Fu, B., & Wu, D. (2010). Effects of landscape restoration on soil water storage and water use in the Loess Plateau Region, China. *Forest Ecology and Management*, *259*(7), 1291–1298. https://doi.org/10.1016/J.FORECO.2009.10.025

Cherubini, F., Vezhapparambu, S., Bogren, W., Astrup, R., & Strømman, A. H. (2017). Spatial, seasonal, and topographical patterns of surface albedo in Norwegian forests and cropland. *International Journal of Remote Sensing*, *38*(16), 4565–4586. https://doi.org/10.1080/01431161.2017.1320442

Chum, H., Faaij, A., Moreira, J., Berndes, G., Dhamija, P., Dong, H., Gabrielle, B., Eng, A.G., Lucht, W., Mapako, M., Cerutti, O.M., McIntyre, T., Minowa, T., Pingoud, K. (2011). Bioenergy. In O. Edenhofer, R. Pichs-Madruga, Y. Sokona, K. Seyboth, P. Matschoss, S. Kadner, K. Seyboth, A. Adler, I. Baum, S. Brunner, P. Eickemeier, B. Kriemann, J. Savolainen, S. Schlöme, C. von Stechow, T. Zwickel, J. C. Minx. (Eds.), *IPCC Special Report on Renewable Energy Sources and Climate Change Mitigation* (pp. 209–332). Retrieved from https://www.ipcc.ch/report/renewable-energy-sources-and-climate-change-mitigation/bioenergy/

Ciais, P., Sabine, C., Bala, G., Bopp, L., Brovkin, V., Canadell, J., Chhabra, A., DeFries, R., Galloway, J., Heimann, M., C. Jones, Le Quéré, C., Myneni, R. B., Piao, S., Thornton, P. (2013). Carbon and other biogeochemical cycles. In *Climate Change 2013: The Physical Science Basis. Contribution of Working Group I to the Fifth Assessment Report of the Intergovernmental Panel on Climate Change* (pp. 465–570). https://doi.org/10.1017/CBO9781107415324.015

Coakley, J. (2005). Atmospheric physics: reflections on aerosol cooling. *Nature*, *438*(7071), 1091–1092. https://doi.org/10.1038/4381091a

Cools, J., Innocenti, D., & O’Brien, S. (2016). Lessons from flood early warning systems. *Environmental Science & Policy*, *58*, 117–122. https://doi.org/10.1016/J.ENVSCI.2016.01.006

Correa, D. F., Beyer, H. L., Possingham, H. P., Thomas-Hall, S. R., & Schenk, P. M. (2017). Biodiversity impacts of bioenergy production: microalgae vs. first generation biofuels. *Renewable and Sustainable Energy Reviews*, *74*, 1131–1146. https://doi.org/10.1016/J.RSER.2017.02.068

Cossalter, C., & Pye-Smith, C. (2003). *Fast-wood forestry: myths and realities*. Retrieved from https://books.google.com/books?hl=en&lr=&id=fu3uciRDD2UC&oi=fnd&pg=PR4&dq=Cossalter+C.,+Pye-Smith+C.+Fast-Wood+Forestry:+Myths+and+Realities.+–+Center+for+International+Forestry+Research,+Jakarta,+2003.+60+&ots=ZIDhBWj5Hu&sig=EkcdWvuh0zjHsC6PVgWYLid8VYc

Creutzig, F., Ravindranath, N. H., Berndes, G., Bolwig, S., Bright, R., Cherubini, F., Chum, H., Corbera, E., Delucchi, M., Faaij, A., Fargione, J., Haberl, H., Heath, G., Lucon, O., Plevin, R., Popp, A., Robledo-Abad, C., Rose, S., Smith, P., Stromman, A., Suh, S., Masera, O. (2015). Bioenergy-c and climate change mitigation: An assessment. *GCB Bioenergy*, *7*(5), 916–944. https://doi.org/10.1111/gcbb.12205

Curtis, P. G., Slay, C. M., Harris, N. L., Tyukavina, A., & Hansen, M. C. (2018). Classifying drivers of global forest loss. *Science*, *361*(6407), 1108–1111. https://doi.org/10.1126/science.aau3445

D’Amato, A. W., Bradford, J. B., Fraver, S., & Palik, B. J. (2011). Forest management for mitigation and adaptation to climate change: Insights from long-term silviculture experiments. *Forest Ecology and Management*, *262*(5), 803–816. https://doi.org/10.1016/j.foreco.2011.05.014

D’Odorico, P., He, Y., Collins, S., De Wekker, S. F. J., Engel, V., & Fuentes, J. D. (2013). Vegetation-microclimate feedbacks in woodland-grassland ecotones. *Global Ecology and Biogeography*, *22*(4), 364–379. https://doi.org/10.1111/geb.12000

de Moraes Sá, J. C., Lal, R., Cerri, C. C., Lorenz, K., Hungria, M., & de Faccio Carvalho, P. C. (2017). Low-carbon agriculture in South America to mitigate global climate change and advance food security. *Environment International*, *98*, 102–112. https://doi.org/10.1016/J.ENVINT.2016.10.020

de Ruiter, H., Macdiarmid, J., Lynd, L., Smith, P., Matthews, R., & Kastner, T. (2017). Total global agricultural land footprint associated with UK food supply 1986-2011. In *Global environmental change*. Retrieved from http://agris.fao.org/agris-search/search.do?recordID=US201700147811

den Herder, M., Moreno, G., Mosquera-Losada, R. M., Palma, J. H. N., Sidiropoulou, A., Santiago-Freijanes, J. J., Crous-Duran, J., Paulo, J.A., Tomé, M., Pantera, A., Papanastasis, V. P., Mantzanas, K., Pachana, P., Papadopoulos, A., Plieninger, T., Burgess, P. J. (2017). Current extent and stratification of agroforestry in the European Union. *Agriculture, Ecosystems & Environment*, *241*, 121–132. https://doi.org/10.1016/j.agee.2017.03.005

DERM. (2011). *Salinity management handbook Second edition*. Brisbane, Australia.

Derpsch, R., Friedrich, T., Kassam, A., & Li, H. (2010). Current status of adoption of no-till farming in the world and some of its main benefits. *International Journal of Agricultural and Biological Engineering*, *3*(1), 1–25. https://doi.org/10.25165/IJABE.V3I1.223

DiGiano, M. L., & Racelis, A. E. (2012). Robustness, adaptation and innovation: forest communities in the wake of Hurricane Dean. *Applied Geography*, *33*, 151–158. https://doi.org/10.1016/j.apgeog.2011.10.004

Djalante, R., Thomalla, F., Sinapoy, M. S., & Carnegie, M. (2012). Building resilience to natural hazards in Indonesia: progress and challenges in implementing the Hyogo Framework for Action. *Natural Hazards*, *62*(3), 779–803. https://doi.org/10.1007/s11069-012-0106-8

Don, A., Osborne, B., Hastings, A., Skiba, U., Carter, M. S., Drewer, J., Flessa, H., Freibauer, A., Hyvönen, N., Jones, M.B., Lanigan, G.J., Mander, Ü., Monti, A., Djomo, S.N., Valentine, J., Walter, K., Zegada-Lizarazu, W., Zenone, T. (2012). Land-use change to bioenergy production in Europe: implications for the greenhouse gas balance and soil carbon. *Global Change Biology: Bioenergy*, *4*(4), 372–391. https://doi.org/10.1111/j.1757-1707.2011.01116.x

Dresner, M., Handelman, C., Braun, S., & Rollwagen-Bollens, G. (2015). Environmental identity, pro-environmental behaviors, and civic engagement of volunteer stewards in Portland area parks. *Environmental Education Research*, *21*(7), 991–1010. https://doi.org/10.1080/13504622.2014.964188

Dubbeling, M. (2014) Integrating Urban and Peri-urban Agriculture and Forestry (UPAF) in city climate change strategies. 18 pp. https://www.ruaf.org/sites/default/files/Final report Urban agriculture and City Climate Change strategies programme June 12014.pdf.

Edelenbosch, O. Y., McCollum, D. L., van Vuuren, D. P., Bertram, C., Carrara, S., Daly, H., Fujimori, S., Kitous, A., Kyle, P., Ó Broin, E., Karkatsoulis, P., Sano, F. (2017). Decomposing passenger transport futures: comparing results of global integrated assessment models. *Transportation Research Part D: Transport and Environment*, *55*, 281–293. https://doi.org/10.1016/j.trd.2016.07.003

Edenhofer, O., Madruga, R. P., Sokona, Y., Seyboth, K., Matschoss, P., Kadner, S., Zwickel, T., Eickemeier, P., Hansen, G., Schlömer, S., von Stechow, C. (2011). *Renewable energy sources and climate change mitigation: special report of the intergovernmental panel on climate change*. https://doi.org/10.1017/CBO9781139151153

Ellis, F. (1998). Household strategies and rural livelihood diversification. *Journal of Development Studies*, *35*(1), 1–38. https://doi.org/10.1080/00220389808422553

Ellis, F. (2008). The determinants of rural livelihood diversification in developing countries. *Journal of Agricultural Economics*, *51*(2), 289–302. https://doi.org/10.1111/j.1477-9552.2000.tb01229.x

Erb, K.-H., Kastner, T., Plutzar, C., Bais, A. L. S., Carvalhais, N., Fetzel, T., Gingrich, S., Haberl, H., Lauk, C., Niedertscheider, M., Pongratz, J., Thurner, M., Luyssaert, S. (2017). Unexpectedly large impact of forest management and grazing on global vegetation biomass. *Nature*, *553*(7686), 73–76. https://doi.org/10.1038/nature25138

Eriksson, L. O., Gustavsson, L., Hänninen, R., Kallio, M., Lyhykäinen, H., Pingoud, K., Pohjola, J., Sathre, R., Solberg, B., Svanaes, J., Valsta, L. (2012). Climate change mitigation through increased wood use in the European construction sector—towards an integrated modelling framework. *European Journal of Forest Research*, *131*(1), 131–144. https://doi.org/10.1007/s10342-010-0463-3

Esteves, T., Kirkby, M., Shakesby, R., Ferreira, A., Soares, J., Irvine, B., Ferreira, C.S., Coelho, C.O., Bento, C.P., Carreiras, M. (2012). Mitigating land degradation caused by wildfire: application of the PESERA model to fire-affected sites in central Portugal. *Geoderma*, *191*, 40–50. Retrieved from https://www.sciencedirect.com/science/article/pii/S001670611200016X

FAO. (2006). *Fire management: voluntary guidelines. Principles and strategic actions.* Rome, Italy.

Favero, A., & Mendelsohn, R. (2014). Using markets for woody biomass energy to sequester carbon in forests. *Journal of the Association of Environmental and Resource Economists*, *1*(1/2), 75–95. https://doi.org/10.1086/676033

Fenton, A., Paavola, J., & Tallontire, A. (2017). The role of microfinance in household livelihood adaptation in Satkhira District, Southwest Bangladesh. *World Development*, *92*, 192–202. https://doi.org/10.1016/j.worlddev.2016.12.004

Fuss, S., Jones, C. D., Kraxner, F., Peters, G. P., Smith, P., Tavoni, M., Van Vuuren, D. P., Canadell, J. G., Jackson, R. B., Milne, J., Moreira, J. R., Nakicenovic, N., Sharifi, A., Yamagata, Y. (2016). Research priorities for negative emissions. *Environmental Research Letters*, *11*(11), 115007. https://doi.org/10.1088/1748-9326/11/11/115007

Gao, B., Huang, T., Ju, X., Gu, B., Huang, W., Xu, L., Rees, R.M., Powlson, D. S., Smith, P., Cui, S. (2018). Chinese cropping systems are a net source of greenhouse gases despite soil carbon sequestration. *Global Change Biology*. https://doi.org/10.1111/gcb.14425

Garnett, T. (2011). Where are the best opportunities for reducing greenhouse gas emissions in the food system (including the food chain)? *Food Policy*, *36*, S23–S32. Retrieved from https://www.sciencedirect.com/science/article/pii/S0306919210001132

Garnett, T., Appleby, M. C., Balmford, A., Bateman, I. J., Benton, T. G., Bloomer, P., Burlingame, B., Dawkins, M., Dolan, L., Fraser, D., Herrero, M., Hoffmann, I., Smith, P., Thornton, P. K., Toulmin, C., Vermeulen, S. J., Godfray, H. C. J. (2013). Sustainable intensification in agriculture: premises and policies. *Science*, *341*(6141), 33–34. https://doi.org/10.1126/science.1234485

Garrity, D. (2012). Agroforestry and the future of global land use. In *Agroforestry-The future of global land use* (pp. 21–27). Springer.

Garschagen, M. (2016). Decentralizing urban disaster risk management in a centralized system? Agendas, actors and contentions in Vietnam. *Habitat International*, *52*, 43–49. https://doi.org/10.1016/j.habitatint.2015.08.030

Gibson, J., Boe-Gibson, G., & Stichbury, G. (2015). Urban land expansion in India 1992–2012. *Food Policy*, *56*, 100–113. https://doi.org/10.1016/J.FOODPOL.2015.08.002

Göbel, C., Langen, N., Blumenthal, A., Teitscheid, P., & Ritter, G. (2015). Cutting food waste through cooperation along the food supply chain. *Sustainability*, *7*(2), 1429–1445. Retrieved from http://www.mdpi.com/2071-1050/7/2/1429/htm

Godfray, H. C. J., Beddington, J. R., Crute, I. R., Haddad, L., Lawrence, D., Muir, J. F., Pretty, J., Robinson, S., Thomas, S. M., Toulmin, C. (2010). Food security: the challenge of feeding 9 billion people. *Science*, *327*(5967), 812–818. https://doi.org/10.1126/science.1185383

Goldstein, B., Hauschild, M., Fernandez, J., & Birkved, M. (2016). Testing the environmental performance of urban agriculture as a food supply in northern climates. *Journal of Cleaner Production*, *135*, 984–994. Retrieved from https://www.sciencedirect.com/science/article/pii/S0959652616308952

Gunatilake, H., Roland-Holst, D., & Sugiyarto, G. (2014). Energy security for India: biofuels, energy efficiency and food productivity. *Energy Policy*, *65*, 761–767. Retrieved from https://www.sciencedirect.com/science/article/pii/S0301421513010732

Guo, J., Wang, B., Wang, G., Wu, Y., & Cao, F. (2018). Vertial and seasonal variations of soil carbon pools in ginkgo agroforestry systems in eastern China. *Catena*, *171*, 450–459. https://doi.org/10.1016/j.catena.2018.07.032

Gustavsson, J., Cederberg, C., Sonesson, U., van Otterdijk, R., & Meybeck, A. (2011). Global food losses and food waste - extent, causes and prevention. In *SAVE FOOD: An initiative on Food Loss and Waste Reduction*. https://doi.org/10.1098/rstb.2010.0126

Haberl, H., Erb, K. H., Krausmann, F., Bondeau, A., Lauk, C., Müller, C., Plutzar, C., Steinberger, J. K. (2011). Global bioenergy potentials from agricultural land in 2050: sensitivity to climate change, diets and yields. *Biomass and Bioenergy*. https://doi.org/10.1016/j.biombioe.2011.04.035

Haggblade, S., Me-Nsope, N. M., & Staatz, J. M. (2017). Food security implications of staple food substitution in Sahelian West Africa. *Food Policy*, *71*, 27–38. https://doi.org/10.1016/J.FOODPOL.2017.06.003

Hammill, A., Matthew, R., & McCarter, E. (2008). Microfinance and climate change adaptation. *IDS Bulletin*, *39*(4), 113–122.

Hansen, M., Potapov, P., Moore, R., Hancher, M., Turubanova, S., Tyukavina, A., Thau, D., Stehman, S. V., Goetz, S. J., Loveland, T. R., Kommareddy, A., Egorov, A., Chini, L., Justice, C. O., Townshend, J. (2013). High-Resolution Global Maps of 21st-Century Forest Cover Change. *Science*, *342*(6160), 850–853. https://doi.org/10.1126/science.1070656

Havemenn, T., & Muccione, V. (2011). *Mechanisms for agricultural climate change mitigation incentives for smallholders. CCAFS Report no. 6*. Retrieved from https://ccafs.cgiar.org/publications/mechanisms-agricultural-climate-change-mitigation-incentives-smallholders#.XPUP1y3MxE4

Havlík, P., Valin, H., Herrero, M., Obersteiner, M., Schmid, E., Rufino, M. C., Mosnier, A., Thornton. P. K., Böttcher, H., Conant, R. T. (2014). Climate change mitigation through livestock system transitions. *Proceedings of the National Academy of Sciences*, *111*(10), 3709–3714. https://doi.org/10.1073/pnas.1308044111

Hengsdijk, H., & de Boer, W. J. (2017). Post-harvest management and post-harvest losses of cereals in Ethiopia. *Food Security*, *9*(5), 945–958. https://doi.org/10.1007/s12571-017-0714-y

Hertel, T. W. (2015). The challenges of sustainably feeding a growing planet. *Food Security*, *7*(2), 185–198. https://doi.org/10.1007/s12571-015-0440-2

Hodges, R. J., Buzby, J. C., & Bennett, B. (2011). Postharvest losses and waste in developed and less developed countries: opportunities to improve resource use. *Journal of Agricultural Science*. https://doi.org/10.1017/S0021859610000936

Ingram, J., Dyball, R., Howden, M., Vermeulen, S., Ganett, T., Redlingshöfer, B., Guilbert, S., Porter, J. (2016). Food security, food systems, and environmental change. *Solutions Journal*, (May-June), 63–73. Retrieved from https://ora.ox.ac.uk/objects/uuid:e7f84fc6-9a91-4c0f-8ba1-3715e2ccc901/download_file?file_format=pdf&safe_filename=Fea_Ingram_4-20-16.pdf&type_of_work=Journal+article

Iordan, C.-M., Hu, X., Arvesen, A., Kauppi, P., & Cherubini, F. (2018). Contribution of forest wood products to negative emissions: historical comparative analysis from 1960 to 2015 in Norway, Sweden and Finland. *Carbon Balance and Management*, *13*(1), 12. https://doi.org/10.1186/s13021-018-0101-9

ITPS-FAO. (2015). *Status of the world’s soil resources (SWSR)*. Retrieved from http://www.fao.org/3/a-i5199e.pdf

Jantz, P., Goetz, S., & Laporte, N. (2014). Carbon stock corridors to mitigate climate change and promote biodiversity in the tropics. *Nature Climate Change*, *4*(2), 138–142. https://doi.org/10.1038/nclimate2105

Jat, M., Dagar, J., Sapkota, T., Govaerts, B., Ridaura, S., Saharawat, Y., Sharma, RK, Tetarwal, JP, Jat, RK, Hobbs, H., Stirling, C. (2016). Climate change and agriculture: adaptation strategies and mitigation opportunities for food security in South Asia and Latin America. *Advances in Agronomy*, *137*, 127–235. Retrieved from https://www.sciencedirect.com/science/article/pii/S0065211315300055

Jebli, M., & Youssef, S. (2017). The role of renewable energy and agriculture in reducing CO2 emissions: evidence for North Africa countries. *Ecological Indicators*, *74*, 295–301. Retrieved from https://www.sciencedirect.com/science/article/pii/S1470160X16306690

Jiang, Y. (2015). China’s water security: current status, emerging challenges and future prospects. *Environmental Science and Policy*, *54*, 106–125. https://doi.org/10.1016/j.envsci.2015.06.006

Kauppi, P. E., Sandström, V., & Lipponen, A. (2018). Forest resources of nations in relation to human well-being. *PLOS ONE*, *13*(5), e0196248. https://doi.org/10.1371/journal.pone.0196248

Keesstra, S. D., Bouma, J., Wallinga, J., Tittonell, P., Smith, P., Cerdà, A., Montanarella, L., Quinton, J. N., Pachepsky, Y., van der Putten, W. H., Bardgett, R. D., Moolenaar, S., Mol, G., Jansen, B., Fresco, L. O. (2016). The significance of soils and soil science towards realization of the United Nations Sustainable Development Goals. *SOIL*, *2*, 111–128. https://doi.org/10.5194/soil-2-111-2016

Keesstra, S., Nunes, J., Novara, A., Finger, D., Avelar, D., Kalantari, Z., & Cerdà, A. (2018). The superior effect of nature based solutions in land management for enhancing ecosystem services. *Science of The Total Environment*, *610*–*611*, 997–1009. Retrieved from https://www.sciencedirect.com/science/article/pii/S0048969717320752

Kemper, J. (2015). Biomass and carbon dioxide capture and storage: a review. *International Journal of Greenhouse Gas Control*, *40*, 401–430. https://doi.org/10.1016/j.ijggc.2015.06.012

Kissinger, M., Sussmann, C., & Dorward, C. (2018). Local or global: a biophysical analysis of a regional food system. *Food Systems*. Retrieved from https://www.cambridge.org/core/journals/renewable-agriculture-and-food-systems/article/local-or-global-a-biophysical-analysis-of-a-regional-food-system/BEB1826C608FC06CE6CD221D25C1EA6D

Kline, K. L., Msangi, S., Dale, V. H., Woods, J., Souza, G. M., Osseweijer, P., Clancy, J. S., Hilbert, J. A., Johnson, F.X., McDonnell, P. C., Mugera, H. K. (2017). Reconciling food security and bioenergy: priorities for action. *GCB Bioenergy*. https://doi.org/10.1111/gcbb.12366

Kloppenberg, J. (2010). Impeding dispossession, enabling repossession: biological open source and the recovery of seed sovereignty. *Journal of Agrarian Change*, *10*(3), 367–388. https://doi.org/10.1111/j.1471-0366.2010.00275.x

Kongsager, R., Locatelli, B., & Chazarin, F. (2016). Addressing climate change mitigation and adaptation together: a global assessment of agriculture and forestry projects. *Environmental Management*, *57*(2), 271–282. https://doi.org/10.1007/s00267-015-0605-y

Kowalski, J., & Conway, T. (2018). Branching out: the inclusion of urban food trees in Canadian urban forest management plans. *Urban Forestry & Urban Greening*. Retrieved from https://www.sciencedirect.com/science/article/pii/S1618866718300736

Kumar, D., & Kalita, P. (2017). Reducing postharvest losses during storage of grain crops to strengthen food security in developing countries. *Foods*, *6*(1), 8. https://doi.org/10.3390/foods6010008

Labrière, N., Locatelli, B., Laumonier, Y., Freycon, V., & Bernoux, M. (2015). Soil erosion in the humid tropics: a systematic quantitative review. *Agriculture, Ecosystems & Environment*, *203*, 127–139. https://doi.org/10.1016/j.agee.2015.01.027

Lal, R. (2011). Sequestering carbon in soils of agro-ecosystems. *Food Policy*, *36*, S33–S39. Retrieved from https://www.sciencedirect.com/science/article/pii/S0306919210001454

Lal, R. (2016). Soil health and carbon management. *Food and Energy Security*, *5*(4), 212–222. https://doi.org/10.1002/fes3.96

Lal, R., & Moldenhauer, W. C. (1987). Effects of soil erosion on crop productivity. *Critical Reviews in Plant Sciences*, *5*(4), 303–367. https://doi.org/10.1080/07352688709382244

Lamb, A., Green, R., Bateman, I., Broadmeadow, M., Bruce, T., Burney, J., Carey, P., Chadwick, D., Crane, E., Field, R., Goulding, K., Griffiths, H., Hastings, A., Kasoar, T., Kindred, D., Phalan, B., Pickett, J., Smith, P., Wall, E., zu Ermgassen, E. K. H. J., Balmford, A. (2016). The potential for land sparing to offset greenhouse gas emissions from agriculture. *Nature Climate Change*, *6*(5), 488–492. https://doi.org/10.1038/nclimate2910

Lee-Smith, D. (2010). Cities feeding people: an update on urban agriculture in equatorial Africa. *Environment and Urbanization*, *22*(2), 483–499. https://doi.org/10.1177/0956247810377383

Lee, Y., Ahern, J., & Yeh, C. (2015). Ecosystem services in peri-urban landscapes: the effects of agricultural landscape change on ecosystem services in Taiwan’s western coastal plain. *Landscape and Urban Planning*, *139*, 137–148. https://doi.org/10.1016/j.landurbplan.2015.02.023

Lejeune, Q., Davin, E., Gudmundsson, L., Winckler, J., & Seneviratne, S. (2018). Historical deforestation locally increased the intensity of hot days in northern mid-latitudes. *Nature Climate Change*, *8*, 386–390. https://doi.org/10.1038/s41558-018-0131-z

Lewis, K., & Witham, C. (2012). Agricultural commodities and climate change. *Climate Policy*, *12*(sup01), S53–S61. https://doi.org/10.1080/14693062.2012.728790

Lewis, S. L., Edwards, D. P., & Galbraith, D. (2015). Increasing human dominance of tropical forests. *Science*, *349*(6250,), 827–832. https://doi.org/10.1126/science.aaa9932

Li, Y., Zhao, M., Motesharrei, S., Mu, Q., Kalnay, E., & Li, S. (2015). Local cooling and warming effects of forests based on satellite observations. *Nature Communications*, *6*, 6603. https://doi.org/10.1038/ncomms7603

Limpens, J., Berendse, F., Blodau, C., Canadell, J. G., Freeman, C., Holden, J., Roulet, N., Rydin, H., Schaepman-Strub, G. (2008). Peatlands and the carbon cycle: from local processes to global implications – a synthesis. *Biogeosciences*, *5*, 1475–1491. Retrieved from www.biogeosciences.net/5/1475/2008/

Lin, Y., Wijedasa, L. S., & Chisholm, R. A. (2017). Singapore’s willingness to pay for mitigation of transboundary forest-fire haze from Indonesia. *Environmental Research Letters*, *12*(2), 24017. https://doi.org/10.1088/1748-9326/aa5cf6

Little, P. D., Smith, K., Cellarius, B. A., Coppock, D. L., & Barrett, C. (2001). Avoiding disaster: diversification and risk management among East African herders. *Development and Change*, *32*(3), 401–433. https://doi.org/10.1111/1467-7660.00211

Lobell, D., Burke, M., Tebaldi, C., Mastrandrea, M., Falcon, W., & Naylor, R. (2008). Prioritizing climate change adaptation needs for food security in 2030. *Science*, *319*(5863), 607–610. https://doi.org/10.1126/science.1152339

Lotze, H., Lenihan, H., Bourque, B., Bradbury, R., Cooke, R., Kay, M., Kidwell, S., Kirby, M., Peterson, C., Jackson, J. (2006). Depletion, degradation, and recovery potential of estuaries and coastal seas. *Science*, *312*(5781), 1806–1809. https://doi.org/10.1126/science.1128035

Luby, C. H., Kloppenburg, J., Michaels, T. E., & Goldman, I. L. (2015). Enhancing freedom to operate for plant breeders and farmers through open source plant breeding. *Crop Science*, *55*(6), 2481. https://doi.org/10.2135/cropsci2014.10.0708

Lugato, E., Paustian, K., Panagos, P., Jones, A., & Borrelli, P. (2016). Quantifying the erosion effect on current carbon budget of European agricultural soils at high spatial resolution. *Global Change Biology*, *22*(5), 1976–1984. https://doi.org/10.1111/gcb.13198

Luyssaert, S., Marie, G., Valade, A., Chen, Y.-Y., Njakou Djomo, S., Ryder, J., Otto, J., Naudts, K., Lansø, A. S., Ghattas, J., McGrath, M. J. (2018). Trade-offs in using European forests to meet climate objectives. *Nature*, *562*(7726), 259–262. https://doi.org/10.1038/s41586-018-0577-1

Lwasa, S., Mugagga, F., Wahab, B., Simon, D., & Climate, J. C. (2014). Urban and peri-urban agriculture and forestry: transcending poverty alleviation to climate change mitigation and adaptation. *Urban Climate*, *7*, 92–106. https://doi.org/10.1016/j.uclim.2013.10.007

Lwasa, S., Mugagga, F., Wahab, B., Simon, D., Connors, J. P., & Griffith, C. (2015). A meta-analysis of urban and peri-urban agriculture and forestry in mediating climate change. *Current Opinion in Environmental Sustainability*, *13*, 68–73. https://doi.org/10.1016/j.cosust.2015.02.003

Maaroufi, N. I., Nordin, A., Hasselquist, N. J., Bach, L. H., Palmqvist, K., & Gundale, M. J. (2015). Anthropogenic nitrogen deposition enhances carbon sequestration in boreal soils. *Global Change Biology*, *21*(8), 3169–3180. https://doi.org/10.1111/gcb.12904

Mal, S., Singh, R. B., Huggel, C., & Grover, A. (2018). *Introducing linkages between climate change, extreme events, and disaster risk reduction*. https://doi.org/10.1007/978-3-319-56469-2_1

Markandya, A., Sampedro, J., Smith, S. J., Van Dingenen, R., Pizarro-Irizar, C., Arto, I., & González-Eguino, M. (2018). Health co-benefits from air pollution and mitigation costs of the Paris Agreement: a modelling study. *The Lancet Planetary Health*, *2*(3), e126–e133. https://doi.org/10.1016/S2542-5196(18)30029-9

Maskrey, A. (2011). Revisiting community-based disaster risk management. *Environmental Hazards*, *10*(1), 42–52. https://doi.org/10.3763/ehaz.2011.0005

Melamed, M., & Schmale, J. (2016). Sustainable policy—key considerations for air quality and climate change. *Current Opinion in Environmental Sustainability*, *23*, 85–91. Retrieved from https://www.sciencedirect.com/science/article/pii/S1877343516301087

Mercer, J. (2010). Policy arena disaster risk reduction or climate change adaptation: are we reinventing the wheel? *Journal of International Development*. https://doi.org/10.1002/jid

Michelini, L., Principato, L., & Iasevoli, G. (2018). Understanding food sharing models to tackle sustainability challenges. *Ecological Economics*, *145*, 205–217. https://doi.org/10.1016/J.ECOLECON.2017.09.009

Minot, N. (2014). Food price volatility in sub-Saharan Africa: has it really increased? *Food Policy*, *45*, 45–56. https://doi.org/10.1016/J.FOODPOL.2013.12.008

Mohammadi, A., Rafiee, S., Jafari, A., Keyhani, A., Mousavi-Avval, S. H., & Nonhebel, S. (2014). Energy use efficiency and greenhouse gas emissions of farming systems in north Iran. *Renewable and Sustainable Energy Reviews*, *30*, 724–733. https://doi.org/10.1016/J.RSER.2013.11.012

Morduch, J., & Sharma, M. (2002). Strengthening public safety nets from the bottom up. *Development Policy Review*, *20*(5), 569–588. https://doi.org/10.1111/1467-7679.00190

Mosquera‐Losada, M. R., Santiago‐Freijanes, J. J., Pisanelli, A., Rois‐Díaz, M., Smith, J., den Herder, M., Moreno, G., Ferreiro‐Domínguez, N., Malignier, N., Lamersdorf, N., Balaguer, F., Pantera, A., Rigueiro‐Rodríguez, A., Aldrey, J. A., González‐Hernández, M. P., Fernández‐ Lorenzo, J. L., Romero‐Franco, R., Burgess, P. J. (2018). Agroforestry in the European common agricultural policy. *Agroforestry Systems*, *92*, 1117. https://doi.org/10.1007/s10457‐ 018‐0251‐5

Mostofa, K., Liu, C., Zhai, W., Minella, M., Vione, D., Gao, K., Minakata, D., Arakaki, T., Yoshioka, T., Hayakawa, K., Konohira, E., Tanoue, E., Akhand, A., Chanda, A., Wang, B., Sakugawa, H. (2016). Reviews and syntheses: ocean acidification and its potential impacts on marine ecosystems. *Biogeosciences*, *13*, 1767–1786. https://doi.org/10.5194/bg-13-1767-2016

Mundler, P., & Rumpus, L. (2012). The energy efficiency of local food systems: a comparison between different modes of distribution. *Food Policy*, *37*(6), 609–615. https://doi.org/10.1016/J.FOODPOL.2012.07.006

Muratori, M., Calvin, K., Wise, M., Kyle, P., & Edmonds, J. (2016). Global economic consequences of deploying bioenergy with carbon capture and storage (BECCS). *Environmental Research Letters*, *11*(9), 95004. https://doi.org/10.1088/1748-9326/11/9/095004

Mutuo, P. K., Cadisch, G., Albrecht, A., Palm, C. A., & Verchot, L. (2005). Potential of agroforestry for carbon sequestration and mitigation of greenhouse gas emissions from soils in the tropics. *Nutrient Cycling in Agroecosystems*, *71*(1), 43–54. https://doi.org/10.1007/s10705-004-5285-6

Nair, P., & Nair, V. (2014). “Solid–fluid–gas”: the state of knowledge on carbon-sequestration potential of agroforestry systems in Africa. *Current Opinion in Environmental Sustainability*, *6*, 22–27. https://doi.org/10.1016/j.cosust.2013.07.014

Naudts, K., Chen, Y., McGrath, M. J., Ryder, J., Valade, A., Otto, J., & Luyssaert, S. (2016). Europe’s forest management did not mitigate climate warming. *Science*, *351*(6273), 597–601. https://doi.org/10.1126/science.aad7270

Nejad, A. N. (2013). Soil and water conservation for desertification control in Iran. In G. Heshmati & V. Squires (Eds.), *Combating Desertification in Asia, Africa and the Middle East: Proven practices* (pp. 377–400). https://doi.org/10.1007/978-94-007-6652-5_18

Nemet, G. F., Holloway, T., & Meier, P. (2010). Implications of incorporating air-quality co-benefits into climate change policymaking. *Environmental Research Letters*, *5*(1), 14007. https://doi.org/10.1088/1748-9326/5/1/014007

Ngigi, M. W., Mueller, U., & Birner, R. (2017). Gender differences in climate change adaptation strategies and participation in group-based approaches: an intra-household analysis from rural Kenya. *Ecological Economics*, *138*, 99–108. https://doi.org/10.1016/j.ecolecon.2017.03.019

Obersteiner, M., Walsh, B., Frank, S., Havlík, P., Cantele, M., Liu, J., Palazzo, A., Herrero, M., Lu, Y., Mosnier, A., Valin, H., Riahi, K., Kraxner, F., Fritz, S., van Vuuren, D. (2016). Assessing the land resource–food price nexus of the Sustainable Development Goals. *Science Advances*, *2*(9), e1501499. https://doi.org/10.1126/sciadv.1501499

Oliver, T. H., & Morecroft, M. D. (2014). Interactions between climate change and land use change on biodiversity: attribution problems, risks, and opportunities. *Wiley Interdisciplinary Reviews: Climate Change*, *5*(3), 317–335. https://doi.org/10.1002/wcc.271

Osuri, A. M., Ratnam, J., Varma, V., Alvarez-Loayza, P., Hurtado Astaiza, J., Bradford, M., Fletcher, C., Ndoundou-Hockemba, M., Jansen, P. A., Kenfack, D., Marshall, A. R., Ramesh, B. R., Rovero, F., Sankaran, M. (2016). Contrasting effects of defaunation on aboveground carbon storage across the global tropics. *Nature Communications*, *7*(1), 11351. https://doi.org/10.1038/ncomms11351

Palm, C., Blanco-Canqui, H., DeClerck, F., Gatere, L., & Grace, P. (2014). Conservation agriculture and ecosystem services: an overview. *Agriculture, Ecosystems & Environment*, *187*, 87–105. https://doi.org/10.1016/J.AGEE.2013.10.010

Payn, T., Carnus, J., Freer-Smith, P., Kimberley, M., Kollert, W., Liu, S., Orazio, C., Rodriguez, L., Silva, LN Wingfield, M. (2015). Changes in planted forests and future global implications. *Forest Ecology and Management*, *352*, 57–67. https://doi.org/10.1016/j.foreco.2015.06.021

Pellegrini, L., & Tasciotti, L. (2014). Crop diversification, dietary diversity and agricultural income: empirical evidence from eight developing countries. *Canadian Journal of Development Studies / Revue Canadienne D’études Du Développement/ Revue Canadienne D’études Du Développement*, *35*(2), 211–227. https://doi.org/10.1080/02255189.2014.898580

Pelletier, J., Gélinas, N., Skutsch, M., Pelletier, J., Gélinas, N., & Skutsch, M. (2016). The place of community forest management in the REDD+ landscape. *Forests*, *7*(12), 170. https://doi.org/10.3390/f7080170

Perugini, L., Caporaso, L., Marconi, S., Cescatti, A., Quesada, B., De Noblet-Ducoudré, N., House, J. I., Arneth, A. (2017). Biophysical effects on temperature and precipitation due to land cover change. *Environmental Research Letters*. https://doi.org/10.1088/1748-9326/aa6b3f

Peterson, N. D. (2012). Developing climate adaptation: the intersection of climate research and development programmes in index insurance. *Development and Change*, *43*(2), 557–584. https://doi.org/10.1111/j.1467-7660.2012.01767.x

Pimentel, D., Zuniga, R., & Morrison, D. (2005). Update on the environmental and economic costs associated with alien-invasive species in the United States. *Ecological Economics*, *52*(3), 273–288. Retrieved from https://www.sciencedirect.com/science/article/pii/S0921800904003027

Pingoud, K., Ekholm, T., Sievänen, R., Huuskonen, S., & Hynynen, J. (2018). Trade-offs between forest carbon stocks and harvests in a steady state – a multi-criteria analysis. *Journal of Environmental Management*, *210*, 96–103. https://doi.org/doi:10.1016/j.jenvman.2017.12.076.

Poore, J., & Nemecek, T. (2018). Reducing food’s environmental impacts through producers and consumers. *Science*, *360*(6392), 987–992. https://doi.org/10.1126/science.aaq0216

Popp, A., Rose, S. K., Calvin, K., Van Vuuren, D. P., Dietrich, J. P., Wise, M., Stehfest, E., Humpenöder, F., Kyle, P., Van Vliet, J., Bauer, N., Lotze-Campen, H., Klein, D., Kriegler, E. (2014). Land-use transition for bioenergy and climate stabilization: model comparison of drivers, impacts and interactions with other land use based mitigation options. *Climatic Change*, *123*(3–4), 495–509. https://doi.org/10.1007/s10584-013-0926-x

Porter, S. D., Reay, D. S., Higgins, P., & Bomberg, E. (2016). A half-century of production-phase greenhouse gas emissions from food loss & waste in the global food supply chain. *Science of the Total Environment*, *571*, 721–729. https://doi.org/10.1016/j.scitotenv.2016.07.041

Powlson, D. S., Stirling, C. M., Thierfelder, C., White, R. P., & Jat, M. L. (2016). Does conservation agriculture deliver climate change mitigation through soil carbon sequestration in tropical agro-ecosystems? *Agriculture, Ecosystems and Environment*, *220*, 164–174. https://doi.org/10.1016/j.agee.2016.01.005

Putz, F. E., Zuidema, P. A., Synnott, T., Peña-Claros, M., Pinard, M. A., Sheil, D., Vanclay, J.K., Sist, P., Gourlet-Fleury, S., Griscom, B., Palmer, J., Zagt, R. (2012). Sustaining conservation values in selectively logged tropical forests: the attained and the attainable. *Conservation Letters*, *5*(4), 296–303. https://doi.org/10.1111/j.1755-263X.2012.00242.x

Qian, J., Peng, Y., Luo, C., Wu, C., & Du, Q. (2015). Urban land expansion and sustainable land use policy in Shenzhen: a case study of China’s rapid urbanization. *Sustainability*, *8*(1), 16. https://doi.org/10.3390/su8010016

Rakodi, C. (1999). A capital assets framework for analysing household livelihood strategies: implications for policy. *Development Policy Review*, *17*(3), 315–342. https://doi.org/10.1111/1467-7679.00090

Ram, A., Dev, I., Uthappa, A. R., Kumar, D., Kumar, N., Chaturvedi, O. P., Dotaniya, M. L., Meena, B. P. (2017). Reactive nitrogen in agroforestry systems of India. In *The Indian Nitrogen Assessment* (pp. 207–218). Elsevier.

Ramanathan, V., Crutzen, P. J., Kiehl, J. T., & Rosenfeld, D. (2001). Aerosols, climate, and the hydrological cycle. *Science (New York, N.Y.)*, *294*(5549), 2119–2124. https://doi.org/10.1126/science.250.4988.1669

Rengasamy, P. (2006). World-a salinization with emphasis on Australia. *Journal of Experimental Botany*, *57*(5), 1017–1023. https://doi.org/10.1093/jxb/erj108

Revi, A., Satterthwaite, D. E., Aragón-Durand, F., Corfee-Morlot, J., Kiunsi, R. B. R., Pelling, M., Roberts, D.C., Solecki. (2014). Urban areas. In C. . Field, V. R. Barros, D. J. Dokken, K. J. Mach, M. D. Mastrandrea, T. E. Bilir, M. Chatterjee, K. L. Ebi, Y. O. Estrada, R. C. Genova, B. Girma, E. S. Kissel, A. N. Levy, S. MacCracken, P. R. Mastrandrea, L. L. White (Eds.), *Climate Change 2014: Impacts, Adaptation, and Vulnerability. Part A: Global and Sectoral Aspects. Contribution of Working Group II to the Fifth Assessment Report of the Intergovernmental Panel on Climate Change* (pp. 535–612).

Rey Benayas, J. M., Newton, A. C., Diaz, A., & Bullock, J. M. (2009). Enhancement of biodiversity and ecosystem services by ecological restoration: a meta-analysis. *Science*, *325*(5944), 1121–1124. https://doi.org/10.1126/science.1172460

Riahi, K., van Vuuren, D. P., Kriegler, E., Edmonds, J., O’Neill, B. C., Fujimori, S., Bauer, N., Calvin, K., Dellink, R., Fricko, O., Lutz, W., Popp, A., Cuaresma, J. C., Samir, K.C., Leimbach, M., Jiang, L., Kram, T., Rao, S., Emmerling, J., Ebi, K., Hasegawa, T., Havlik, P., Humpenöder, F., Da Silva, L. A., Smith, S., Stehfest, E., Bosetti, V., Eom, J., Gernaat, D., Masui, T., Rogelj, J., Strefler, J., Drouet. L., Krey, V., Luderer, G., Harmsen, M., Takahashi, K., Baumstark, L., Doelman, J. C., Kainuma, M., Klimont, Z., Marangoni, G., Lotze-Campen, H., Obersteiner, M., Tabeau, A., Tavoni, M. (2017). The-a Shared Socioeconomic Pathways and their energy, land use, and greenhouse gas emissions implications: an overview. *Global Environmental Change*, *42*, 153–168. https://doi.org/10.1016/j.gloenvcha.2016.05.009

Ridoutt, B., Sanguansri, P., Bonney, L., Crimp, S., Lewis, G., & Lim-Camacho, L. (2016). Climate change adaptation strategy in the food industry—insights from product carbon and water footprints. *Climate*, *4*(2), 26. https://doi.org/10.3390/cli4020026

Ritzema, R. S., Frelat, R., Douxchamps, S., Silvestri, S., Rufino, M. C., Herrero, M., Giller, K. E., López-Ridaura, S., Teufel, N., Paul, B. K. & van Wijk, M. T. (2017). Is production intensification likely to make farm households food-adequate? A simple food availability analysis across smallholder farming systems from East and West Africa. *Food Security*, *9*(1), 115–131. https://doi.org/10.1007/s12571-016-0638-y

Roberts, K.G., Gloy, B.A., Joseph, S., Scott, N.R., & Lehmann, J. (2009) Life cycle assessment of biochar systems: estimating the energetic, economic, and climate change potential. *Environment, Science and Technology, 44,* 827 – 833. doi.org/10.1021/es902266r.

Rosenstock, T. S., Tully, K. L., Arias-Navarro, C., Neufeldt, H., Butterbach-Bahl, K., & Verchot, L. V. (2014). Agroforestry with N2-fixing trees: sustainable development’s friend or foe? *Current Opinion in Environmental Sustainability*. https://doi.org/10.1016/j.cosust.2013.09.001

Rulli, M., Bozzi, S., Spada, M., Bocchiola, D., & Rosso, R. (2006). Rainfall simulations on a fire disturbed Mediterranean area. *Journal of Hydrology*, *327*(3–4), 323–338. Retrieved from https://www.sciencedirect.com/science/article/pii/S0022169405006189

Sain, G., Loboguerrero, A. M., Corner-Dolloff, C., Lizarazo, M., Nowak, A., Martínez-Barón, D., & Andrieu, N. (2017). Costs and benefits of climate-smart agriculture: the case of the Dry Corridor in Guatemala. *Agricultural Systems*, *151*, 163–173. https://doi.org/10.1016/J.AGSY.2016.05.004

Sánchez, J., Curt, M. D., & Fernández, J. (2017). Approach to the potential production of giant reed in surplus saline lands of Spain. *GCB Bioenergy*. https://doi.org/10.1111/gcbb.12329

Santiago‐Freijanes, J. J., Rigueiro‐Rodríguez, A., J.A., A., Moreno, G., den Herder, M., Burgess, P., & Mosquera‐Losada, M. R. (2018). Understanding agroforestry practices in Europe through landscape features policy promotion. *Agroforestry Systems*, *92*(4), 1105–1115. https://doi.org/10.1007/s10457-018-0212-z

Sapkota, T., Shankar, V., Rai, M., Jat, M., Stirling, C., Singh, L., Jat, H.S., Grewal, M. S. (2017). Reducing global warming potential through sustainable intensification of basmati rice-wheat systems in India. *Sustainability*, *9*(6), 1044. https://doi.org/10.3390/su9061044

Scasta, J., Thacker, E., Hovick, T., Engle, D., Allred, B., Fuhlendorf, S., & Weir, J. (2016). Patch-burn grazing (PBG) as a livestock management alternative for fire-prone ecosystems of North America. *Renewable Agriculture and Food Systems*, *31*(6), 550–567. Retrieved from https://www.cambridge.org/core/journals/renewable-agriculture-and-food-systems/article/patchburn-grazing-pbg-as-a-livestock-management-alternative-for-fireprone-ecosystems-of-north-america/FA50DEED8C4522102CD00B09B539C8F3

Schipper, L., & Pelling, M. (2006). Disaster risk, climate change and international development: scope for, and challenges to, integration. *Disasters*, *30*(1), 19–38. https://doi.org/10.1111/j.1467-9523.2006.00304.x

Schuiling, R. D., & Krijgsman, P. (2006). Enhanced weathering: an effective and cheap tool to sequester CO2. *Climatic Change*, *74*(1–3), 349–354. https://doi.org/10.1007/s10584-005-3485-y

Schjønning, P., Jensen, J.L., Bruun, S., Jensen, L.S., Christensen, B.T., Munkholm, L.J., Oelofse, M., Baby, S. & Knudsen, L. (2018) The role of soil organic matter for maintaining crop yields: evidence for a renewed conceptual basis. *Advances in Agronomy, 150,* 35-79. https://doi.org/10.1016/bs.agron.2018.03.001

Searchinger, T. D., Beringer, T., Holtsmark, B., Kammen, D. M., Lambin, E. F., Lucht, W., Raven, P., van Ypersele, J. P. (2018). Europe’s renewable energy directive poised to harm global forests. *Nature Communications*, *9*(1), 10–13. https://doi.org/10.1038/s41467-018-06175-4

Seidl, R., Schelhaas, M. J., Rammer, W., & Verkerk, P. J. (2014). Increasing forest disturbances in Europe and their impact on carbon storage. *Nature Climate Change*, *4*(9), 806–810. https://doi.org/10.1038/nclimate2318

Seinfeld, J. H., & Pandis, S. N. (n.d.). *Atmospheric chemistry and physics: from air pollution to climate change*. Retrieved from https://www.wiley.com/en-us/Atmospheric+Chemistry+and+Physics%3A+From+Air+Pollution+to+Climate+Change%2C+3rd+Edition-p-9781118947401

Shaw, C., Hales, S., Howden-Chapman, P., & Edwards, R. (2014). Health co-benefits of climate change mitigation policies in the transport sector. *Nature Climate Change*, *4*(6), 427–433. https://doi.org/10.1038/nclimate2247

Shcherbak, I., Millar, N., & Robertson, G. P. (2014). Global metaanalysis of the nonlinear response of soil nitrous oxide (N2O) emissions to fertilizer nitrogen. *Proceedings of the National Academy of Sciences of the United States of America*, *111*(25), 9199–9204. https://doi.org/10.1073/pnas.1322434111

Sheahan, M., & Barrett, C. B. (2017). Ten striking facts about agricultural input use in sub-Saharan Africa. *Food Policy*, *67*, 12–25. https://doi.org/10.1016/J.FOODPOL.2016.09.010

Shen, X., Wang, L., Wu, C., Lv, T., Lu, Z., Luo, W., & Li, G. (2017). Local interests or centralized targets? How China’s local government implements the farmland policy of Requisition–Compensation Balance. *Land Use Policy*, *67*, 716–724. https://doi.org/10.1016/J.LANDUSEPOL.2017.06.012

Sida, T. S., Baudron, F., Hadgu, K., Derero, A., & Giller, K. E. (2018). Crop vs. tree: can agronomic management reduce trade-offs in tree-crop interactions? *Agriculture, Ecosystems & Environment*, *260*, 36–46. https://doi.org/10.1016/J.AGEE.2018.03.011

Sims, R., Schaeffer, R., Creutzig, F., Cruz-Núñez, X., D’Agosto, M., Dimitriu, D., Meza, M. J. F., Fulton, L., Kobayashi, S., Lah, O., McKinnon, A., Newman, P., Ouyang, M. Schauer, J. J., Sperling, D., Tiwari, G. (2014). Transport. In O. Edenhofer, R. Pichs-Madruga, Y. Sokona, E. Farahani, S. Kadner, K. Seyboth, A. Adler, I. Baum, S. Brunner, P. Eickemeier, B. Kriemann, J. Savolainen, S. Schlöme, C. von Stechow, T. Zwickel, J. C. Minx (Eds.), *Climate Change 2014: Mitigation of Climate Change. Contribution of Working Group III to the Fifth Assessment Report of the Intergovernmental Panel on Climate Change*. Cambridge, United Kingdom and New York, NY, USA: Cambridge University Press.

Singh, G. (2009). Salinity-related desertification and management strategies: Indian experience. *Land Degradation & Development*, *20*(4), 367–385. https://doi.org/10.1002/ldr.933

Slade, R., Bauen, A., & Gross, R. (2014). Global bioenergy resources. *Nature Climate Change*, *4*, 99–105. https://doi.org/10.1038/nclimate2097

Smith, P. (2008). Land use change and soil organic carbon dynamics. *Nutrient Cycling in Agroecosystems*, *81*(2), 169–178. https://doi.org/10.1007/s10705-007-9138-y

Smith, V. H., & Glauber, J. W. (2012). Agricultural insurance in developed countries: where have we been and where are we going? *Applied Economic Perspectives and Policy*, *34*(3), 363–390. https://doi.org/10.1093/aepp/pps029

Soane, B. D., & van Ouwerkerk, C. (1994). Soil compaction problems in world agriculture. *Developments in Agricultural Engineering*, *11*, 1–21. https://doi.org/10.1016/B978-0-444-88286-8.50009-X

Song, G., Li, M., Fullana-i-Palmer, P., Williamson, D., & Wang, Y. (2017). Dietary changes to mitigate climate change and benefit public health in China. *Science of the Total Environment*, *577*, 289–298. https://doi.org/10.1016/j.scitotenv.2016.10.184

Springer, N. P., Garbach, K., Guillozet, K., Haden, V. R., Hedao, P., Hollander, A. D., Huber, P. R., Ingersoll, C., Langner, M., Lipari, G., Mohammadi, Y., Musker, R., Piatto, M., Riggle, C., Schweisguth, M., Sin, E., Snider, S., Vidic, N., White, A., Brodt, S., Quinn, J. F., Tomich, T. P. (2015). Sustainable sourcing of global agricultural raw materials: assessing gaps in key impact and vulnerability issues and indicators. *PLOS ONE*, *10*(6), e0128752. https://doi.org/10.1371/journal.pone.0128752

Stanturf, J. A., Kant, P., Lillesø, J.-P. B., Mansourian, S., Kleine, M., Graudal, L., & Madsen, P. (2015). Forest landscape restoration as a key component of climate change mitigation and adaptation. In *IUFRO World Series Volume 34*. Retrieved from https://www.iufro.org/publications/series/world-series/article/2015/12/01/world-series-vol-34-forest-landscape-restoration-as-a-key-component-of-climate-change-mitigation/

Steinbach, H. S., & Alvarez, R. (2006). Changes in soil organic carbon contents and nitrous oxide emissions after introduction of no-till in Pampean agroecosystems. *Journal of Environmental Quality*, *35*(1), 3–13. https://doi.org/doi:10.2134/jeq2005.0050

Sternberg, T., & Batbuyan, B. (2013). Integrating the Hyogo framework into Mongolia’s disaster risk reduction (DRR) policy and management. *International Journal of Disaster Risk Reduction*. https://doi.org/10.1016/j.ijdrr.2013.05.003

Tadasse, G., Algieri, B., Kalkuhl, M., & von Braun, J. (2016). Drivers and triggers of international food price spikes and volatility. In l M. Kalkuh, J. von Braun, & M. Torero (Eds.), *Food Price Volatility and Its Implications for Food Security and Policy* (pp. 59–82). https://doi.org/10.1007/978-3-319-28201-5_3

Tan, R., Beckmann, V., van den Berg, L., & Qu, F. (2009). Governing farmland conversion: comparing China with the Netherlands and Germany. *Land Use Policy*, *26*(4), 961–974. https://doi.org/10.1016/J.LANDUSEPOL.2008.11.009

Tao, Y., Li, F., Liu, X., Zhao, D., Sun, X., & Modelling, L. X. (2015). Variation in ecosystem services across an urbanization gradient: a study of terrestrial carbon stocks from Changzhou, China. *Ecological Applications*. Retrieved from https://www.sciencedirect.com/science/article/pii/S0304380015001994

Thomalla, F., Downing, T., Spanger-Siegfried, E., Han, G., & Rockström, J. (2006). Reducing hazard vulnerability: towards a common approach between disaster risk reduction and climate adaptation. *Disasters*, *30*(1), 39–48. https://doi.org/10.1111/j.1467-9523.2006.00305.x

Tian, X., Sohngen, B., Baker, J., Ohrel, S., & Fawcett, A. A. (2018). Will US forests continue to be a carbon sink? *Land Economics*, *94*(1), 97–113.

Tighe, M., Haling, R. E., Flavel, R. J., & Young, I. M. (2012). Ecological succession, hydrology and carbon acquisition of biological soil crusts measured at the micro-scale. *PLoS ONE*, *7*(10), e48565. https://doi.org/10.1371/journal.pone.0048565

Tilman, D., and M. Clark, 2014: Global diets link environmental sustainability and human health. Nature, 515, 518–522, doi:10.1038/nature13959.

Torvanger, A. (2018). Governance-b of bioenergy with carbon capture and storage (BECCS): accounting, rewarding, and the Paris agreement. *Climate Policy*, *0*(0), 1–13. https://doi.org/10.1080/14693062.2018.1509044

Trabucco, A., Zomer, R. J., Bossio, D. A., van Straaten, O., & Verchot, L. V. (2008). Climate change mitigation through afforestation/reforestation: a global analysis of hydrologic impacts with four case studies. *Agriculture, Ecosystems and Environment*, *126*(1–2), 81–97. https://doi.org/10.1016/j.agee.2008.01.015

UNEP. (2017). *The emissions gap report 2017*. Retrieved from https://www.unenvironment.org/resources/emissions-gap-report-2017

Valendik, E. N., Vershovets, S. V., Kisilyahov, E. K., Ivanova, G. A., Bruchanov, A. V., Kosov, I. V., & Goldammer, J. G. (2011). *Tekhnologii kontroliruyemykh vyzhiganiy v lesakh Sibiri: kollektivnaya monografiya [Technologies of controlled burning in forests of Siberia]* (E. S. Petrenko, Ed.). Siberian Federal University, Krasnoyarsk.

van Vuuren, D. P., Hof, A. F., van Sluisveld, M. A. E., & Riahi, K. (2017). Open discussion of negative emissions is urgently needed. *Nature Energy*, *2*(12), 902–904. https://doi.org/10.1038/s41560-017-0055-2

van Vuuren, D. P., Kok, M., Lucas, P. L., Prins, A. G., Alkemade, R., van den Berg, M., Bouwman, L., van der Esch, S., Jeuken, M., Kram, T., Stehfest, E. (2015). Pathways to achieve a set of ambitious global sustainability objectives by 2050: Explorations using the IMAGE integrated assessment model. *Technological Forecasting and Social Change*, *98*, 303–323. https://doi.org/10.1016/j.techfore.2015.03.005

van Vuuren, D. P., Stehfest, E., den Elzen, M. G. J., Kram, T., van Vliet, J., Deetman, S., Isaac, M., Goldewijk, K. K., Hof, A., Beltran, A. M., Oostenrijk, R., van Ruijven, B. (2011). RCP2.6: exploring the possibility to keep global mean temperature increase below 2 degrees C. *Climatic Change*, *109*, 95. https://doi.org/10.1007/s10584-011-0152-3

van Vuuren, D. P., van Soest, H., Riahi, K., Clarke, L., Krey, V., Kriegler, E., Rogelj, J., Schaeffer, M., avoni, M. (2016). Carbon budgets and energy transition pathways. *Environmental Research Letters*, *11*(7), 75002. https://doi.org/10.1088/1748-9326/11/7/075002

VandenBygaart, A. J. (2016). The myth that no-till can mitigate global climate change. *Agriculture, Ecosystems & Environment*, *216*, 98–99. https://doi.org/10.1016/J.AGEE.2015.09.013

Vermeulen, S. J., Campbell, B. M., & Ingram, J. S. I. (2012). Climate change and food systems. *Annual Review of Environment and Resources*, *37*(1), 195–222. https://doi.org/10.1146/annurev-environ-020411-130608

Vignola, R., Harvey, C. A., Bautista-Solis, P., Avelino, J., Rapidel, B., Donatti, C., & Martinez, R. (2015). Ecosystem-based Adaptation for smallholder farmers: definitions, opportunities and constraints. *Agriculture, Ecosystems & Environment*, *211*, 126–132. https://doi.org/10.1016/J.AGEE.2015.05.013

Vilà, M., Espinar, J. L., Hejda, M., Hulme, P. E., Jarošík, V., Maron, J. L., Pergl, J., Schaffner, U., Sun, Y., Pyšek, P. (2011). Ecological impacts of invasive alien plants: a meta-analysis of their effects on species, communities and ecosystems. *Ecology Letters*, *14*(7), 702–708. https://doi.org/10.1111/j.1461-0248.2011.01628.x

Vogel, C., & O’Brien, K. (2006). Who can eat information? Examining the effectiveness of seasonal climate forecasts and regional climate-risk management strategies. *Climate Research*, *33*(1), 111–122. https://doi.org/10.3354/cr033111

Waldron, A., Garrity, D., Malhi, Y., Girardin, C., Miller, D. C., & Seddon, N. (2017). Agroforestry can enhance food security while meeting other Sustainable Development Goals. *Tropical Conservation Science*, *10*, 194008291772066. https://doi.org/10.1177/1940082917720667

Wattnem, T. (2016). Seed laws, certification and standardization: outlawing informal seed systems in the Global South. *The Journal of Peasant Studies*, *43*(4), 850–867. https://doi.org/10.1080/03066150.2015.1130702

Westerling, A., Hidalgo, H., Cayan, D., & Swetnam, T. (2006). Warming and earlier spring increase Western U.S. forest wildfire activity. *Science (New York, N.Y.)*, *313*(5789), 940–943. https://doi.org/10.1126/science.262.5135.885

Whitehead, P. J., Purdon, P., Russell-Smith, J., Cooke, P. M., & Sutton, S. (2008). The management of climate change through prescribed Savanna burning: emerging contributions of indigenous people in Northern Australia. *Public Administration and Development*, *28*(5), 374–385. https://doi.org/10.1002/pad.512

Wild, M., Roesch, A., & Ammann, C. (2012). Global dimming and brightening - evidence and agricultural implications. *CAB Reviews*, *7*(3). https://doi.org/10.1079/PAVSNNR20127003

Wilhelm, M., Blome, C., Bhakoo, V., & Paulraj, A. (2016). Sustainability in multi-tier supply chains: understanding the double agency role of the first-tier supplier. *Journal of Operations Management*, *41*, 42–60. https://doi.org/10.1016/j.jom.2015.11.001

Wodon, Q., & Zaman, H. (2010). Higher food prices in sub-Saharan Africa: poverty impact and policy responses. *The World Bank Research Observer*, *25*(1), 157–176. https://doi.org/10.1093/wbro/lkp018

World Bank. (2011). *Rising global Interest in farmland: can it yield sustainable and equitable benefits?* https://doi.org/10.1596/978-0-8213-8591-3

Wu, W., Hasegawa, T., Ohashi, H., Hanasaki, N., Liu, J., Matsui, T., Fujimori, S., Masui, T., Takahashi, K. (2019). Global advanced bioenergy potential under environmental protection policies and societal transformation measures. *GCB Bioenergy*, gcbb.12614. https://doi.org/10.1111/gcbb.12614

Xu, Y., & Ramanathan, V. (2017). Well below 2 °C: mitigation strategies for avoiding dangerous to catastrophic climate changes. *Proceedings of the National Academy of Sciences*, *114*(39), 10315‐10323. https://doi.org/10.1073/pnas.1618481114

Xu, Y., Zaelke, D., Velders, G. J. M., & Ramanathan, V. (2013). The role of HFCs in mitigating 21st century climate change. *Atmospheric Chemistry and Physics*, *13*, 6083–6089. https://doi.org/10.5194/acp-13-6083-2013

Yirdaw, E., Tigabu, M., & Monge, A. (2017). Rehabilitation of degraded dryland ecosystems – review. *Silva Fennica*, *51*(1B), 1673. https://doi.org/10.14214/sf.1673

Yong, D., & Peh, K. (2016). South-east Asia’s forest fires: blazing the policy trail. *Oryx*, *50*(2), 207–212. Retrieved from https://www.cambridge.org/core/journals/oryx/article/southeast-asias-forest-fires-blazing-the-policy-trail/CA89A255B7D7DA310F2F02268178D33A
